# Electrochemical Corrosion and Safety Hazards in Sustainable Batteries: Corrosion Mechanisms, Safety Challenges, and Protection

**DOI:** 10.1007/s40820-026-02178-9

**Published:** 2026-04-15

**Authors:** Chandrabhan Verma, Imad Barsoum, Akram Alfantazi, Kyong Yop Rhee

**Affiliations:** 1 Department of Chemical and Petroleum Engineering, Khalifa University of Science and Technology, P.O. Box 127788, Abu Dhabi, United Arab Emirates; 2 Emirates Nuclear Technology Center (ENTC), Khalifa University, 12788 Abu Dhabi, United Arab Emirates; 3 Department of Mechanical and Nuclear Engineering, Khalifa University of Science and Technology, Abu Dhabi, 12788 United Arab Emirates; 4 Department of Mechanical Engineering, College of Engineering, Kyung Hee University, Yongin, 445-701 South Korea

**Keywords:** Electrochemical corrosion, Sustainable energy storage, Metal-anode batteries, Interfacial engineering, Corrosion inhibitors

## Abstract

The swift transition to sustainable energy has heightened demand for high-performance, safe, and environmentally responsible battery technologies. Zn₋, Mg₋, Na₋, Al₋, Fe₋, organic, and bio-based systems offer several advantages over traditional resource-intensive and toxic alternatives. However, their practical implications are significantly challenged by their susceptibility to electrochemical corrosion, which adversely affects their efficiency, longevity, safety, recyclability, and reversibility. Corrosion is one of the most significant and persistent barriers to the development of next-generation energy storage systems. This review comprehensively presents unified mechanisms of corrosion across diverse sustainable battery systems, with a detailed account of pitting, uniform, galvanic, intergranular, and passivation-related degradation pathways. The article presents a unique comparison of the degradation mechanisms of Zn, Al, Mg, and other anodes in different electrolytes. Corrosion mitigation strategies, including surface passivation, surface engineering, alloying, use of surfactants and polymer-based films, ionic liquids, deep eutectic solvents, metal–organic frameworks, heterocycles, and bio-based multifunctional corrosion inhibitors, have been comprehensively surveyed. These inhibitors suppress the increase in cycle life, achieving inhibition efficiencies of over 90%. Lastly, the review highlights the design of molecular-level corrosion inhibitors, interfacial engineering, real-time corrosion testing, advanced electrolytes, and forward-looking directions, all of which are essential to the development of sustainable, stable energy storage systems.

## Highlights

- This review provides the first comprehensive collection of electrochemical corrosion mechanisms in Zn-, Al-, Mg-, Na-, organic-, and bio-based batteries, highlighting their safety and hazardous effects of corrosion in sustainable batteries.
- This review uniquely presents the connection of electrochemical corrosion with dendrite formation, hydrogen evolution, impedance growth, recycling challenges, capacity fading, and safety hazards.
- This provides a next-generation solution, such as corrosion inhibitors, bio-derived additives, metal–organic frameworks, deep eutectic solvents, ionic liquids, gel electrolytes, and interfacial and surface engineering approaches.

## Graphical abstract

**Figure:**
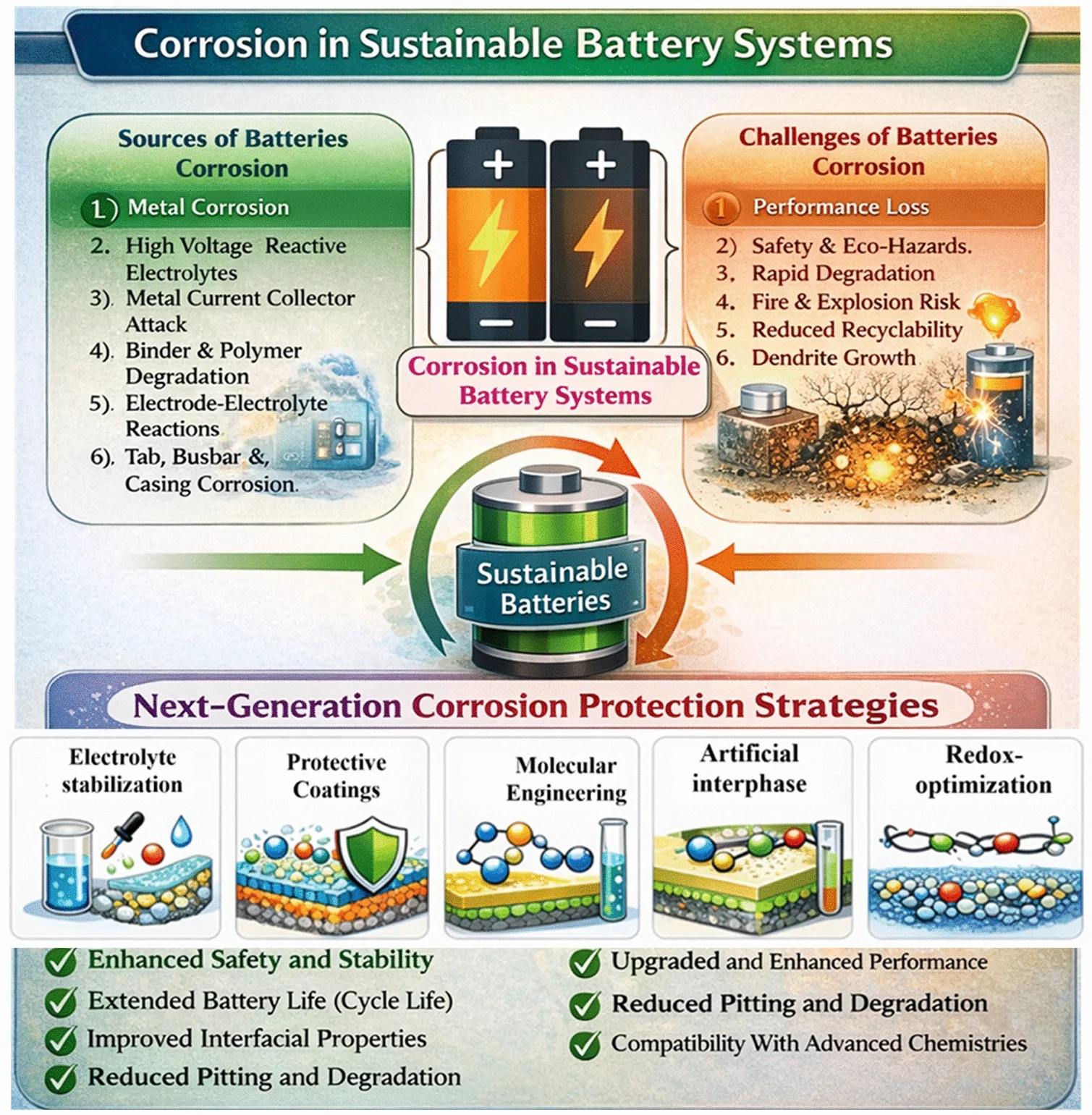

## Introduction

### Safe and Sustainable Batteries: Need for Clean and Green Energy Transition

Batteries are electrochemical devices that change stored chemical energy into electrical energy through redox reactions [1, 2]. They consist of negative (anode) and positive (cathode) electrodes, a separator that prevents direct electrical contact between the electrodes, and an electrolyte that allows ions to move between the electrodes during charging and discharging. By storing wind and solar energy, they serve as renewable energy resources for on-demand use. They are widely used in electric vehicles (EVs) to minimize greenhouse gas emissions from transport and achieve a net-zero emissions (NZE) target by 2050 [3, 4]. The urgent climate goals, such as reducing CO_2_ emissions, fossil fuel use, and improving the economy, have driven research and development (R&D) into battery technology. In the power sector, to achieve NZEs by 2050, global energy storage capacity needs to be increased sixfold to 1,500 GW, with batteries accounting for approximately 90% of this increase [5]. They have also reduced the reliance on fossil fuels and enhanced energy security. According to the IEA's 2024 report, the development of batteries alone in the transport sector could increase battery demand by nearly sevenfold by 2030, representing a significant shift that could displace over 8 million barrels of oil/day compared to the fossil fuel baseline. According to this report, the worldwide battery manufacturing size stood at nearly ~ 3 TWh in 2024 and is projected to reach ~ 6.5 TWh by 2030. Despite significant progress, scientists and engineers continue to work diligently to develop more affordable, energy-efficient, and environmentally friendly substitutes. This surge has led to noticeable improvements in charging speed, battery lifespan, and energy density.

**Fig. 1:**
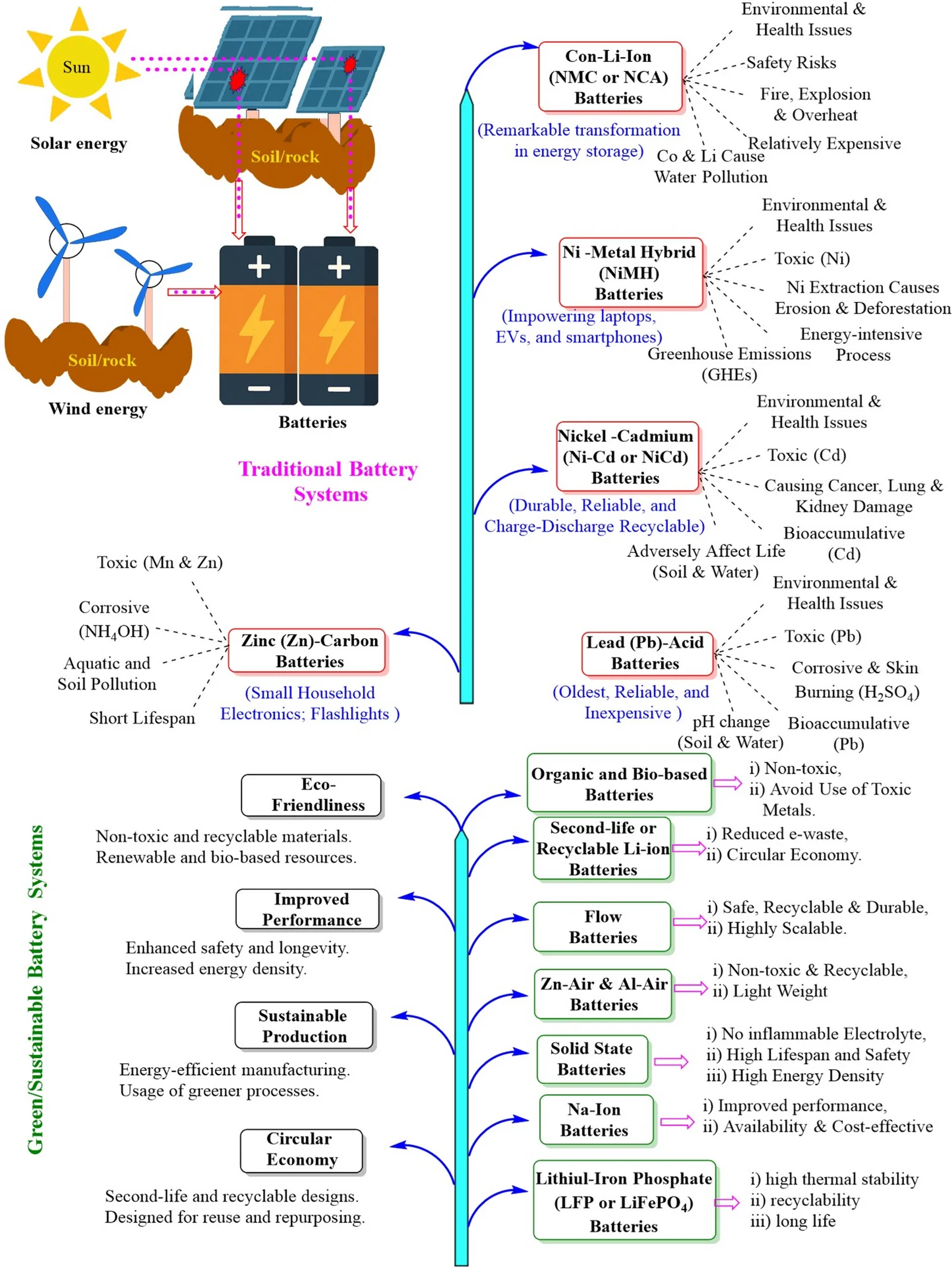
Schematic illustration of challenges and drawbacks of traditional battery systems and opportunities of sustainable battery systems. The figure represents the comparative representation of (upper) conventional battery systems, including Ni–Cd, Pb-acid, Co/Ni-rich Li-ion, and NiMH, with particular emphasis on main challenges such as environmental burden, toxicity, dependence on raw materials, mining and disposal, limited recycling, and safety risks; and (lower) emerging (next-generation) battery systems, such as Zn-, Al-, Mg-, Fe-, Na-based, and organic/bio-based, with particular advantages of reduced toxicity, high compatibility, resource abundance, scalability and improved safety. The figure also shows that, despite several advantageous features of emerging battery systems, corrosion-mediated electrode degradation remains a significant challenge that adversely affects battery durability, stability, and performance

Although traditional batteries have been widely used for decades, they pose significant health and environmental challenges due to their toxic materials (Fig. 1) [6, 7]. Lead-acid batteries, which are among the oldest, most reliable, and least expensive types of batteries, contain a highly poisonous lead electrode and a corrosive H_2_SO_4_ electrolyte [8]. If they are not suitably recycled or processed, they contaminate and adversely affect aquatic and soil life. Besides the toxicity concerns of lead, the spillage or leakage of highly corrosive acid causes metallic corrosion, skin burns, and damages the ecosystem by altering the pH of soil and water. Likewise, nickel–cadmium (Ni–Cd or NiCd) batteries are associated with several serious environmental and health issues, although they are relatively durable and can withstand many charge–discharge cycles [9, 10]. Cd is a highly toxic and cancer-causing element, adversely affecting the aquatic and soil life, and it accumulates in plants and animals after entering the food chain [11]. Cd also causes kidney and lung damage. Nickel-metal-hybrid (NiMH) batteries are relatively safer than Pb- and Cd-based batteries; nevertheless, they pose significant environmental challenges [10, 12].

The extraction, processing, and purification of the rare earth and Ni elements can cause severe ecosystem damage through mining waste contamination of soil and water, causing deforestation and soil erosion [13, 14]. The mining process is energy- and water-consuming, contributing to greenhouse gas emissions. Conventional Li-ion batteries, mainly composed of Ni, Mg, and Co (NMC) or Ni, Co, and Al (NCA), have undergone a remarkable transformation in current knowledge, powering laptops, EVs, and smartphones. However, they are associated with serious sustainability challenges, as mining-based extraction and processing of Co and Li result in water and soil pollution [15, 16]. Li-ion batteries also pose safety risks, as they can catch fire, explode, and overheat. Zinc-carbon (ZnC) batteries are frequently used in small household electronics such as flashlights. The toxicity concerns of ZnC batteries arise from corrosive chemicals and toxic metals, including NH_4_Cl, MnO_2_, and Zn. These substances can leak, especially after disposal, to cause aquatic and oil pollution. The environmental challenges and their short lifespan make them unsuitable alternatives for modern sustainability goals, despite the convenience and inexpensiveness.

**Fig. 2:**
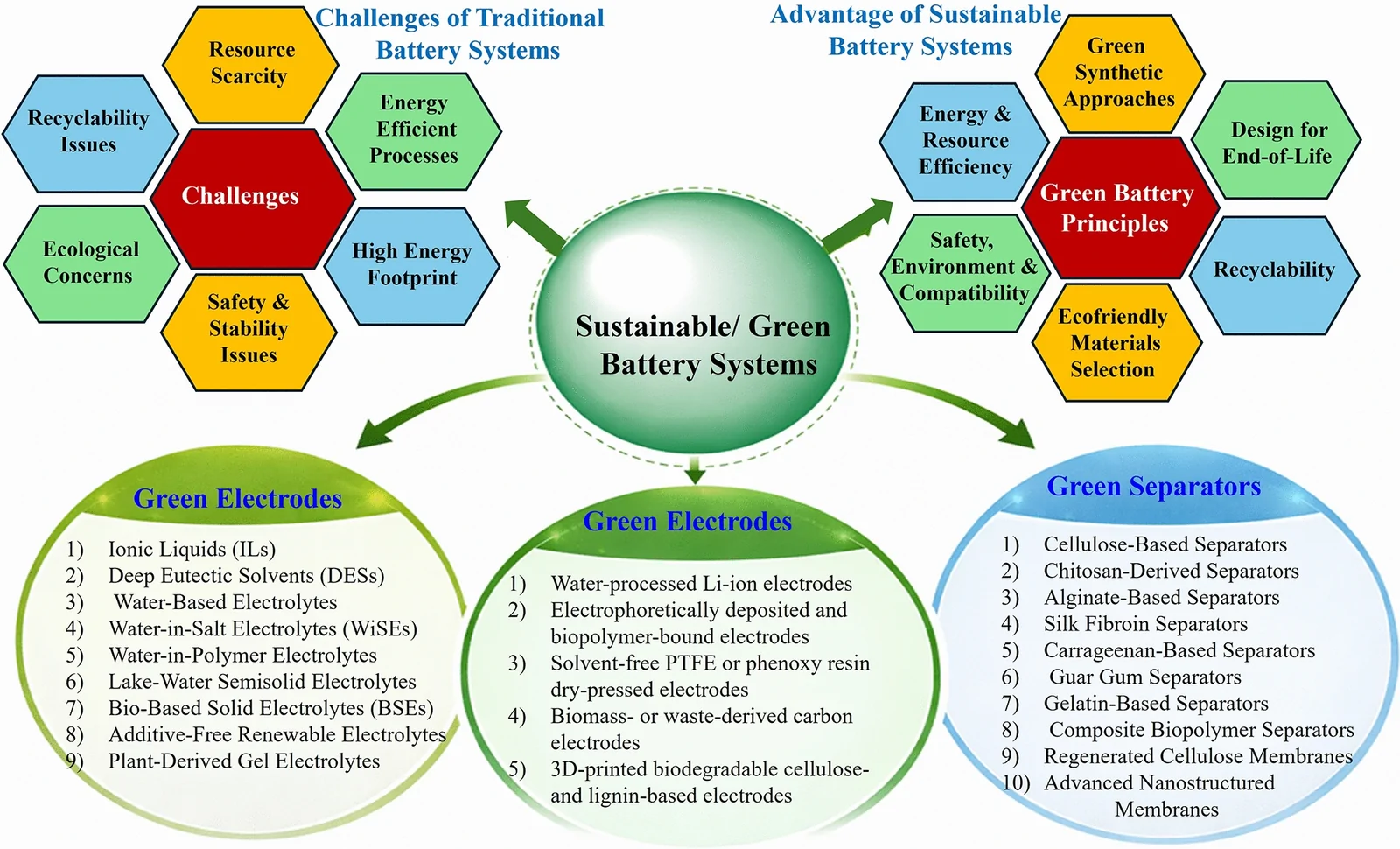
Schematic presentation of the conceptual framework linking (i) main challenges and shortcomings of traditional batteries, such as safety risks, toxicity, environmental burden, and limited recyclability; (ii) green battery design principles, including circular economy compatibility, recyclability, resource abundance, environmental safety, reduced risks and toxicity; (iii) material-level use strategies such as advanced electrolytes (e.g., ILs, DESs and gel systems), sustainable electrodes (Zn, Al, Mg, Na, Fe, and organic systems), and nanostructured and bio-based separators. This figure shows that corrosion remains a critical shortcoming affecting the durability, stability, and performance of sustainable batteries

Recently, demand for environmentally friendly and sustainable energy storage technologies has increased significantly as the shift toward renewable energy systems continues [17, 18]. Efforts are underway to minimize the carbon footprint, promote recycling, and encourage EV adoption, thereby enabling the emergence of eco-friendly, socially responsive, economically and technically viable alternatives. Sustainable battery strategies utilize renewable materials, energy-efficient and eco-conscious syntheses, and non-toxic components. These strategies promote biodegradability, recyclability, the circular economy, and sustainable sourcing, with enhanced renewability, safety, performance, and sustainability. The use of bio-inspired systems has shown great potential in bio-electrochemical and biomolecule-based batteries, imitating biological energy conversion [19–21]. These safe, biodegradable, and sustainable substitutes have already demonstrated great potential in wearable devices, environmental monitoring, biomedical applications, and agricultural systems. Recent studies have explored the use of promising biomolecules, such as lawsone, juglone, and flavins, as sustainable redox-active agents [22]. These naturally derived renewable biomasses eliminate toxic metals and deliver high energy density with excellent sustainability and biodegradability. The use of efficient and sustainable electrolytes, such as deep eutectic solvents (DESs), ionic liquids (ILs), renewable and bio-based polymers, additive-free water-based systems, and gel-based systems, has emerged as another sustainable strategy (Fig. 2) [23, 24]. Recently, the use of lignocellulose-based polymer electrolytes derived from biomass has also advanced substantially, owing to their enhanced interfacial compatibility, mechanical integrity, and controlled ion transport [25]. As illustrated in Fig. 2, the transition from convectional to next-generation sustainable batteries can be understood through a structured framework (Table 1): Challenges and shortcoming of traditional batteries (safety risks, toxicity, environmental burden, limited recyclability) motivate specific green or sustainable design principles (circular economy compatibility, recyclability, resource abundance, ecological safety, reduced risks and toxicity) which are then implemented through the targeted material-level strategies such as advanced electrolytes (e.g., ILs, DESs, and gel systems), sustainable electrodes (Zn, Al, Mg, Na, Fe, and organic systems), and nanostructured and bio-based separators. Nevertheless, despite these improvements, corrosion remains a critical shortcoming that affects the durability, stability, and performance of sustainable batteries.

**Table 1:** A comparative summary of challenges of traditional battery systems, sustainability of green design principles, sustainable alternatives, and their advantages

S/N	Traditional battery challenges	Green/ sustainable design principles	Sustainable alternatives	Advantages of sustainable alternatives
1	Toxic electrodes (Co, Pd, Cd)-environmental and health risks	Replace with more sustainable alternatives	Zn, Al, Mg, Na, Fe-based metals, organic and bio-based systems	Improved environmental computability, reduced toxicity, and risks
2	Dependence on dangerous raw materials (rare earth elements, Ni, Co)	Replace with more abundant metal chemistries	Co/Ni-free cathodes, Zn-ion, Na-ion, Fe-ion, etc	Supply chain stability and resource abundance
3	High carbon footprint (due to mining-related processes)	Avoid mining-related processes to reduce the environmental impact	Recyclable separators, aqueous systems, and second-life battery uses	Circular economy mixing and minimizing ecological risks
4	Traditional organic electrolytes: flammability and thermal runway risks	Replace flammable electrolytes with less volatile ones	Solid-battery systems, aqueous systems, polymer gel electrolyte, and DESs/ILs electrolyte	Enhanced thermal and intrinsic safety
5	Disposal problem (hazardous) and limited recyclability	Focusing on metal recovery strategies	Recyclable electrodes (Metal) in an aqueous system	Enhanced material recovery and cycling
6	Performance in stability-side reactions and corrosion	Material design, surface engineering, and electrolyte modification	Alloying, corrosion inhibitors, functional additives, protective coatings, etc	Improved stability, cycle life, and performance

These strategies also include using green and sustainable separators, especially those derived from natural resources, such as chitosan- and cellulose-based membranes, bio-based separators, and advanced nanostructured separators [26, 27]. Nowadays, lithium iron phosphate (LFP or LiFePO_4_) batteries, which feature Co- and Ni-free compositions, offer numerous benefits, including high thermal stability, recyclability, and long life [28, 29]. Similarly, Na-ion batteries improve environmental performance due to their significant availability, non-toxicity, reduced production costs, especially at large scale, which minimizes resource strain and affordability [30]. Solid-state batteries eliminate the use of flammable liquid-based electrolytes, thereby increasing lifespan, energy density, and safety [31, 32]. These properties make them useful alternatives for next-generation EV batteries. Zn-air and Al-air batteries, which utilize oxygen from the air as a reactant, produce non-toxic, recyclable, and lightweight power sources useful for storage and backup systems [33, 34]. Flow batteries (organic or vanadium) offer safe, recyclable, durable, and highly scalable alternatives for renewable energy grids. Second-life or recyclable Li-ion batteries reduce adverse effects by supporting the circular economy and extending the usefulness of EV batteries, thereby reducing e-waste [35, 36]. Organic and bio-based batteries are composed of carbon and bio-based polymers, thereby avoiding the use of toxic heavy metals [37]. For the second-life use and safe recycling of Li-ion batteries, efficient discharge methods are essential. The redox-couple electrolytes, such as Fe(II)/Fe(III) systems, can be fully discharged without producing toxic gases. Obviously, this approach reduces the hazards and reasonably protects recycling workers. In the present article, the term “sustainable batteries” refers to emerging battery technologies that have emerged as alternatives to conventional toxic and critical-material-intensive battery systems such as Ni–Cd, Pb-acid, and Co-/Ni-rich Li-ion (NMC/NCA) batteries. The sustainable battery systems include aqueous and metal-anode batteries based on earth-abundant elements, such as Al, Zn, Mg, Na, and Fe, as well as selected organic or bio-based electrode systems. The sustainable battery systems offer several advantages over traditional alternatives, including safety, resource availability, and large-scale compatibility. This article aims to understand the corrosion mechanisms in sustainable battery systems, their associated challenges, consequences, and mitigation strategies.

### Electrochemical Corrosion in Sustainable Batteries: Significance, Mechanisms, and Environmental Factors

Corrosion is defined as the degradation of metallic materials due to their interactions with environmental constituents [38]. In battery systems, corrosion poses a severe problem to the safety, long-term performance, and cost efficiency of the energy storage technologies, which are the most significant pillars of NZE 2050 [39, 40]. Corrosion adversely affects efficiency and lifespan, leading to undesirable replacement. This could increase the material cost and consumption, raise the carbon footprint, and reduce recyclability. Corrosion-related damages or failures can compromise the integrity and stability of the grid, as well as its reliability, discouraging confidence in clean energy transformations. Numerous battery components, including metallic connectors (e.g., Al in cathodes and Cu in anodes) and current collectors, are highly susceptible to electrochemical corrosion, particularly in humid, high-voltage conditions [41, 42]. Corrosion-related failures can cause short circuits, increase internal resistance, and promote the decomposition of electrodes and electrolytes. Electrochemical corrosion directly affects the performance of battery systems by reducing specific capacity and active material utilization due to continuous metal dissolution. The formation of unstable passive films and corrosion products increases interfacial resistance, thereby reducing the rate capacity and causing greater polarization. Unfavorable electrochemical corrosion directly affects key battery performance parameters. The dissolution of active metals at the anode reduces their capacity and lowers the energy density. The formation of passive films of oxides, hydroxides, or corrosion inhibitors increases interfacial charge transfer resistance, thereby reducing power output and increasing polarization. At the same time, excessive H_2_ evolution reactions utilize electrolyte components and electrons without contributing to applicable charge (energy) storage. This leads to a decrease in Coulombic efficiency and accelerates self-discharge. Uneven metal dissolution and localized corrosion accelerate the risks of dendrite growth in rechargeable batteries. Dendrite growth not only reduces reversibility but also increases the risk of rapid capacity fading and internal short circuits. Therefore, in battery systems, corrosion is not just a problem of material (electrode) degradation but a fundamental factor affecting battery efficiency, safety, cycle life, and rate capability. Corrosion-related shortcomings can pose environmental, health, and safety risks through interfacial corrosion and subsequent leakage.

Zhang et al. observed and reported that in Li-ion batteries, corrosion of Al (cathode) and Cu (anode) collectors results in contact loss, reduced power, short circuits, and increased resistance under stress [43]. High voltage and over-discharging cause Al and Cu to dissolve, respectively. During recharging, these processes pose safety risks, cause capacity loss, and lead to short circuits. Ruetschi proposed that corrosion is one of the main factors determining the batteries' lifespan [44]. The corrosion rate in battery electrolytes depends on temperature, voltage, and the metals (anodes and cathodes). The author proposed that the lifespan of batteries can be reduced by 50–75% if these factors are not adequately controlled or managed. Valve-regulated lead-acid batteries (VRLA) employed in UPS systems fail only in 3–5 years, much before their 10-year design life. Corrosion significantly reduces life expectancy. Interfacial interactions between electrodes and subsequent corrosion can reduce Li-ion battery performance by up to 20% [45, 46]. In EV lead-acid batteries, 30%-50% failures resulted from corrosion of the lead grids.

**Fig. 3:**
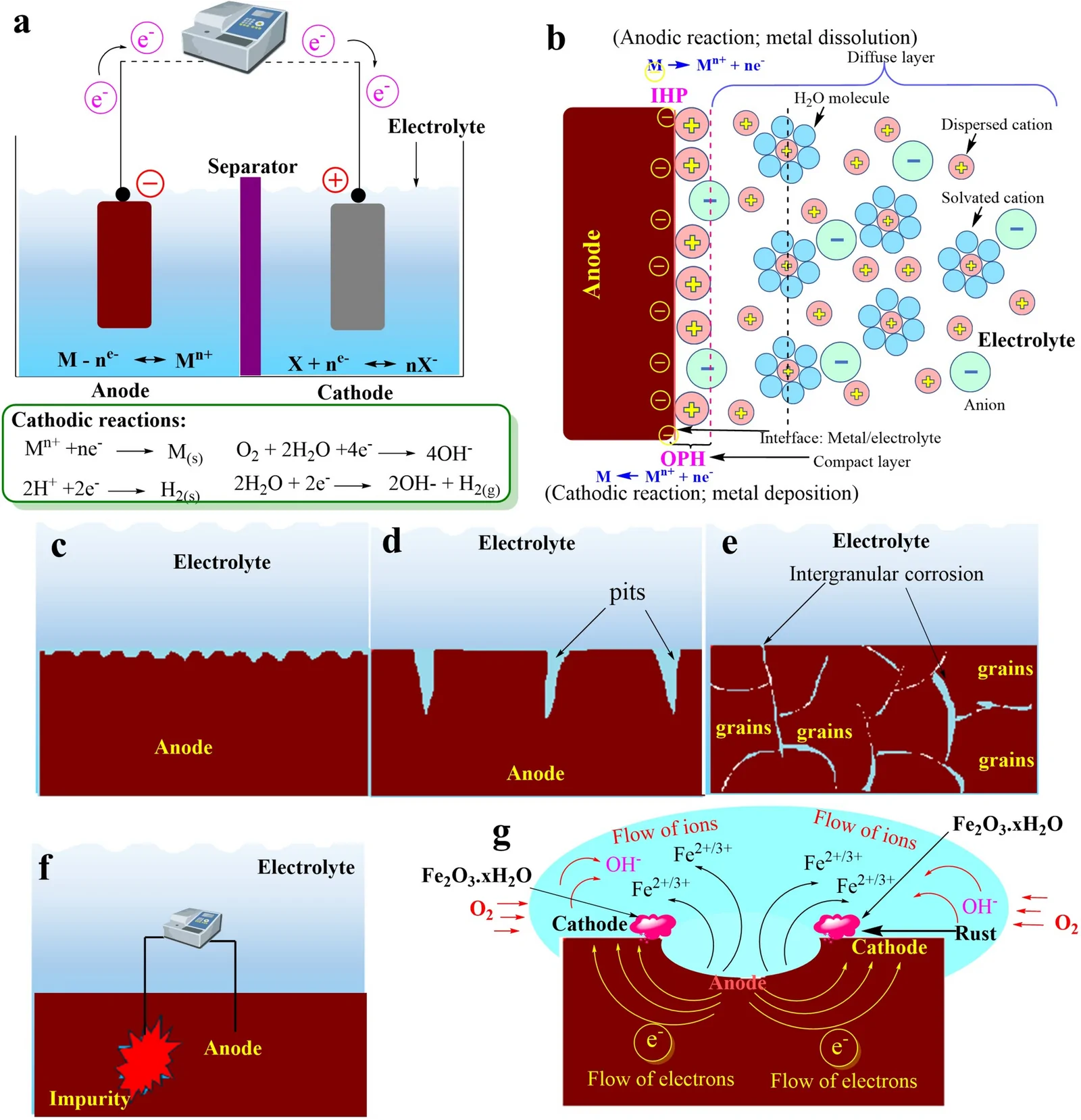
Schematics showing **a** mechanisms of anodic and cathodic reactions in aqueous electrolytes, **b** formation of the electric double layer/interface between metal and electrolyte, **c** uniform (general) corrosion, **d** pitting corrosion, **e** intergranular corrosion, **f** galvanic corrosion, and **g** the formation of aeration concentration cells due to oxygen gradient

In batteries, Al, Zn, and Mg are frequently used as anodes due to their compatibility and high energy densities; however, they face significant corrosion challenges, especially in aqueous electrolytes [47, 48]. In these systems, corrosion is mediated by redox reactions that lead to undesirable and irreversible loss of metallic material, as well as adverse effects on battery performance, including shortened lifespan and reduced capacity. Mechanistically, corrosion can be classified into two types: general (uniform) and localized [49]. General corrosion occurs uniformly throughout the anode surface, causing uniform surface thinning or loss. On the other hand, in localized corrosion, some specific areas of the electrode surface experience more severe corrosion than the adjacent areas. The diffusion of electrolyte components (e.g., Cl^−^ and H_2_O) and oxygen (O_2_) or other active oxidants into the defective regions results in the formation of a pitting nucleus. Most localized corrosion occurs through the formation of aeration concentration cells, i.e., O_2_-rich cathode and O_2_-deficient anode regions. Corrosion can also initiate and propagate along grain boundaries [50, 51]. Obviously, the diffusion of corrosive or active species will be relatively easy in grain boundaries due to the high surface defect density. This results in easy setting of electrochemical cells, mainly through the aeration concentration mechanism. After initiation, corrosion may propagate along grain boundaries (intergranular corrosion) or through grains (intragranular corrosion). The dissolution mechanism can involve galvanic corrosion if the anodes contain metallic impurities [52, 53]. The mechanisms of uniform (general) and localized corrosion are schematically presented in Fig. 3. Figure 3a illustrates the anodic metallic dissolution and cathodic reduction reactions occurring in the aqueous electrolytes. Notably, in anode dissolution, a single anodic reaction (oxidation) occurs, whereas several cathodic reactions are possible depending on the electrolyte composition. Hydrogen evolution and oxygen reduction are significant cathodic reactions in proton- and oxygen-based electrolytes, respectively. Figure 3b depicts the development of the electric double layer at the interface of the metal and electrolyte. This schematic illustrates the formation of the inner Helmholtz plane (IHP), the outer Helmholtz plane (OHP), and the diffuse layer (bulk solution), showing the interfacial potential distribution and charge separation. The general or uniform corrosion of the anode in the battery electrolyte is illustrated in Fig. 3c, which shows a homogeneous material loss throughout the surface. Localized corrosion mechanisms, including pitting, intergranular, and galvanic corrosion, are described in Fig. 3d–f. Lastly, the formation of oxygen gradient cells, or concentration cells, due to different oxygen concentrations at the anode and cathode regions, and the subsequent corrosion, is schematically presented in Fig. 3g.

The corrosion mechanisms of different anodes, including Mg, Zn, Al, and Fe, vary with the environment's pH [46, 54]. At acidic pH levels, these metals dissolve due to the reduction in hydrogen ion concentration. In neutral conditions, oxygen reduction is a significant cathodic reaction that forms oxide and hydroxide ions. Dissolved metal ions react with oxide and hydroxide ions, forming the corresponding metal oxides and hydroxides, which passivate the metal surface. The protective nature of the passive layer varies depending on the nature of the metals, pH, and other environmental factors. The corrosion mechanisms of some common anodes, including Zn, Mg, Al, and Fe, are presented herein. A more detailed account of the inhibition mechanisms can be found elsewhere [46]. Among the different anode materials used in sustainable batteries, Zn has attracted particular attention owing to its favorable electrochemical properties and compatibility with aqueous electrolytes used in battery applications. Nevertheless, the corrosion behavior of Zn strongly depends on pH and the dissolved species of the electrolyte.

(i) Corrosion Behavior of Zn-Anode

**Fig. 4:**
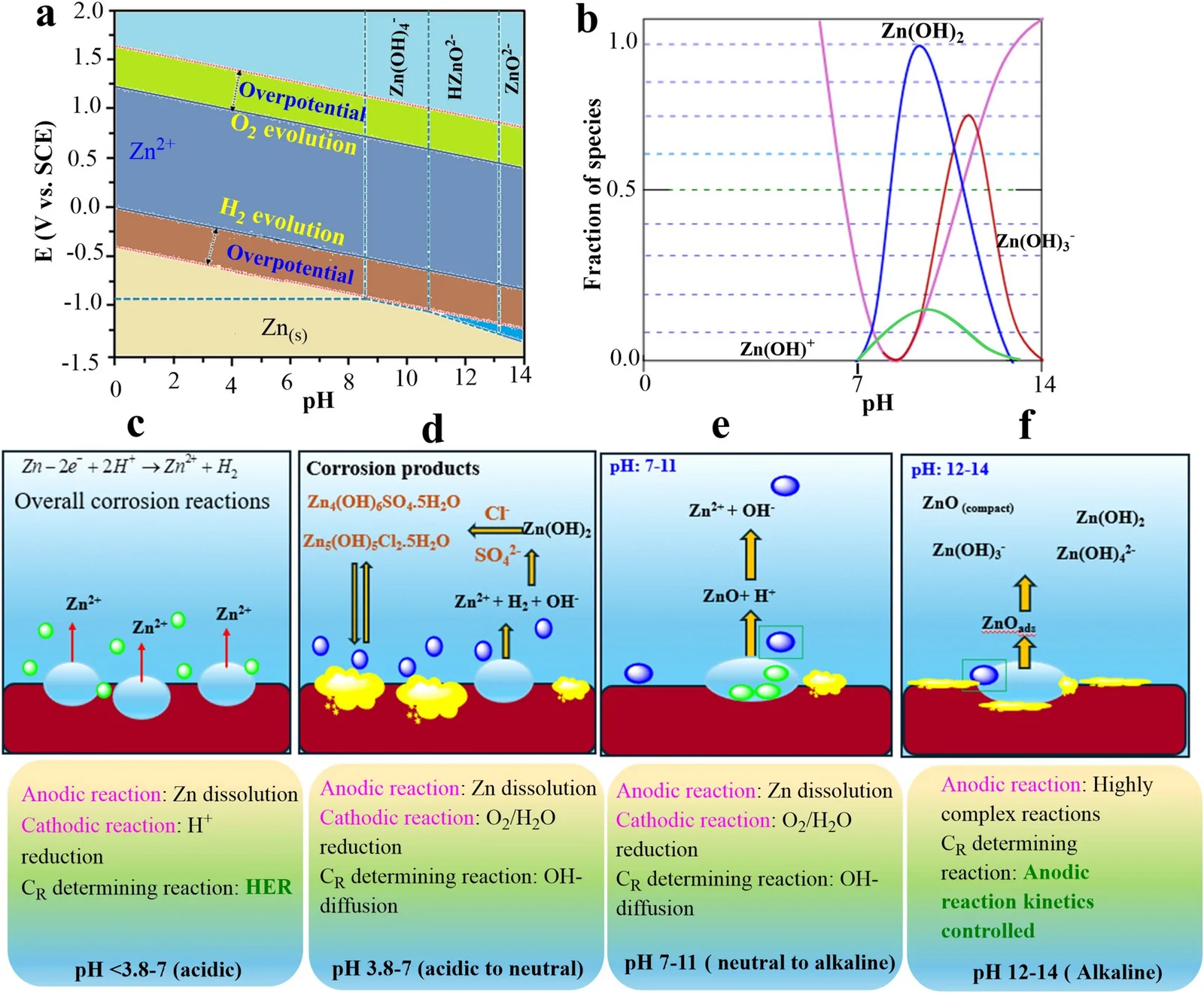
Schematic illustrations of **a** Pourbaix diagram of Zn-H_2_O system at RT, **b** relative fraction of different species at various pH, **c**–**g** the dissolution mechanisms of Zn, formation of different corrosion products at different pH, and protection mechanisms of corrosion products

Zn is a sacrificial anode in different systems as it readily oxidizes and forms Zn^2+^ ions. In acidic conditions, zinc dissolves by reducing H^+^ ions, releasing hydrogen gas (H_2_). In neutral environments, reduction of O_2_ occurs as a major cathodic (reduction) reaction, forming Zn(OH)_2_ that provides slight corrosion protection. Zn forms highly soluble Zn(OH)_4_^2−^ (zincate) ions in alkaline conditions. Zn(OH)_4_^2−^ ions accelerate the dissolution of the passive layer, thereby increasing the corrosion rate, particularly at elevated alkalinity. The behavior of Zn corrosion in different pH levels is as follows:

***Corrosion of Zn in acidic environments*** The nature of Zn corrosion greatly depends upon the electrolytes' pH, as seen by the Pourbaix diagram of the Zn-H_2_O system (Fig. 4a). Figure 4b shows that the formation of different corrosion products also depends on pH: Low pH favors the formation of Zn^2+^ ions, whereas high pH favors the formation of Zn(OH)_4_^2−^ ions. On the other hand, the formation of ZnO and Zn(OH)_2_ predominantly occurs at intermediate pH levels. The dissolution of Zn at the anode liberates electrons, which are consumed at the cathode by species, such as H^+^, H_2_O, and O_2_, leading to self-dissolution. In acidic conditions, the anodic dissolution, cathodic Zn deposition, and H_2_ evolution reactions occur as follows [46]:

1 $$ {\mathrm{Zn}} \to {\mathrm{Zn}}^{{^{2} + }} + 2{\mathrm{e}}^{ - } \;\;\;\;\;\left( {\text{anodic dissolution}} \right) $$

2 $$ {\mathrm{Zn}}^{{^{2} + }} + 2{\mathrm{e}}^{ - } \to {\mathrm{Zn}}_{{\mathrm{(solid)}}} \;\;\;\;\left( {\text{cathodic deposition}} \right) $$

3 $$ 2{\mathrm{H}}^{ + } + 2{\mathrm{e}}^{ - } \to {\mathrm{H}}_{{2({\mathrm{gas}})}} \;\;\;\;\left( {{\mathrm{H}}_{{2}} {\mathrm{evolution}}} \right) $$

In acidic conditions, Zn^2+^ ions form as the thermodynamically most stable species, and the corrosion rate is mainly governed by the hydrogen evolution reaction (HER) [55, 56]. However, at slightly higher acidic pH (3–5), the diffusion of H^+^ ions (to the cathode) becomes slower, and the rate of Zn corrosion is mainly governed by H^+^ ions diffusion or mixed kinetics [57, 58]. Figure 4c, d represents the mechanism of Zn corrosion in acidic environments.

***Corrosion of Zn in neutral environments*** In neutral or mildly acidic electrolytes, the corrosion rate (C_R_) determining process shifts from HER to oxygen reduction reaction (ORR). In these circumstances, the cathodic reactions can be illustrated as follows [46, 56]:

4 $$ {\mathrm{H}}_{{2}} {\mathrm{O}} + 2{\mathrm{e}}^{ - } \to {\mathrm{2OH}}^{ - } \;\;\;\;\;\;\;\left( {\text{deaerated environment}} \right) $$

5 $$ {\mathrm{O}}_{2} + 2{\mathrm{H}}_{{2}} {\mathrm{O}} + 4{\mathrm{e}}^{ - } \to 4{\mathrm{OH}}^{ - } \;\;\;\;\;\;\;\;\;\left( {\text{Aerated environment}} \right) $$

The ORR reaction kinetics are activation-controlled, and because of their very high irreversibility, they are independent of electrolyte pH. The presence of oxygen can significantly increase the C_R_. In these conditions, the anodic dissolution reactions are relatively complex and involve many ways, including Eq. (1). The anodic responses can be expressed as follows [46, 56]:

6 $$ {\mathrm{Zn}}^{2 + } + {\mathrm{2OH}}^{ - } \to {\mathrm{Zn(OH)}}_{2} $$

7 $$ {\mathrm{Zn(OH)}}_{{2}} \to {\mathrm{ZnO}} + {\mathrm{H}}_{{2}} {\mathrm{O}} $$

8 $$ {\mathrm{Zn(OH)}}_{2} + {\mathrm{2OH}}^{ - } \to {\mathrm{Zn(OH)}}_{{4}}^{{2 - }} $$

Notably, corrosion products are relatively porous, allowing the diffusion or penetration of corrosive species, such as Cl^−^ and SO_4_^2−^ ions. These ions may react with Zn^2+^ and other corrosion products to form corresponding Zn salts. For example, Cl^−^ ions can react with Zn^2+^ and Zn(OH)_2_ to form corresponding hydroxide compounds, as expressed by the following equations [46, 59]:

9 $$ 5{\mathrm{Zn}}^{2 + } + 8{\mathrm{OH}}^{ - } + 2{\mathrm{Cl}}^{ - } + {\mathrm{H}}_{{2}} {\mathrm{O}} \to {\mathrm{Zn}}_{5} ({\mathrm{OH}})_{8} {\mathrm{Cl}}_{2} \cdot {\mathrm{H}}_{{2}} {\mathrm{O}} $$

10 $$ 5{\mathrm{Zn(OH)}}_{2} + + 2{\mathrm{Cl}}^{ - } + {\mathrm{H}}_{{2}} {\mathrm{O}} \to {\mathrm{Zn}}_{5} ({\mathrm{OH}})_{8} {\mathrm{Cl}}_{2} \cdot {\mathrm{H}}_{{2}} {\text{O + 2OH}}^{ - } $$

Likewise, ClO_4_^−^ and SO_4_^2−^ ions can accelerate the Zn corrosion by following reactions [46, 59, 60]:

11 $$ {\text{Zn + 2ClO}}_{4}^{ - } \to {\mathrm{Zn(ClO}}_{4} )_{{\mathrm{2(aq)}}} + 2{\mathrm{e}}^{ - } $$

12 $$ {\mathrm{Zn(OH}})_{2} + 3{\mathrm{Zn}}^{2 + } + 4{\mathrm{OH}}^{ - } + {\mathrm{SO}}_{4}^{2 - } + 5{\mathrm{H}}_{{2}} {\mathrm{O}} \to {\mathrm{Zn}}_{{4}} {\mathrm{(OH)}}_{6} {\mathrm{SO}}_{4} \cdot 5{\mathrm{H}}_{{2}} {\mathrm{O}} $$

Zn_4_(OH)_6_SO_4_.5H_2_O forms a more compact and protective layer than Zn_4_(OH)_8_Cl.H_2_O, mainly due to the aggressiveness of Cl^−^ ions. The formation rate of ion pairs or complexes accelerates the Zn dissolution of these ions, following the following trend: Cl^−^ > SO_4_^2−^ > ClO_4_^−^. The pH of Zn-based batteries typically ranges between 3 and 6 [46, 61].

*Corrosion of Zn in alkaline environments* In alkaline environments, the reduction of H_2_O and O_2_ (ORR) contributes as a significant cathodic reaction. In the pH range of 7 to 10, zinc dissolution occurs via two mechanisms.

13 $$ {\text{Zn + H}}_{{2}} {\mathrm{O}} \to {\text{ZnO + 2H}}^{ + } + 2{\mathrm{e}}^{ - } $$

14 $$ {\text{Zn + H}}_{{2}} {\text{O + H}}^{ + } \to {\mathrm{Zn(OH)}}^{ + } + 2{\mathrm{H}}^{ + } + 2{\mathrm{e}}^{ - } $$

The passive film formed on the ZnO does not provide significant corrosion protection. This type of passive film is commonly called a pseudo-passive film. The formation of the pseudo-passive film is a net result of passive film destabilization by H^+^ ions and its formation as the corrosion reactions progress. Notably, oxides and hydroxides form at high pH (11–13), and the kinetics of anodic reactions mainly govern the *C*_R_. At pH 11, the insufficient supply of H^+^ results in limited Zn hydrolysis, leading to small domains of passivity. At pH 12, a compact film of ZnO can be formed by the separate or combined actions of the adsorption and nucleation-growth models, which can be illustrated as follows [62, 63]:

15 $$ {\text{Zn + OH}}^{ - } \to {\mathrm{ZnOH}}_{{{\mathrm{ads}}}} + {\mathrm{e}}^{ - } $$

16 $$ {\mathrm{ZnOH}}_{{{\mathrm{ads}}}} \to {\text{ZnO + H}}^{ + } + {\mathrm{e}}^{ - } $$

17 $$ {\text{Zn + 2OH}}^{ - } \to {\text{ZnO + H}}_{{2}} {\text{O + }}2{\mathrm{e}}^{ - } $$

The compact ZnO film thus formed is referred to as a type III oxide. A type III oxide film is relatively thin but provides significant protection against further corrosion. Zn(OH)_3_^−^ forms on further increasing the alkalinity (pH: 13–14), as expressed below [46, 56]:

18 $$ {\mathrm{ZnOH}}_{{{\mathrm{ads}}}} {\text{ + OH}}^{ - } \to {\mathrm{Zn(OH)}}_{2} + {\mathrm{e}}^{ - } $$

19 $$ {\mathrm{Zn(OH)}}_{{2}} {\text{ + OH}}^{ - } \to {\mathrm{Zn(OH)}}_{3}^{ - } $$

As corrosion reactions progress, the formation of Zn(OH)_3_^−^ is hindered by local supersaturation, which restricts the transport of OH- ions at the metal-electrolyte interface, leading to the gradual deposition of Zn(OH)_2_ on the surface. Simultaneously, the dissolution of Zn(OH)_3_^−^ and Zn(OH)_4_^2−^ also results in the building of ZnO, as per the following equations [46, 56]:

20 $$ {\mathrm{Zn(OH)}}_{{3}}^{ - } {\text{ + H}}^{ + } \to {\text{ZnO + 2H}}_{{2}} {\mathrm{O}} $$

21 $$ {\mathrm{Zn(OH)}}_{{4}}^{{2 - }} \to {\text{ZnO + H}}_{{2}} {\text{O + 2OH}}^{ - } $$

Thus formed, the ZnO passive film is referred to as a type I oxide. In this process, the dissolution of Zn(OH)_2_ is the rate-determining step. When the OH-concentration decreases, the dissolution of Zn(OH)_2_ halts, and a well-defined, highly protective oxide film, known as a type II oxide, forms. Noticeably, type I and II oxides form relatively larger proportions; however, type III oxide plays a more significant role in transiting the Zn surface in the passive (oxide) state [63]. Typically, alkaline Zn and Zn-air batteries use highly concentrated KOH solutions with a pH above 13.

Cathodic reactions can be considered rate-determining steps in the corrosion of Zn anodes in batteries. In milder acidic conditions (pH 4–6), H^+^ diffusion significantly affects the CR, whereas at pH 7–10, ORR becomes the main cathodic reaction. A higher pH favors the formation of corrosion-protective passive films. Numerous active species, including I_3_^−^, Br_2_, and Br_3_^−^, can diffuse to the Zn anode, leading to chemical corrosion. Oxygen and hydrogen evolution are commonly experienced in Zn-I_2_ cells, possibly due to interactions of iodine species with Zn(OH)_2_ [64]. Self-discharge and chemical corrosion are influenced by the diffusion of active Br_2_ and Br_3_^−^ in Zn-Br_2_ batteries. Localized corrosion, primarily pitting corrosion, is commonly encountered in Zn batteries due to the diffusion of halide ions through defective passive films. Sometimes, local acidification resulting from the hydrolysis of Zn and potential differences in pits and adjacent areas can form galvanic cells. Both localized processes increase the anodes’ *C*_R_. In practical Zn-based battery systems, these material degradation processes directly translate into reduced battery performance. The continuous, undesirable degradation and loss of Zn result in lower specific capacity and reduced active material utilization over repeated cycles. Moreover, H_2_ evolution due to cathodic reduction accelerates electrolyte and electron consumption, promoting self-discharge and reducing coulombic efficiency. Localized corrosion and uneven metal dissolution or deposition can lead to dendrite growth and a rougher surface, which, in turn, shortens cycle life and increases polarization. The accumulation of corrosion products and ZnO on the electrode surface can adversely affect ion transport pathways, reduce rate capability, and increase impedance. Given these, effective corrosion mitigation is essential to achieve long-term cycling stability, a stable discharge voltage, and high reversibility in Zn-based battery systems. The corrosion on Zn is primarily governed by zincate formation and H_2_ evolution in alkaline electrolytes. Al exhibits markedly different corrosion behavior due to its amphoteric nature and its ability to form highly stable, protective oxide films. Therefore, the mechanisms of Al corrosion have been analyzed and presented separately.

(ii) Corrosion Behavior of Al-Anode

Al-ion batteries, especially Al-air batteries, which use highly acidic or alkaline electrolytes, have recently shown great potential for battery applications. Being amphoteric, AL is highly sensitive to alkaline and acidic environments. The Pourbaix diagram of the Al-H_2_O system indicates that Al is highly susceptible to corrosion due to the decomposition of water, as the stability domain of Al is significantly lower compared to that of H_2_O. The dissolution of Al results in the formation of Al^3+^, Al_2_O_3_, and AlO_2_ depending on pH. Due to Al's high reactivity, a thin, compact oxide layer forms on the Al surface, which is highly soluble in acidic electrolytes. In acidic environments, the dissolution of the oxide layer involves a field-assisted or chemical process as expressed below [46, 65, 66]:

22 $$ {\mathrm{Al}}_{{2}} {\mathrm{O}}_{{3}} {\text{ + 6H}}^{ + } \to {\mathrm{2Al}}^{{3 + }} {\text{ + 3H}}_{{2}} {\mathrm{O}} $$

23 $$ {\mathrm{Al}}_{{\mathrm{(ox)}}} \to {\mathrm{2Al}}_{{{\mathrm{aq}}}}^{{3 + }} {\text{ + Al}}_{{\mathrm{Al(ox)}}}^{{3 - }} $$

In these equations, Al_(ox)_, Al_aq_^3+^, and Al_(ox)_^3−^ represent Al atoms in oxide film, Al^3+^ ions in aqueous solution, and Al vacancy in oxide film, respectively. The H^+^ ions present in the electrolyte can move to the oxide film and form H_2_O by reacting with O^2−^. Meanwhile, O^2−^ species can be eliminated, and Al^3+^ ions can enter the lattice to form Al_2_O_3_. The migration of Al^3+^ to Al vacancies, accompanied by the evolution of H_2_ at the Al/Al_2_O_3_ interface, results in Al surface pitting corrosion. The aggressive H^+^ ions freely attack the bare Al surface with a rapid increase in corrosion rate, once the surface oxide film is completely removed, as expressed as follows:

24 $$ {\text{Al + 3H}}^{ + } \to {\mathrm{Al}}^{{3 + }} {\text{ + 3/2H}}_{{2}} $$

Aggressive anions, including SO_4_^2−^, Cl^−^, and NO_3_^−^, complicate the corrosion process. Because of their great diffusibility and aggressiveness, the Cl^−^ ions produce more significant effects than other anions. Cl^−^ ions participate in Al corrosion and the oxide layer dissolution as follows [66]:

25 $$ {\text{Al + nCl}}^{ - } \to {\mathrm{AlCl}}^{{\text{(n - 3) - }}} + 3\mathrm{e}^{ - } $$

26 $$  {\mathrm{Al}}^{3 + } ({\mathrm{Al}}_{2} {\mathrm{O}}_{3} ) + 4{\mathrm{Cl}}^{ - } \to {\mathrm{AlCl}}_{4}^{ - }  $$

Cl^−^ ions preferentially adsorb on the Al surface by replacing the pre-adsorbed H_2_O molecules, favoring the transfer of Al from metal to solution phase. The mechanism of Al corrosion in acidic electrolytes containing SO_4_^2−^ and NO_3_^−^ ions is significantly slower, which may be attributed to their lesser aggressiveness compared to Cl^−^ ions. Some studies suggest that the presence of SO_4_^2−^ ions decreases the aggressiveness of Cl^−^ ions by preferential adsorption of less aggressive SO_4_^2−^ ions [67, 68]. Another opinion proposes that SO_4_^2−^ ions form surface protective Al_2_(SO_4_)_3_.18H_2_O and Al(OH)SO_4_^−^based passive films, which protect against corrosive dissolution [69]. NO_3_^−^ ions increase the stability of the passive film, decreasing the C_R_. Nevertheless, several species such as NO, N_2_, Al(NO_3_)_3_, NO_2_^−^, and NH_3_ form in the presence of NO_3_^−^ ions, making the dissolution mechanism complex, unclear, and unestablished [70–72]. In some studies, the authors claimed that these species can react with Al/Al^3+^ to form complexes that can accelerate the C_R_ [73]. These discussions revealed that the dissolution of Al in acidic environments depends more on the nature and behavior of anions than on the pH of electrolytes.

The oxide film is highly stable and insoluble, according to the mechanism of Al dissolution in neutral electrolytes. Coronation starts with the hydrolysis of the Al–O–Al bond of Al_2_O_3_, which forms the corresponding Al–OH species. As hydrolysis continues, the surface oxide film converts to a highly porous, poorly crystallized pseudo-boehmite (i.e., AlOOH) film, allowing water molecules to diffuse to the Al-AlOOH interface. Obviously, at this stage, C_R_ is controlled by hydrolysis. Corrosion begins immediately when H_2_O molecules meet the Al surface. The Al^3+^ ions so formed react with OH^−^ ions of water, as presented below [46, 74]:

27 $$ {\mathrm{Al}}^{{3 + }} {\text{ + OH}}^{ - } \to {\mathrm{Al(OH)}}^{{2 + }} $$

28 $$ {\mathrm{Al}}^{{3 + }} {\text{ + 2OH}}^{ - } \to {\mathrm{Al(OH)}}_{{2}}^{ + } $$

29 $$ {\mathrm{Al}}^{{3 + }} {\text{ + 3OH}}^{ - } \to {\mathrm{Al(OH)}}_{{3}} $$

The growth and nucleation of Al hydroxide govern the *C*_R_ at this stage. Al(OH)^2+^ and Al(OH)_2_^+^ species diffuse into the solution, and H_2_O molecules diffuse to the interface of Al/Al_2_O_3_. Over time, the oxide film thickness and its passivating behavior increase, reducing the *C*_R_. Therefore, the transport of these species determines the *C*_R_. The nucleation and growth of H_2_ gas bubbles at the Al/Al_2_O_3_ interface may adversely affect surface passivation, leading to pitting corrosion. In mild alkaline solutions, the corrosion mechanism is like that in neutral electrolytes, forming numerous species, including Al(OH)_3_, Al_2_O_3_, and AlOOH. OH- ions of water may diffuse to Al/Al_2_O_3_ and disrupt the oxide film stability as follows:

30 $$ {\mathrm{Al(OH)}}_{{3}} {\text{ + OH}}^{ - } \to {\mathrm{Al(OH)}}_{{4}}^{ - } $$

At this stage, diffusion of Al(OH)_4_^−^ and OH^−^ to or from the Al_2_O_3_/electrolyte interface can be considered the rate-determining step. In alkaline electrolytes, the Al^3+^ ions are thermodynamically unstable, which renders their direct ejection from the oxide films. Nevertheless, the dissolution of Al_2_O_3_ films proceeds as per the following equation:

31 $$ {\mathrm{Al(OH)}}_{{3}} {\text{ + OH}}^{ - } {\text{ + H}}_{{2}} {\mathrm{O}} \to {\mathrm{Al(OH)}}_{{4}}^{ - } $$

The oxide film is completely dissolved in fundamental (alkaline) solution, resulting in direct attack of the Al surface by OH^−^ ions, as expressed below:

32 $$ {\text{Al + 4OH}}^{ - } \to {\mathrm{Al(OH)}}_{{4}}^{ - } $$

Moreover, the diffusion of Al(OH)_4_^−^ and OH^−^ does not significantly influence the *C*_R_ in highly alkaline solutions. Moreover, the presence of alloying metal impurities leads to the formation of galvanic cells and subsequent galvanic corrosion in Al batteries. Mg demonstrates even greater dissolution complexity and chemical reactivity than Zn and Al. Mg-based systems are particularly challenging in aqueous batteries, as they are prone to substantial hydrogen evolution and are subject to multiple competing models.

(iii) Corrosion Behavior of Mg-Anode

The Mg anode exhibits higher self-corrosion than Zn and Al due to its high reactivity. Due to its high reactivity, Mg in intermetallic alloys such as Mg_2_Si and Al_3_Fe acts as the anode, with the remaining alloying elements serving as the cathodes. Mg-air and Mg-MnO_2_ are typical examples of Mg-based aqueous batteries. Rechargeable Mg-air batteries face significant challenges due to their poor high-rate stability and low energy conversion efficiency. The Pourbaix diagram indicates that Mg lacks a stable domain in aqueous electrolytes because its equilibrium potential is lower than that for H_2_ evolution. The behavior of Mg corrosion is more complicated than that of Zn and Al. Several hypothetical mechanisms, including the univalent film-based model, the Mg-hydride intermediate model, the Mg-based model, and the metal spalling model, have been proposed to explain the dissolution of the Mg anode in Mg-based aqueous batteries. These models have their own benefits and drawbacks. The Mg-hydride intermediate model suggests that on the Mg surface, MgH_2_ exists and plays a significant role in corrosive dissolution [75, 76]. Firstly, Mg is reduced to form MgH_2_, followed by its oxidation in neutral or alkaline solution to form Mg(OH)_2_ or Mg^2+^ ions in acidic solution, as expressed follows:

33 $$ {\text{Mg + 2H}}^{ + } + 2{\mathrm{e}}^{ - } \to {\mathrm{MgH}}_{{2}} $$

34 $$ {\mathrm{MgH}}_{{2}} {\text{ + 2H}}_{{2}} {\mathrm{O}} \to {\mathrm{Mg(OH)}}_{{2}} {\text{ + 2H}}_{{2}} $$

35 $$ {\mathrm{MgH}}_{{2}} {\text{ + 2H}}^{ + } \to {\mathrm{Mg}}^{{2 + }} {\text{ + 2H}}_{{2}} $$

The unprotected Mg can also be dissolved directly to form Mg^2+^ ions, as shown below [77]:

36 $$ {\mathrm{Mg}} \to {\mathrm{Mg}}^{2 + } + 2{\mathrm{e}}^{ - } $$

The univalent model proposed that Mg dissolution initiates with the formation of univalent Mg (Mg^+^), as expressed below:

37 $$ {\mathrm{Mg}} \to {\mathrm{Mg}}^{ + } + {\mathrm{e}}^{ - } $$

The univalent Mg^+^ ions are volatile and readily oxidized to Mg^2+^ as per the following mechanisms:

38 $$ 2{\mathrm{Mg}}^{ + } {\text{ + 2H}}^{ + } \to 2{\mathrm{Mg}}^{{2 + }} {\text{ + 2H}}_{{2}} $$

39 $$ 2{\mathrm{Mg}}^{ + } {\text{ + 2H}}_{{2}} {\mathrm{O}} \to 2{\mathrm{Mg}}^{{2 + }} {\text{ + 2OH}}^{ - } {\text{ + H}}_{{2}} $$

These dissolution processes have been extensively studied in acidic electrolytes, but the mechanisms of dissolution in neutral and alkaline electrolytes remain unclear. The film-based model proposes that, due to Mg's high reactivity, it readily forms Mg(OH)_2_ and MgO passive films, even in cathodic polarization zones. The passive films break once the anodic polarization potential exceeds the breakdown potential, leading to Mg dissolution [46, 78]. The uneven dissolution was attributed to a spalling mechanism, in which Mg dissolves as flakes or small particles, leading to H_2_ evolution without current flow [46, 79]. The recent findings suggest that insoluble Mg and Mg^2+^ ions do not participate in the dissolution process. Another model suggests that in the presence of aggressive ions, Mg dissolution starts by breaking down the MgO film at reactive sites. This ultimately results in the formation of Mg(OH)_2_, increasing Mg corrosion and H_2_ evolution. This process occurs at the MgO/Mg(OH)_2_ interface, where Mg^2+^ ions can diffuse in the electrolyte or precipitate on the surface as Mg(OH)_2_ [80, 81]. In neutral electrolytes, the overall corrosion reactions can be presented as follows:

40 $$ {\text{Mg + 2H}}_{{2}} {\mathrm{O}} \to {\text{MgO + 2OH}}^{ - } {\text{ + H}}_{{2}} $$

Mg-air and Mg-MnO_2_ batteries primarily use NaCl-based aqueous electrolytes, and the corrosion mechanism of the Mg anode can be described by the equation above. At high pH (above 10.5), the Mg(OH)_2_ film provides anticorrosion protection through passivation. The MgO and Mg(OH)_2_ films are volatile and soluble in acidic electrolytes, accelerating the C_R_, as follows [82, 83]:

41 $$ {\text{MgO + 2H}}^{ + } \to {\mathrm{Mg}}^{{2 + }} {\text{ + H}}_{{2}} {\mathrm{O}} $$

42 $$ {\text{Mg + 2H}}^{ + } \to {\mathrm{Mg}}^{{2 + }} {\text{ + H}}_{{2}} $$

In Mg-based alloys, Mg dissolution follows trends like those of pure Mg, with H_2_ evolution as the dominant cathodic reaction. The presence of alloying metal impurities leads to the setting of galvanic cells and subsequent galvanic corrosion in Mg batteries. The dissolution mechanism of the Fe anode and other metal anodes can be found elsewhere [45, 46, 84]. Although Zn, Mg, and Al anodes exhibit different electrochemical behavior, their corrosion mechanisms share a common fundamental: anodic metallic dissolution (actual electrode loss) and cathodic hydrogen evolution reaction (HER) or oxygen reduction reaction (ORR). In these systems, corrosion rates and mechanisms are strongly influenced by pH-dependent passivation and the stability of passive (oxide or hydroxide) films. Nevertheless, the significant differences originate from the properties of oxide layers and their intrinsic thermodynamic stability. Obviously, Zn forms amphoteric corrosion products, such as zincate, in alkaline electrolytes and exhibits relatively moderate self-corrosion. In acidic and neutral environments, corrosion is mainly controlled by the reduction of protons to form hydrogen gas and by the decrease in oxygen. In contrast, Al forms a highly stable, compact, and uniform oxide film under neutral conditions, resulting in relatively superior passivation. However, due to the amphoteric nature of Al-based passive films, they are susceptible to strong acidic or alkaline conditions and become highly unstable under such conditions.

Mg acquires a more negative equilibrium potential and is electrochemically more reactive. Mg lacks a wide stability domain in aqueous electrolytes and undergoes pronounced self-corrosion, leading to extensive H_2_ evolution. The uneven accumulation of corrosion products results in a rough Zn-based surface, leading to halide-induced dendrite growth and localized pitting corrosion. Likewise, chloride ingress also breaks the passive layers on the Al surface, while in Mg, galvanic coupling with intermetallic phases and passive film instability govern the passive film stability. These discussions show that, while corrosion mechanisms across different electrolyte systems and electrodes can be described by a unified mechanism, their severity, major degradation pathways, and passivation stability differ remarkably, necessitating material-specific corrosion protection strategies. The development, breaking, and regeneration of oxide- or hydroxide-based passive films play a significant role in controlling the kinetics of anodic dissolution reactions. The stability of these oxide or hydroxide films largely depends on transport phenomena, ionic species, pH, and applied polarization potential. Moreover, in Zn, Al, and Mg, localized (pitting or intergranular) corrosion occurs by the ingress or diffusion of corrosive species through surface defects, galvanic coupling among microstructural heterogeneities, and concentration gradients. As a result, anode corrosion in battery systems can be described as the coupling of different phenomena, including redox (electrochemical) reactions, transport processes, and passivation, which are governed by thermodynamic stability, mass transport, and corrosion kinetics.

In battery systems, in addition to intrinsic material and electrolyte properties, the degradation mechanisms and corrosion kinetics of anodes are greatly governed by operating conditions. By increasing the diffusion coefficients and charge transfer kinetics, temperature significantly increases corrosion and dissolution rates [85, 86]. Elevated temperatures adversely affect the stability of protective films, promoting the risks of localized corrosion and hydrogen evolution [87]. Similarly, fast charging rates and relatively high current density promote dendritic growth, leading to localized pitting, anodic polarization, and increased overpotential [88, 89]. High depth-of-discharge and repeated cycling conditions can induce mechanical stress on passive films, increasing the risk of cracking and exposing a fresh anodic surface to corrosive electrolyte [88, 89]. In seawater and flow-based battery systems, the hydrodynamic and electrolyte velocity conditions affect the mass transport of corrosive species (e.g., SO_4_^2−^, Cl^−^), thereby influencing concentration cell formation and localized corrosion [90, 91]. Moreover, pressure and state-of-charge fluctuations and oxygen concentration gradients may promote galvanic coupling and adversely affect interfacial stability [92, 93]. Thus, in practical battery systems, corrosion behavior not only depends on the nature and properties of the anodes and the electrolyte composition but is also coupled with real operating parameters.

Under extreme conditions, e.g., elevated temperature and high voltage, metal-ion corrosion in aqueous electrolytes is significantly intensified, significantly limiting the practical operating window. The thermodynamic driving force for anodic dissolution increases at relatively high anodic potential (*E*_anodic_), leading not only to accelerated oxidation and metal dissolution but also to destabilization of protective passive films [94]. Likewise, increased voltage can trigger electrolyte oxygen evolution reaction (OER), electrolyte decomposition, and degradative side reactions [95, 96]. These side reactions also generate several active species, which diminish the protective properties of oxide or hydroxide passive films. In Al-, Mg-, and Zn-based battery systems, the stability domains defined by the Pourbaix diagram may shift into instability domains due to local potential shifts at high polarization, accelerating the risks of pitting corrosion or active metal loss [97]. At the same time, an increase in temperature enhances charge transfer kinetics at the metal-electrolyte interface, ionic mobility, and the diffusion coefficient, thereby increasing HER and *i*_corr_ [98, 99]. An increase in temperature is also consistent with the high solubility of corrosion products, diminishing the integrity of oxide or hydroxide passive films and intensifying the risk of localized corrosion due to faster mass transport of aggressive anions such as Cl^−^ or SO_4_^2−^. Therefore, extreme conditions (elevated temperature and high voltage) can bring corrosion-mediated adverse effects, including capacity fading, dendrite formation and growth, impedance growth, and gas accumulation.

In practical battery systems, corrosion does not occur under static electrolyte conditions; instead, it appears in dynamically evolving chemical environments [100, 101]. During the cycling process, due to concentration polarization, oxygen reduction, metal-ion hydrolysis (solvation), and hydrogen evolution, the local pH can fluctuate. At the same time, several anions, including NO_3_^−^, Cl^−^, SO_4_^2−^, and other active species, may coexist with varying concentrations. Under such complex-electrolyte conditions, corrosion mechanisms compete and may transform in response to kinetic accessibility and thermodynamic stability [102, 103]. Thermodynamically, using the Pourbaix diagram, the dominant corrosion mechanism can be rationalized, providing solid insights into the domains of metal, oxide, and soluble species as a function of potential and pH [104]. Nevertheless, in practical systems, corrosion pathways are often controlled by kinetic parameters such as oxygen transport, diffusion of OH^−^ or H^+^, kinetics of passive film dissolution, adsorption strength of aggressive anions available in the electrolyte, and charge transfer resistance at the interface [105, 106]. For example, in zinc-based systems, corrosion or dissolution is mainly controlled by hydrogen evolution at low pH, whereas at neutral pH, dissolution is primarily controlled by the oxygen reduction reaction [107]. The presence of Cl^−^ at high concentrations induces a kinetically favored localized pitting corrosion, driven by autocatalytic local acidification and the breakdown of protective (passive) films. Conversely, sulfate ions may accelerate the formation of protective sulfate-containing surface films, shifting the corrosion mechanism toward uniform behavior. A similar competition can also be observed in Mg- and Al-based battery systems, where aggressive anion adsorption, oxide stability, and hydrolysis (solvation) reactions collectively determine whether uniform or localized dissolution predominates or passivation occurs. Therefore, in sustainable batteries, corrosion mechanisms depend on thermodynamic and kinetic factors.

The qualitative monitoring of the corrosion behavior of anode materials is of great practical importance, in addition to mechanistic knowledge and the factors that affect it, particularly in extensively employed Li-ion battery (LIB) systems [108, 109]. In LIB systems, corrosion mainly affects Cu and Al current collectors under elevated temperatures, over-discharge, and high-voltage conditions [110]. Therefore, electrochemical techniques such as electrochemical impedance spectroscope (EIS), linear polarization resistance (LPR), and potentiodynamic polarization (PDP) have been widely used to evaluate functional qualitative parameters such as R_ct_ (charge transfer resistance), R_p_ (polarization resistance), E_corr_ (corrosion potential), and i_corr_ (corrosion current density), to provide qualitative insight about passive films stability and kinetics of corrosion reactions [111–113]. Moreover, weight -loss and hydrogen evolution analyses are also employed to determine corrosion rates. The literature investigation also shows that several advanced surface characterization techniques, including SEM (scanning electron microscope), EDS (energy-dispersive X-ray spectroscopy), XPS (X-ray photoelectron spectroscopy), and ICP-MS (inductively coupled plasma mass spectrometry), have been used to detect trace metal dissolution and surface degradation [111]. Nowadays, in situ and operando diagnostic approaches have also emerged as a potential tool for studying interfacial material degradation via electrochemical mechanisms in realistic environments [114–116].

Corrosion behavior of electrodes in battery systems has been extensively studied using electrochemical techniques. In most of these investigations, electrochemical inhibition efficiencies are derived under simplified laboratory conditions, especially for short-duration experiments. Several electrochemical indices, such as *R*_p_, *R*_ct_, and *i*_corr_, while providing valuable insights into mechanisms, do not fully capture long-term dissolution under practical battery cycling conditions, which are generally characterized by temperature fluctuations, mechanical stress, repeated charge–discharge cycles, and changing electrolyte composition. Moreover, testing protocols, such as applied current density, exposure times, electrolyte composition (concentration), and the units used for reporting, vary widely across studies. The inconsistencies in these protocols and outcomes make direct comparison difficult, leading to inconsistent conclusions and challenging the assessment of the best-performing materials. The founding of unified benchmarking standards is highly vital for the next-generation sustainable battery systems to establish practice performance thresholds including: (i) sustained Coulombic efficiency of > 99%; (ii) capacity retention of > 80%-90% after extended cycling (e.g., over 1000–5000 cycles varying upon system); (iii) controlled H_2_ evolution rates; and (iv) stable impedance growth below defined limits over prolonged operations. To accelerate technology translation and enhance compatibility, long-term corrosion testing protocols are essential, especially in realistic operating battery conditions. The integrated testing frameworks will enable reproducibility, benchmarking, and the industrial implementation of corrosion mitigation approaches in sustainable battery systems.

The pH of electrolytes significantly impacts corrosion pathways, corrosion behavior, and the stability of passive films. Several anions, such as Cl^−^, Br^−^, and SO_4_^2−^ ions, enhance C_R_, whereas certain anions, especially NO_3_^−^ and CrO_4_^2−^, passivate the metal surface and decrease C_R_ [117–119]. Generally, an increase in temperature results in a rise in *C*_R_, and similar trends are observed with increasing current density (*i*_corr_). Properties of anodic metals, such as particle size, grain properties, distribution, and surface area, also affect the corrosion resistance behavior of anodes [46, 120]. The presence of elemental impurities may accelerate or decrease *C*_R_. The presence of contaminants, especially anions, in electrolytes may positively or negatively affect the *C*_R_ of anodic materials. In different battery systems, the degree of electrode corrosion is closely associated with the performance decay trends observed during cycling. The corrosion current density (*i*_corr_) derived through potentiodynamic polarization can be correlated with the capacity retention and self-discharge rates. Generally, an increased *i*_corr_ value is associated with reduced capacity retention and an increased self-discharge rate. Likewise, the formation of corrosion products and the development of passive films are associated with increased impedance values in EIS studies and larger voltage hysteresis during charge–discharge processes. These points indicate that monitoring *i*_corr_, *R*_ct_, *R*_p_, *R*_f_, *E*_corr_, and other parameters provides indirect but reliable indicators of battery porosity, health, and performance. In battery systems, the corrosion behavior of electrodes is strongly influenced by several interrelated physicochemical parameters, including temperature, pH, electrolyte composition, the nature of aggressive ions, and the applied potential (*E*_applied_). Each of these parameters can alter corrosion mechanisms, H_2_ evolution kinetics, mass transport properties, and the stability of passive films. Table 2 summarizes the physicochemical parameters that induce primary corrosion.

**Table 2:** A summary of physicochemical parameters mediated corrosion phenomena and their mechanistic aspects

S/N	Physicochemical parameter	Corrosion phenomenon	Mechanism(s)
1	Low pH (acidic condition)	Uniform corrosion; enhanced HER	Destabilization of passive films; accelerated H^+^ reduction
2	Neutral pH	ORR as a major cathodic reaction; porous oxide films	Diffusing controlled process; oxides or hydroxides formation
3	High pH (alkaline condition)	Zn, Al, and Mg form zincate, amphoteric Al_2_O_3_, and Mg(OH)_2_, respectively	Passive film dissolution; anodic activation at very high pH
4	Aggressive ions (e.g., Cl-)	Localized attacks, pitting corrosion	Local acidization and breakdown of passive (oxide) films
5	Relatively less aggressive ions (e.g., SO_4_^2−^ and NO_3_^−^)	Reduced localized pitting; improved passivation	Form relatively compact sulfate/nitrate-based films
6	High voltage	Localized corrosion, OER activation, and accelerated breaking of passive films	Electrolyte decomposition and shift stability domains in the Pourbaix diagram
7	Elevated temperature	Passive films weakening; increased H_2_ evolution; increases icorr and	Enhanced charge transfer kinetics; enhanced diffusion
8	Presence of oxygen	Aeration (concentration) cells; enhanced ORR-mediated corrosion	Concentration gradient development; cathodic activation
9	Accelerated current density	Localized corrosion; Dendrite growth	Polarization-induced instability; nonuniform ion flux

### Corrosion Could Be a Barrier and Stealing Problems in Sustainable Batteries

**Fig. 5:**
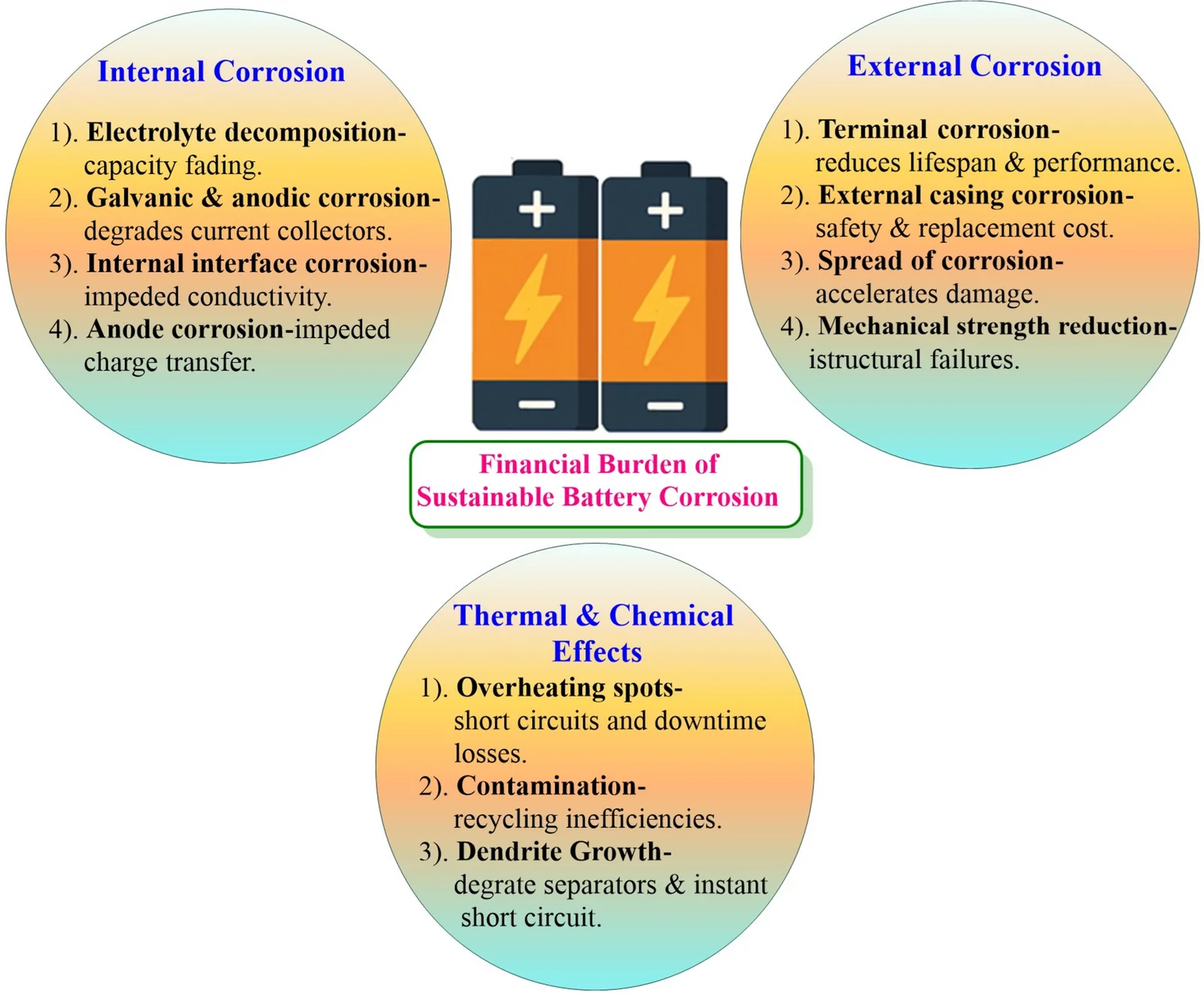
Schematic diagram showing corrosion-related multifaceted barrier to the future of the battery industry, particularly for aqueous electrolyte-based batteries

Corrosion poses a multifaceted barrier to the future of the battery industry, particularly for aqueous electrolyte-based batteries (Fig. 5) [49]. Corrosion adversely affects both the external casing and the internal electrochemical interfaces of batteries, reducing their lifespan, safety, and performance. Corrosion impedes the electrical conductivity between the battery and the device, leading to several severe issues, such as power loss or slow charging [121]. This results in sulfation and undercharging overtime. Once corrosion is initiated, it can spread to other components of the battery, causing further corrosion and damage. The corrosion of the battery terminals can also adversely affect the lifespan and performance. Overheating spots developed by corrosion may lead to parasitic shorts or drains. Qin et al. observed that corrosion from hydrothermal salt spray leads to a 76% decrease in the elastic modulus and induces swelling due to NaCl infiltration, resulting not only in reduced mechanical strength but also compromised integrity and safety risks, including thermal runaways and short circuits [122]. The challenges are particularly encountered in environments of high humidity. Corrosion-mediated failures affect the energy storage systems in electric marine vehicles (EMVs), coastal grids, and offshore installations. Du and coworkers demonstrated that galvanic and anodic corrosion, as well as electrolyte decomposition, degrade current collectors and electrodes, leading to capacity fading, gas evolution, and increased impedance [123]. Cu and Al collectors in Li-ion batteries experience galvanic and pitting corrosion, especially in humid environments, at elevated temperatures, and under elevated voltage.

In aqueous electrolyte-based batteries, Zn, Fe, Al, and Mg anodes readily corrode, evolve H_2_ gas, and form oxide (passive) films in the presence of O_2_ and H_2_O, thereby impeding charge transfer. Recent reports indicate that safe, sustainable, and recyclable battery systems rely heavily on the recovery of metals such as Co, Mn, Ni, Al, Mg, and Li [124–126]. However, corrosion degradation and the mixing of corrosion products, mainly oxides and hydroxides, reduce the purity of recovered metals and their recovery efficiency. Moreover, corrosion-mediated contamination can adversely affect the recycling process. The corroded and damaged casing, as well as electrode residue, may increase safety risks, especially in aged batteries, which require strict design and recycling standards.

## Electrochemical Corrosion Problems in AESs: Mechanisms, Consequences, and Mitigation

To thoroughly understand the corrosion mechanisms in battery systems, it is crucial first to examine the basic electrochemical reactions governing metallic dissolution and passivation in aqueous environments. Although the mechanism of corrosion varies depending on the nature of the metal and the electrolyte, it is generally governed by interfacial redox (electrochemical) reactions, the formation of passive (oxide) films, and mass transport processes. In this section, the corrosion behavior and mechanisms of representative anode materials, such as Zn, Mg, and Al, are discussed to highlight similarities and differences. In addition to gaining insights into corrosion mechanisms in battery systems, developing effective corrosion protection strategies is vital for enhancing the electrochemical stability and service life of next-generation sustainable batteries. In these systems, the corrosion protection strategies can be classified into three broad categories: (i) strategies related to intrinsic material designs that include microstructural optimization and alloying; (ii) strategies related to surface engineering, such as passivation, coatings, and artificial interphases [127]; and (iii) strategies related to electrolyte modification through the incorporation of solvation-controlling species, corrosion inhibitors, and functional additives. The objectives of these strategies include suppressing anodic electrode dissolution, cathodic H_2_ evolution, stabilizing passive films, reducing dendrite growth, and homogenizing metal deposition. These protection strategies, along with representative mechanisms, are systematically discussed in the following sections. In battery systems, corrosion protection strategies depend heavily on the form of corrosion.

The use of gel electrolytes has emerged as a practical approach to prevent interfacial corrosion in aqueous metal-ion batteries, particularly in Al-, Mg-, and Zn-based systems [128–131]. Unlike conventional electrolytes, gel-based systems are composed of confined water molecules within polymer networks, thereby reducing undesirable HER and water activities [128, 129, 132]. Moreover, the polymer matrix also restricts the ingress of aggressive anions, including sulfate and chloride, present in the electrolyte, thereby minimizing the risks of concentration-gradient-mediated galvanic effects and localized pitting corrosion [133–135]. Additionally, these electrolytes can accelerate the formation of relatively more uniform and stable solid-electrolyte interphases, which can serve as protective films, thereby reducing current density and improving the kinetics of interfacial charge transfer [136, 137]. Being mechanically robust, gel electrolytes also suppress dendrite propagation by regulating ion flux at the electrode surface. Therefore, gel-based electrolyte systems offer several benefits for batteries, including lower HER, reduced metal dissolution, improved Coulombic efficiency, enhanced safety, and long-term cycling stability [138, 139]. Owing to these properties, i.e., interfacial protection and electrolyte stabilization, the use of gel-based electrolytes in next-generation aqueous sustainable batteries is gaining particular attention.

In addition to gel and specific additive electrolyte systems, the balanced design of corrosion-resistant electrolytes also requires systematic consideration of physicochemical parameters under both alkaline and acidic conditions. Buffering capacity and pH control are highly critical parameters, as the corrosion rate depends strongly on hydroxide concentration and proton availability [140]. Obviously, the stable pH domains reduce the possibilities of passive film breakdown and retards excessive H_2_ evolution by reducing HER [141, 142]. Solvation structure and water activity are other important parameters that play a central role in the design of corrosion-resistant electrolytes [143]. Generally, free water molecules promote anodic dissolution and HER. The reactivity of water and its adverse consequences can be minimized using water-in-salt systems, high-concentration electrolytes, the incorporation of an external additive, or gel electrolytes [144–146]. These species form intermolecular hydrogen bonds with free water molecules, thereby reducing their activity and consequences. The selection of anions and their control of aggressiveness could be other practical approaches to designing corrosion-resistant electrolytes. For example, Cl^−^ ions promote localized corrosion by dissolving or diffusing through the surface oxide films, leading to pitting corrosion, whereas NO_3_^−^ or SO_4_^2−^ ions promote the formation of protective passive films [147, 148]. To avoid excessive side reactions and undesirable corrosion, the electrochemical stability window (ESW) and redox compatibility with electrode materials must be appropriately ensured. Excessive dendrite growth and polarization can be avoided by optimizing transport homogeneity and ionic conductivity [149, 150]. Moreover, SEI-forming ability and interfacial compatibility are crucial for stabilizing artificial interphases or oxides during cycling. Screening these parameters can provide a systematic framework for designing and developing corrosion-resistant electrolytes tailored to alkaline or acidic metal-ion batteries.

Table 3 summarizes the significant forms of electrochemical corrosion in aqueous electrolyte-based battery systems, along with their corresponding mechanisms, affected components, significant consequences, and primary prevention strategies. The corrosion in these systems manifests in several ways, including uniform, pitting, galvanic, and intergranular corrosion, as well as hydrogen- and dendrite-mediated corrosion and passive film instability. The different corrosion forms share the common anodic dissolution and cathodic HER or ORR reactions, but they differ in severity, effect on electrochemical performance, and localized behavior. The consequences range from gradual, slow material and capacity loss to increased impedance, polarization, and the risk of short circuits. Effective corrosion mitigation strategies include alloy design, microstructure control, grain refinement, corrosion inhibitors, surface engineering, electrolyte regulation, and interfacial stabilization.

**Table 3:** A summary of significant forms of electrochemical corrosion, mechanisms, affected components, significant consequences, and primary prevention strategies in aqueous electrolyte-based battery systems

S/N	Form of corrosion	Major degradation mechanism(s)	Major consequences	Components undergo degradation	Main mitigation strategies
1	General (uniform) corrosion	Uniform anode dissolution through ORR or HER	Gradual loss of electrodes, increased icorr, and reduced specific capacity	Anodes (Mg, Al, and Zn) and current collectors	Alloying, protective coatings, electrolyte modification, and inhibitors
2	Pitting	Breakdown of passive film, halide-induced degradation	Risks of short-circuits, rapid localized attacks, and dendrite growth	Al and Zn anodes in chloride-based electrolytes	Surface coatings, anion regulation (e.g., sulfate can be preferred over Cl^−^), and inhibitors
3	Intergranular corrosion	Preferential corrosion along with grain boundaries	Performance loss, mechanical strength loss, and crack development	Polycrystalline Mg, Al, and Zn anodes	Alloy design, passivation stabilization, and grain refinement
4	Galvanic corrosion	Intermetallic phases or dissimilar metal coupling	Localized corrosion and reduced cycle life	Current collectors (Cu/Al) and alloyed anodes	Impurity removal, surface homogenization, and control of microstructure
5	Instability of passive films	pH-dependent dissolution of passive films	Capacity fading due to increased polarization and impedance	Dissolve oxides and hydroxides, i.e., ZnO, Al_2_O_3_, and Mg(OH)_2_ films	Electrolyte buffering, pH control, and solvation control
6	H_2_ evolution catalyzed corrosion	HER-mediated anodic corrosion	Reduced Coulombic efficiency, self-discharge, and gas accumulation	Mg and Zn in H_2_O-based electrolytes	Reduction of H_2_O activity, cathodic protection, and electrolyte control
7	Dendrite-mediated corrosion	Localized corrosion due to uneven deposition	Capacity fading and risks of short circuits	Rechargeable Mg and Zn anodes	Surface modification and tip-blocking

### Electrochemical Corrosion and Corrosion Inhibition of Sustainable Zinc-based Batteries

Zn-based anodic materials have long been recognized for primary (non-rechargeable) as well as secondary (rechargeable) batteries owing to their cost-effectivity, high theoretical capacity (820 Ah kg^−1^), and electrochemical reversibility [151–154]. Nevertheless, their practical applications are significantly hindered by their susceptibility to corrosion in alkaline electrolytes, particularly for Zn-MnO_2_ and Zn-air batteries [155, 156]. Mercury amalgamation was widely employed in conventional batteries to prevent or minimize undesirable anodic corrosion (dissolution) by passivating the anode surface [84]. The growing interest in sustainable development in the early 1990s, following the establishment of green chemistry principles, led to efforts to mitigate battery corrosion in the search for Mercury-Free strategies. These strategies include alloy-based modifications and the use of external additives, commonly referred to as corrosion inhibitors. Organic compounds, primarily heterocycles, featuring various heteroatoms and polar functional groups, serve as effective corrosion inhibitors for anodic dissolution [157, 158]. These electron-rich sites serve as adsorption centers during their coordination with the anode (Zn) surface. For example, Nartey et al. screened the inhibition performance of a series of organic inhibitors for the dissolution of Zn in KOH solution [159]. The outcomes of different analyses showed that they strongly bind to the metallic surface and form protective hydrophobic films via π-orbital or coordination bonds. The presence of corrosion inhibitors significantly improves the charge–discharge stability and minimizes the H_2_ evolution. These effects typically improve capacity retention, Coulombic efficiency, cycle life, and reduce polarization under practical operating conditions. A year later, Kannan and coworkers adopted an alloying approach, integrating trace amounts of Mg, Pb, and Al into high-purity Zn to improve battery performance and corrosion resistance [160]. The alloying elements not only improve battery performance but also replace traditional inorganic additives, such as CaO, sodium citrate, and sodium stannate. The authors observed that cathodic H_2_ evolution and anodic dissolution were significantly retarded by alloying, attaining anodic inhibition efficiency of up to 99%. The literature study reveals that in the early 2000s, the use of protective thin films and polymeric surfactants began to protect against anodic dissolution [161]. They synergistically improve the surface morphology, retard H_2_ evolution, and anodic dissolution.

Zhu et al. proposed a Nb-based surface modification approach in which Pb(NO_3_)_3_ produces a protective Nb-rich surface oxide (passive) film on the Zn surface for adequate protection [162]. The Nb-rich surface oxide films provide uniform corrosion protection, minimize corrosion rate (*C*_R_), reduce current density (*i*_corr_), and increase charge transfer resistance (R_ct_). Significant progress in the use of organic and surfactant-type corrosion inhibitors to protect Zn anode dissolution was made between 2009 and 2015. These species, such as amidopoly ethylamines, develop a corrosion-protective hydrophobic layer, thereby inhibiting both anodic and cathodic reactions [163]. Mainly, they serve as mixed-type corrosion inhibitors, meaning that they retard both anodic Zn dissolution and cathodic H_2_ evolution without significantly shifting the corrosion potential (*E*_corr_) (by more than 85 mV). Their adsorption mostly obeyed the Langmuir isotherm model. Polyethylene glycol (PEG), DTAB, Tween-20, Polyoxyethylene (40) nonylphenyl ether (PNE), and hydroxyethyl cellulose (HEC) are employed as effective and eco-friendly substitutes to mitigate Zn anode corrosion [157, 164–167]. They exhibit 80–98% anodic dissolution inhibition efficiencies, depending on the nature of the electrolytes. Recent advancements in green chemistry and sustainable development necessitate the use of green, especially bio-based polymers, such as PEG and HEC, for anticorrosion applications in batteries. They are highly flexible, hydrophilic, cost-effective, and compatible, with remarkable film-forming and ionic conductivity properties.

The idea of using bio-based additives was further explored by Yang et al. [168]. They observed that CMC (carboxymethyl cellulose) passivates the Zn surface and retards dendrite growth in 6 M KOH. CMC forms a non-porous, uniform hydrophilic-hydrophobic interface layer that prevents the diffusion of corrosion species, reduces *C*_R_ and *i*_corr_, and enhances *R*_ct_. The formation of protective hydrophobic films of 2-octanone ethylene diamine (OED) via coordination bonding was studied in a separate study [169]. The results showed that a 4 wt% loading of OED manifested more than 95% efficiency. OED minimized the risks of pitting corrosion by providing a uniform layer. The H_2_ evolution, i.e., cathodic reactions, was significantly reduced, as OED primarily serves as a cathodic-type inhibitor. The polymer-based inhibitors mostly form a uniform and compact monolayer, as indicated by the Langmuir isotherm [170, 171]. The inhibitor films provide 90%–96.8% anodic dissolution inhibition efficiency. The use of surfactants and polymers can be considered as Hg-free, next-generation, amphiphilic, sustainable alternatives for corrosion protection in battery systems.

The literature survey reveals that several corrosion inhibitors have been developed and employed in battery systems. Their modes of action can be rationalized within a combined mechanistic classification framework. The different inhibitors described in the present section can be divided into three classes: (i) adsorption and film-forming surfactants and polymers, including DTAB, PEG, Tween-20, HEC, CMC and PNE, which produce corrosion-protective physical barrier the interface and retard anodic Zn corrosion and cathodic H_2_ evolution; (ii) chelating and multidentate heterocycles, including benzotriazole and imidazole derivatives, BHB, nitrilotriacetic acid (NTA) and gluconate-based complexes, which coordinate with metallic and ionic Zn sites and form highly protective chelated protective films through chemisorption mechanism; and (iii) inorganic or ionic modifiers, including Sc^3+^ cations, Nb-based treatments, oxygen scavengers (AQS) and sulfate salts, which control interfacial charge distribution, nucleation behavior and composition of passive films [172, 173].

Their molecular and structural properties primarily determine their effectiveness as protectants. Corrosion inhibitors containing electron-rich polar functional groups of heteroatoms (N, S, O), π-conjugated systems in the form of aromatic rings or side chains, and multiple adsorption sites with the potential for chelation significantly enhance adsorption effectiveness through chemisorption on the ionic or metallic Zn surface. The adsorption of corrosion inhibitors and the formation of hydrophobic protective films are consistent with improved coulombic efficiency, increased *R*_ct_ (or *R*_p_), and decreased icorr. In polymer- and surfactant-based corrosion inhibitors, the balance between hydrophilic segments (headgroups) and hydrophobic segments (alkyl chains) determines wettability, ion transport, and the compactness of protective films, and directly affects dendrite formation and H_2_ evolution. Generally, hybrid formulations having polymers (or organic) components mixed with inorganic ions provide synergistic protection by suppressing both anodic and cathodic reactions.

A noticeable transition occurred between 2009 and 2018, as ZnSO_4_-based systems replaced alkaline batteries, enabling rechargeable Zn-ion batteries with enhanced reversibility, safety, and stability. Sun et al. manifested that sodium anthraquinone-2-sulfonate (AQS) behaved as a self-oxidizing oxygen scavenger [174]. AQS achieves a coulombic efficiency of over 99% and cycling stability of more than 2500 h. Through the deoxygenation mode, AQS also retards dendrite growth, surface precipitation, *C*_R_, and *i*_corr_, enabling long-lasting cycling. Gelman and coworkers demonstrated that the PEG-Zn hybrid systems form a significantly denser, more uniform protective film [175]. Hybrid systems not only improve corrosion resistance but also enhance battery performance and lifespan [175, 176]. The use of hybrid systems (organic/polymers and inorganic additives) has also been explored elsewhere [177–179]. The presence of Pb^2+^ and Mg^2+^ ions improves the inhibition performance of pyrazole and gelatine, respectively. In Zn-air and Zn-alkaline systems, amphiphilic inhibitors address long-standing problems by suppressing H_2_ evolution, dendrite growth, and ZnO passivation. Many surfactants, including SDS, Tween-20, and SDBS, have shown noticeable improvements in the discharge performance and electrochemical stability of the anode materials [180–182]. These surfactants served as interfacial regulators, diminishing electrode polarization, improving ion transport, and stabilizing the anodes.

**Fig. 6:**
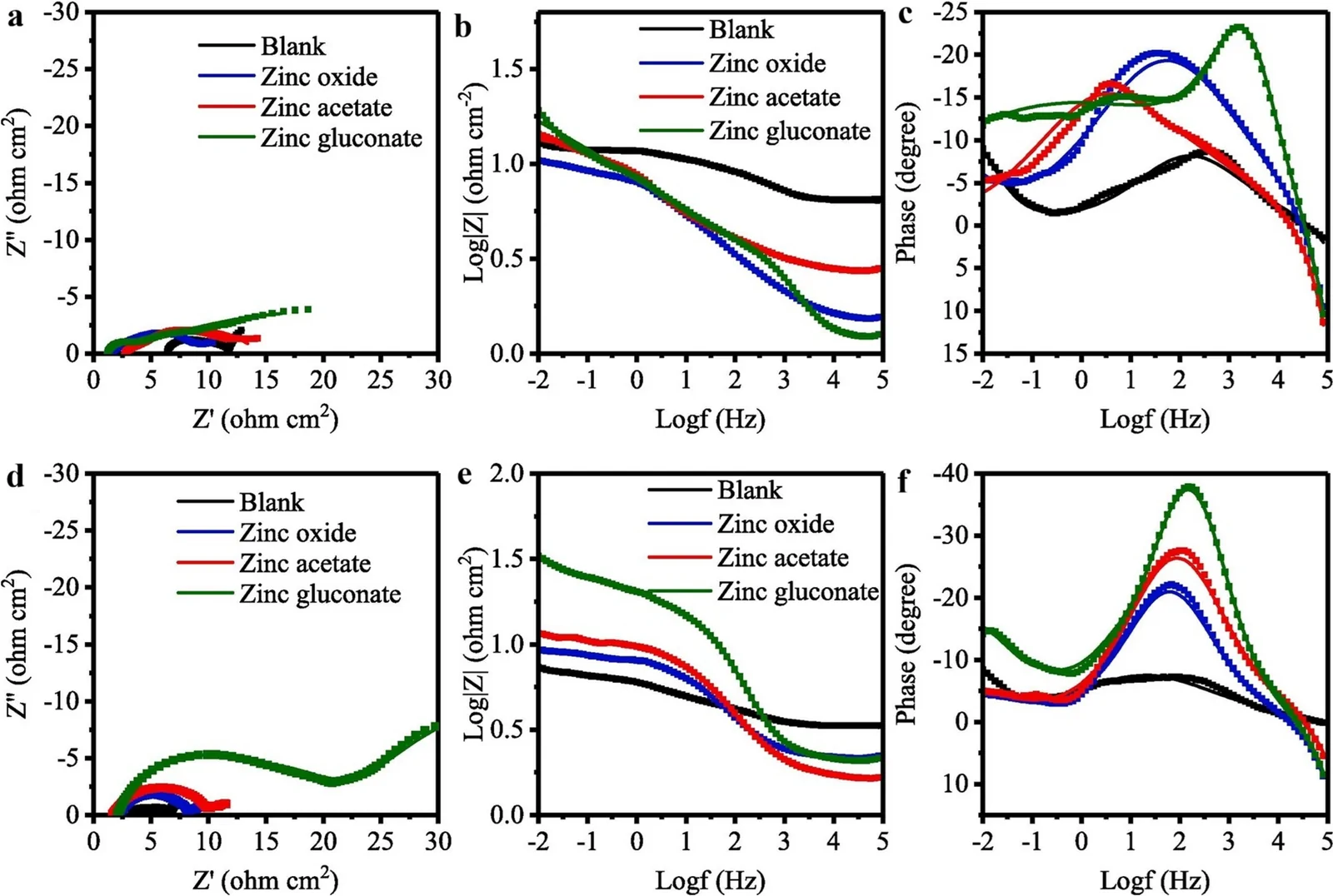
Fitted findings and EIS test curves for the zinc anode following immersion in four electrolytes for **a**–**c** 0.5 h and **d**–**f** 24 h [183]. (Reproduced with permission. © 2024 Elsevier)

**Fig. 7:**
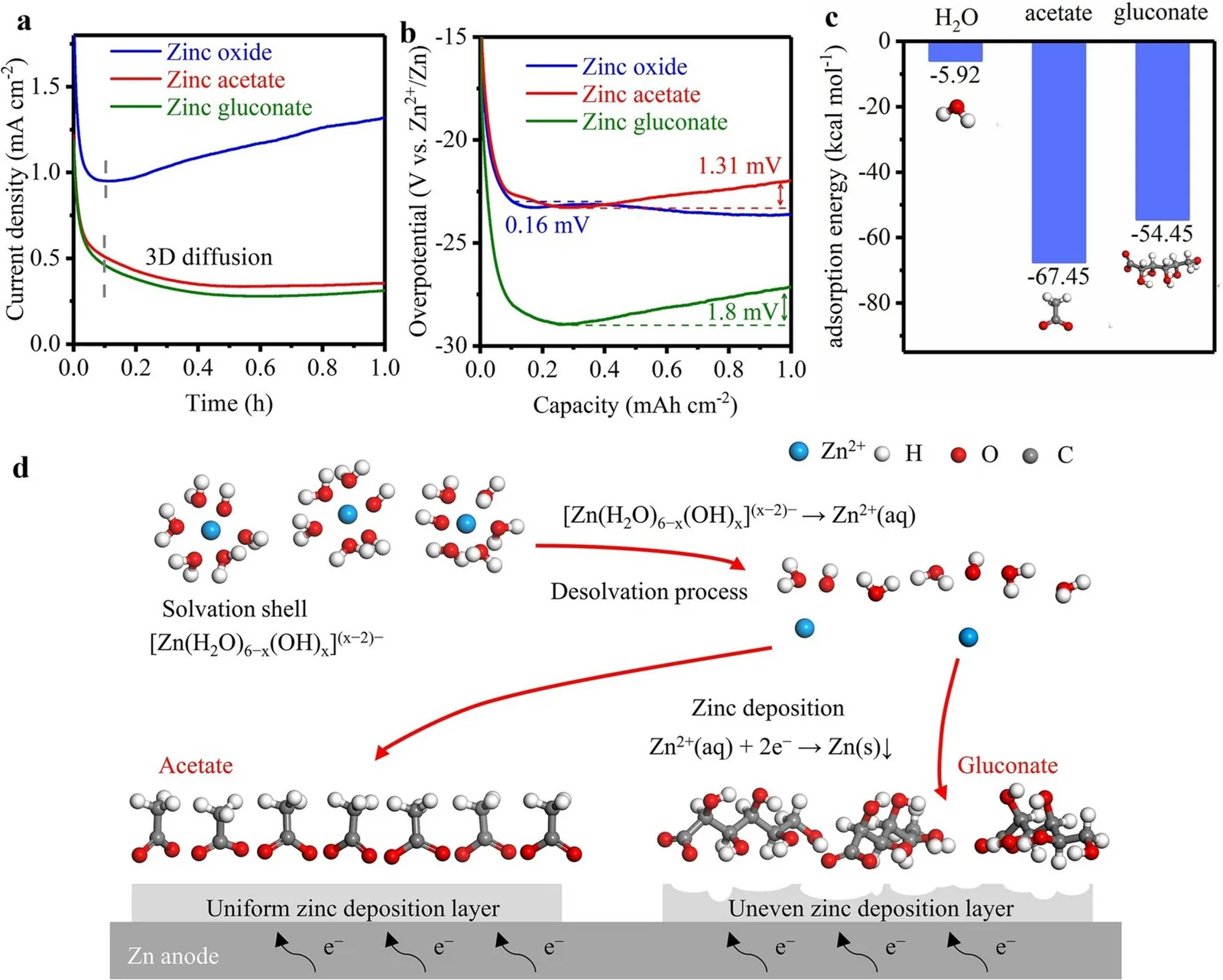
**a** Zn anode CA and **b** CP measured in various electrolytes. **c** The energy of adsorption of gluconate, acetate, and H_2_O on the Zn (0 0 2) plane. **d** Additives and the zinc deposition process schematics [183] (Reproduced with permission. © 2024 Elsevier)

Li et al. proposed using a more complex amphiphilic PEI (polyethylenimine) inhibitor for dendritic suppression and corrosion protection [184]. A relatively noticeable transformation was the use of coordination-complex-based corrosion inhibitors in battery systems. The counterions of these complexes not only regulate the nucleation but also stabilize the redox reactions. We et al. studied the corrosive dissolution of Zn foils in an alkaline solution of ZnO with and without Zn acetate (ZA) and Zn gluconate (ZG) [183]. The Nyquist and Bode plots for Zn corrosion in 6M KOH with and without ZA and ZG are shown in Fig. 6. The careful inspection reveals that, initially, inhibitors exhibit incomplete adsorption; however, after 24 h of immersion, a complete adsorption film can be inferred from the development of a well-defined capacitive semicircle in Nyquist plots. ZG produced the largest diameter with the highest phase angle (~ 37°) among the studied systems, indicating the best resistance, most effective adsorption, and thickest inhibitive films. The nucleation of Zn^2+^ ions in an alkaline solution containing ZA and ZG, which proceeds mainly via 3D diffusion, is schematically measured through chronoamperometry (CA) and chronopotentiometry (CP) and is shown in Fig. 7a, b. Figure 7c shows that acetate ions bind effectively to the 002 Zn surface by replacing the pre-adsorbed H_2_O molecules, thereby reinforcing the deposition and desolvation of Zn^2+^ ions. The adsorption and formation of protective film by gluconate and acetate ions are illustrated in Fig. 7d. They form a uniform, non-porous protective film that prevents the diffusion of corrosive species.

Recently, advanced molecules for mitigating anodic dissolution, including polyols, ionic liquids (ILs), and metal–organic frameworks (MOFs), have emerged as practical and multifunctional alternatives [64, 173, 185, 186]. MOFs provide anticorrosion protection and enhance battery performance, achieving more than 85% capacity retention and 99.6% Coulombic efficiency even after over 6000 cycles. ILs form a corrosion-protective hydrophilic film, providing more than 91.3% efficiency at concentrations as low as 2.5 mM. MOFs and ILs retards the dendrite growth, minimize sulfate precipitation, and homogenize the metallic surface. The use of ion-assisted and functionalized electrolytes can be considered another notable transformation in protecting battery anodes through solvation, film formation, and ion-surface interactions. Kim and coworkers proposed that Sc^3+^ cations force uniform Zn deposition, which retard dendrite growth [187]. The Sc^3+^ cations' “tip-blocking” mode of action was associated with a coulombic efficiency exceeding 99% over 100 cycles. The polyhydroxylated sugars (polyols), such as trehalose, develop an H-bonding network and minimize the presence of free water molecules [188]. The coordination of trehalose with Zn^2+^ and the subsequent hydrogen-bonding networks decreased cathodic H_2_ evolution and anodic Zn dissolution, thereby promoting Zn deposition. The organic and ionic components regulate the deposition and solvation kinetics.

Nowadays, artificial intelligence (AI) assisted screening has emerged as one of the most potent and effective tools in electrolyte optimization and materials discovery in energy storage systems [189, 190]. High-throughput computational screening using machine learning (ML) and data-driven modeling approaches enables the assessment of several vital parameters, including binding affinity, electrochemical stability window (ESW), solvation structure, hydrogen evolution activity, and adsorption energy [191–195]. Nowadays, ML-driven studies on proton-conducting electrolysis and H_2_ evolution electrocatalysts have shown that data-driven strategies can effectively tailor or fabricate active-site distributions, electronic structures, and overall surface chemistry to improve interfacial stability and reduce HER kinetics [196, 197]. Such approaches provide useful insight for designing effective corrosion-resistant electrolytes for aqueous battery systems. AI models can also be used to correlate molecular descriptors such as energies of frontier molecular orbitals, i.e., energy of highest occupied molecular orbital (*E*_HOMO_), energy of lowest unoccupied molecular orbital (*E*_LUMO_), their difference Δ*E* (*E*_LUMO_ − *E*_HOMO_), electronegativity (*χ*), dipole moment (*μ*), and hydrogen-bonding potential, to design corrosion-resistant electrolytes. These computed parameters can be correlated to experimentally derived parameters such as inhibition efficiency (%IE), *R*_ct_, *R*_p_, or *i*_corr_. Fortunately, computational approaches enable the identification of effective electrolyte additives and corrosion inhibitors without the tedious synthesis and experimental trials [198–200].

For example, DFT-derived parameters combined with ML algorithms can be used to predict the adsorption nature and effectiveness of corrosion inhibitors on electrode (Mg, Al, Zn, etc.) surfaces. In contrast, molecular dynamics (MD) and Monte Carlo (MC) simulations can be used to study water activity and solvation structure in gel-based or concentrated electrolyte systems [198–200]. AI can also be used to gain helpful insight about the selection of optimized anions by predicting pitting susceptibility and oxide film stability under different operating conditions, such as varying voltage, temperature, and pH [201, 202]. Moreover, predictive models developed using electrochemical datasets may help define safe and effective operational windows under extreme conditions, i.e., high temperature and voltage, by linking corrosion kinetics to electrolyte composition. Given this, combining electrochemical outcomes and mechanistic modeling and AI-driven strategies offers a promising pathway toward designing and developing corrosion-resistant electrolytes and corrosion inhibitors for the next-generation sustainable batteries.

**Fig. 8:**
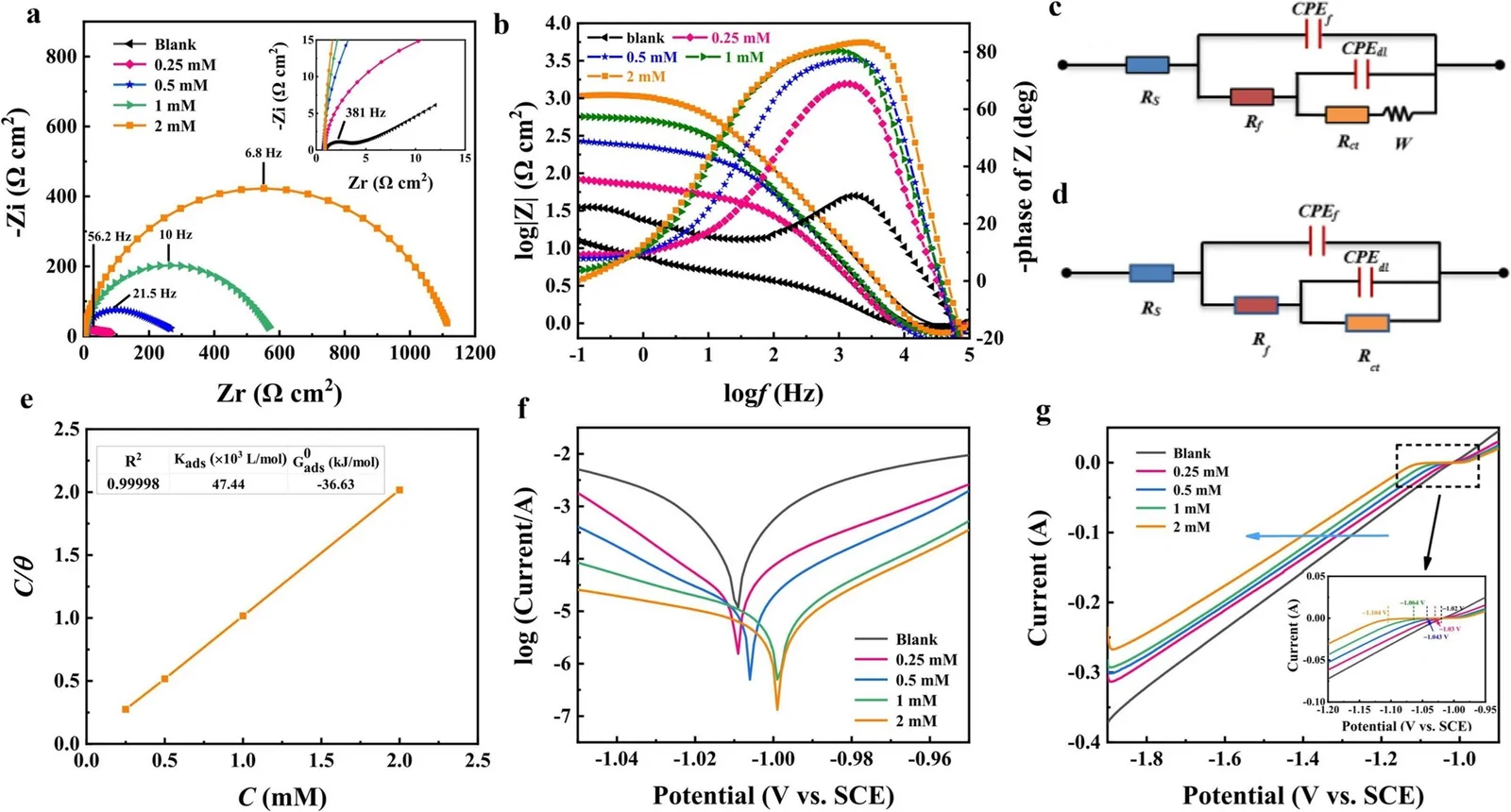
The zinc electrode was submerged in a 2 M ZnSO_4_ solution, with and without BHB. **a** Nyquist graph, **b** Bode plots, **c**, **d** equivalent circuits, **e** Langmuir isotherm adsorption curves, **f** linear polarization curves, and **g** LSV curves [206] (Reproduced with permission. © 2023 Elsevier)

Inorganic and anionic modifications further expanded the design of corrosion-resistant electrolytes. Lin et al. observed that the presence of SPS (anionic sodium 3,30-dithiodipropane sulfonate) in corrosive alkaline solution forced horizontal Zn growth through the selective adsorption of sulfonate groups available at the high-energy sites, favoring the development of corrosion resistance orientation [203]. This type of orientation enabled dendrite-free cycling for over 4400 h, with a coulombic efficiency exceeding 99.7%. Likewise, Na_2_SO_4_ facilitates the formation of passive films over the Zn surface, minimizing *C*_R_, H_2_ evolution, and *i*_corr_ [204]. Currently, considerable attention is being paid to the development and application of multidentate heterocyclic and chelating inhibitors for zinc stabilization in aqueous battery systems. NTA (nitrilotriacetic acid) serves as a multidentate additive that coordinates Zn^2+^ and develops chelating films (NTA-Zn^2+^), reducing Zn(OH)_2_ formation and cathodic H_2_ evolution [205]. 0.15 wt% NTA reduced *i*_corr_ by more than 90% and achieved a coulombic efficiency of 99.4% after 800 cycles. NTA also promotes uniform Zn deposition and Zn^2+^ desolvation. Similarly, BHB (6-bromo-1H-benzimidazole) coordinates to the Zn surface via its Br and N atoms, promoting effective film formation and retarding H_2_ evolution and dendrite growth [206]. A coulombic efficiency of 99.24% was observed after 1150 h in the presence of 0.25 mM. BHB greatly enhanced corrosion resistance. The Nyquist and Bode plots, which reveal a significant increase in corrosion resistance, are shown in Fig. 8a, b.

**Fig. 9:**
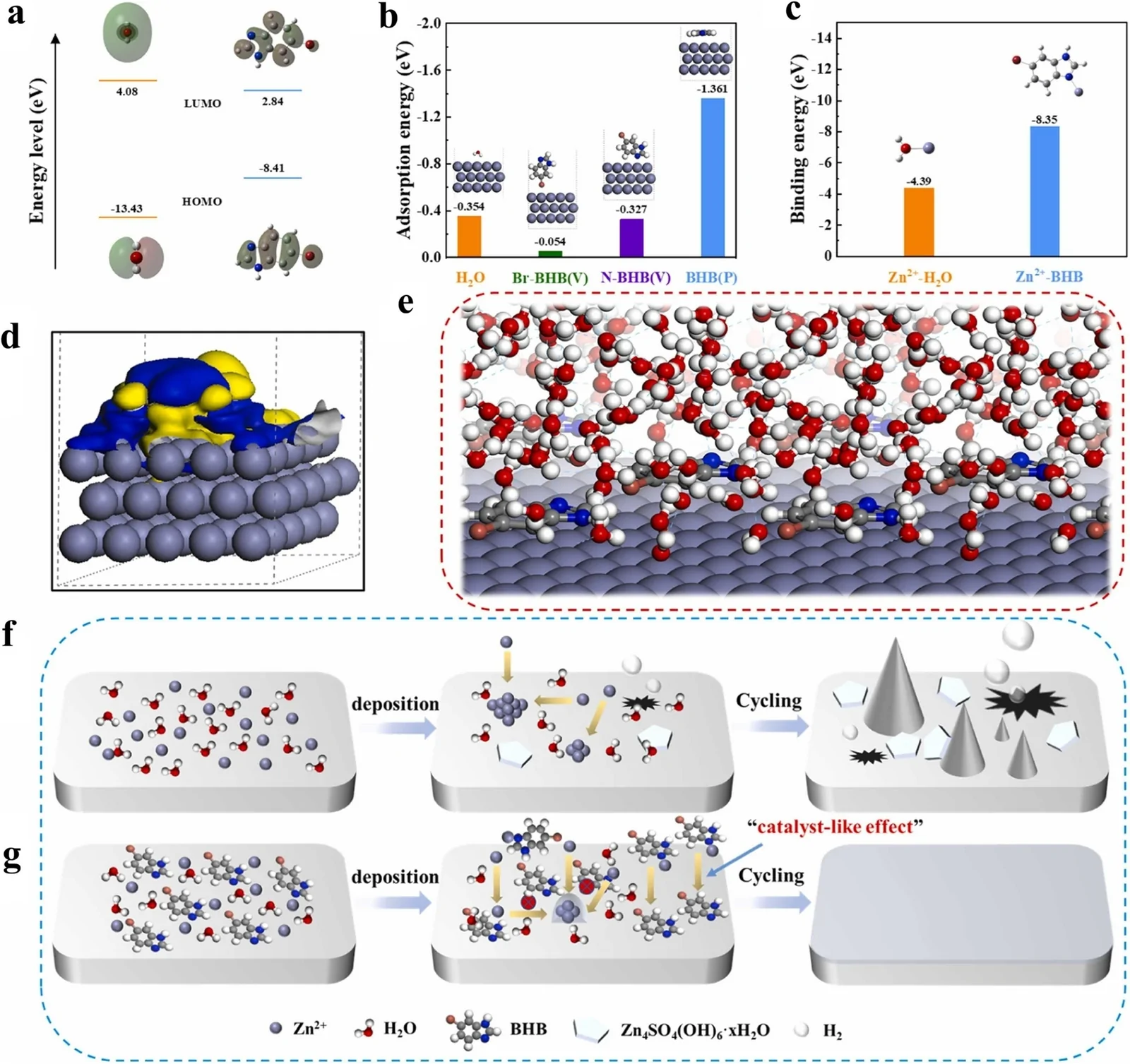
**a** HOMO and LUMO for molecules of BHB and water. **b** Water and BHB adsorption energy on the zinc surface. **c** Zn^2+^-H_2_O and Zn^2+^-BHB complex binding energies. **d** Map of differential charge density, where blue indicates decreasing charge density, and yellow indicates rising charge density. **e** MD simulation snapshot. The schematic diagrams of Zn deposition behavior for ZnSO_4_ and ZnSO_4_ + BHB electrolytes are shown in **f** and **g**, respectively [206] (Reproduced with permission. © 2023 Elsevier)

The Nyquist curves were suitably fitted in equivalent circuits to get the desired EIS parameters (Fig. 8c, d). The increase in R_ct_ and impedance values indicated adsorption and the formation of a protective BHB film. The adsorption was also supported by high film resistance (*R*_f_) and lower double-layer capacitance (*C*_dl_). The adsorption of BHB followed the Langmuir isotherm (Fig. 8e), suggesting that it forms a chemisorbed monolayer. Potentiodynamic polarization (PDP) curves in the presence of BHB showed a slight decrease in H_2_ evolution and *i*_corr_, with a protection efficiency of approximately 99% (Fig. 8f, g). Computational studies support the interactions between the Zn surface and BHB. The differential charge density map reveals electron accumulation around Br and N and depletion on Zn sites, thereby establishing coordination and charge transfer processes. MD simulation analyses showed that BHB forms a robust monolayer through parallel adsorption, resulting in a compact hydrophobic layer that prevents the diffusion of corrosive species, including water (Fig. 9a-e). Figure 9f, g shows the Zn growth behavior with and without BHB. The uniform, smooth, and dendrite-free Zn growth was clearly revealed in the presence of BHB, indicating homogenous nucleation and prolonged surface stability.

**Fig. 10:**
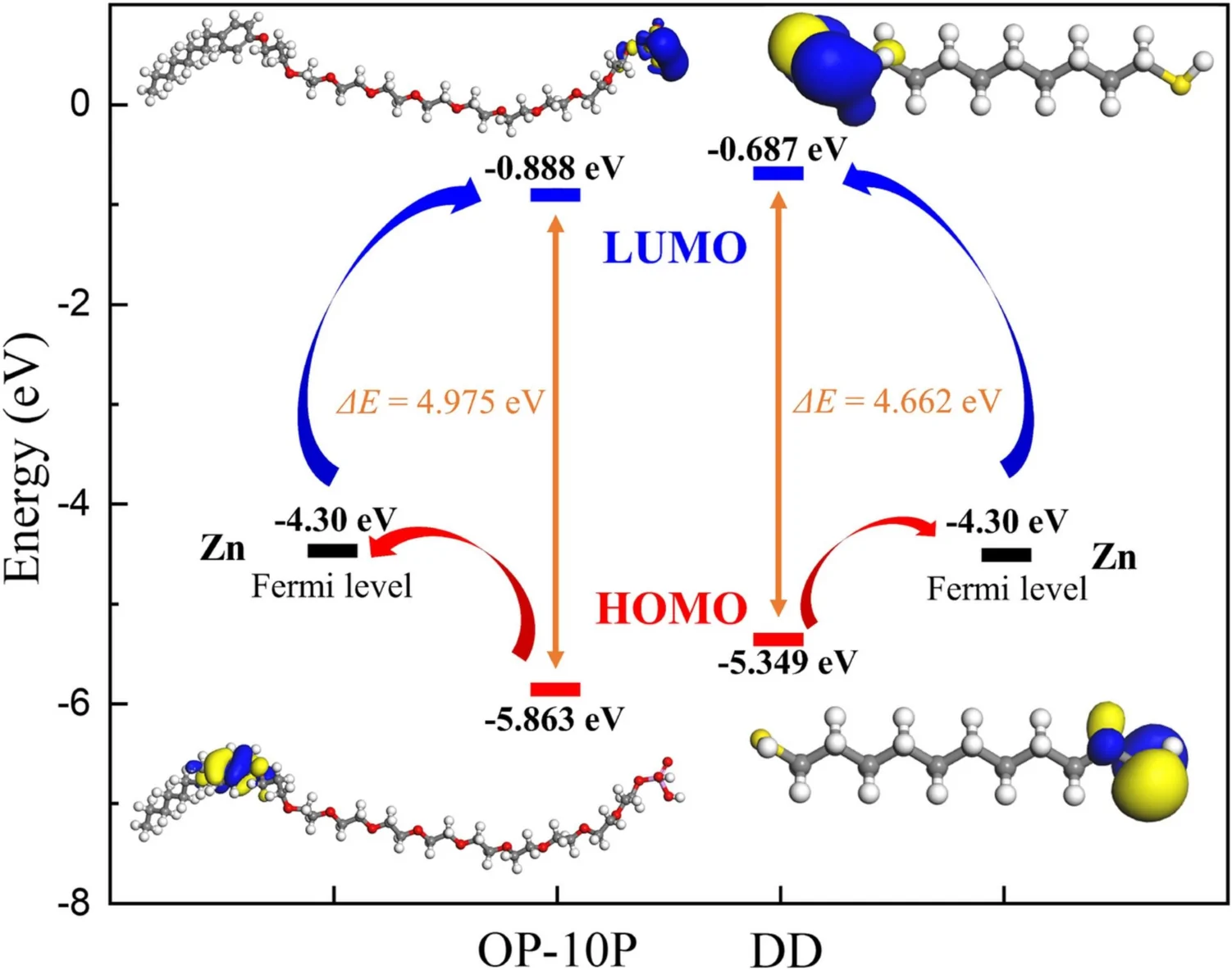
Schematic presentation of the EHOMO, ELUMO, and ΔE for OP-10P and DD are among the pertinent parameters that are computed in quantum chemistry [207] (Reproduced with permission. © 2024 Elsevier)

Nearly complete corrosion protection was observed using a dual-layer surfactant system (OP-10P) for the Zn-air battery [207]. DD (1,10-decanedithiol) coordinates with Zn^2+^ and forms an outer, protective, hydrophobic layer that reduces self-discharge and enhances conductivity during cycling. The electronic properties of OP-10P and DD were investigated using DFT. FMOs and their respective energies and energy gaps are illustrated in Fig. 10. DD has a higher *E*_HOMO_ value, favoring more donation or coordination, whereas OP-10P is more susceptible to better acceptance due to a lower *E*_LUMO_ value. The relatively low ΔE values for OP-10P and DD indicate that they chemisorb to the metal surface, forming uniform, compact corrosion-protective films. Likewise, the N-atom of Pyr (pyrazine), Pym (pyrimidine), and Pyd (pyridazine) coordinates with the Zn surface and forms corrosion-protective, hydrophobic films [208]. The DFT study revealed that pyrimidine with N atoms at 1st and 3rd (meta-) position, favored parallel adsorption, balancing and controlling the coordination and desolvation kinetics.

The use of bio-based inhibitors has been identified as a promising new avenue for sustainable anode stabilization and corrosion protection. Ren et al. reported that fucoidan (FCD), a natural polysaccharide, forms an 8 nm covalently bonded film on a metallic substrate by coordinating through its oxygen atoms [155]. The covalently monolayer film so formed reduced the H_2_ evolution and retard the corrosion without affecting the Zn^2+^ transport, providing up to 2700 h cycling. The bio-based chelating FCD revealed a coulombic efficiency of 99.5% after 300 cycles. Likewise, protonated triglycine (ggg), a zwitterion, interacts with the Zn surface through –NH_3_ and –COO^−^ and reduced water activities and H_2_ evolution [209]. The use of biocompatible compounds in Zn anode corrosion protection has also been explored in other reports [210, 211]. Organophosphonates, polyols, and metal–organic complexes have been established as effective multifunctional additives for Zn-based aqueous batteries [212–214]. They provide anticorrosion protection by adsorbing at the interface of the Zn surface and aqueous electrolytes. They reduce the *C*_R_, *i*_corr_, and H_2_ evolution and enhance R_ct_ for Zn corrosion in such electrolytes. They also improve the battery performance, including coulombic efficiency over 99%.

The use of complexing and film-forming additives for enhanced stability and corrosion protection has been further advanced in recent studies. Sn and coworkers developed dopamine-derivatized polypyrrole (DA-PPy) for H_2_ evolution reduction and corrosion protection [215]. DA-PPy, with its numerous coordination sites, effectively binds to the surface, retarding corrosion without affecting Zn^2+^ transport. Zn-pyrrolidone carboxylate (Zn-PCA) stabilizes cathodic and anodic processes in Zn-I_2_ batteries [216]. Polyiodide adsorbs and reduces dendrite growth and corrosion, with 87% capacity retention after 30,000 cycles. PCA- anions mainly coordinated with I_2_. Imidazo[1,2-b]pyridazine (IP) provides dual functionalities, i.e., anchoring and shielding effects [217]. The coordination and chemisorption of IP via N atoms displace pre-adsorbed water molecules. This results in dendrite-free deposition and stable cycling over 2200 h with a very high coulombic efficiency of 98.7%.

**Fig. 11:**
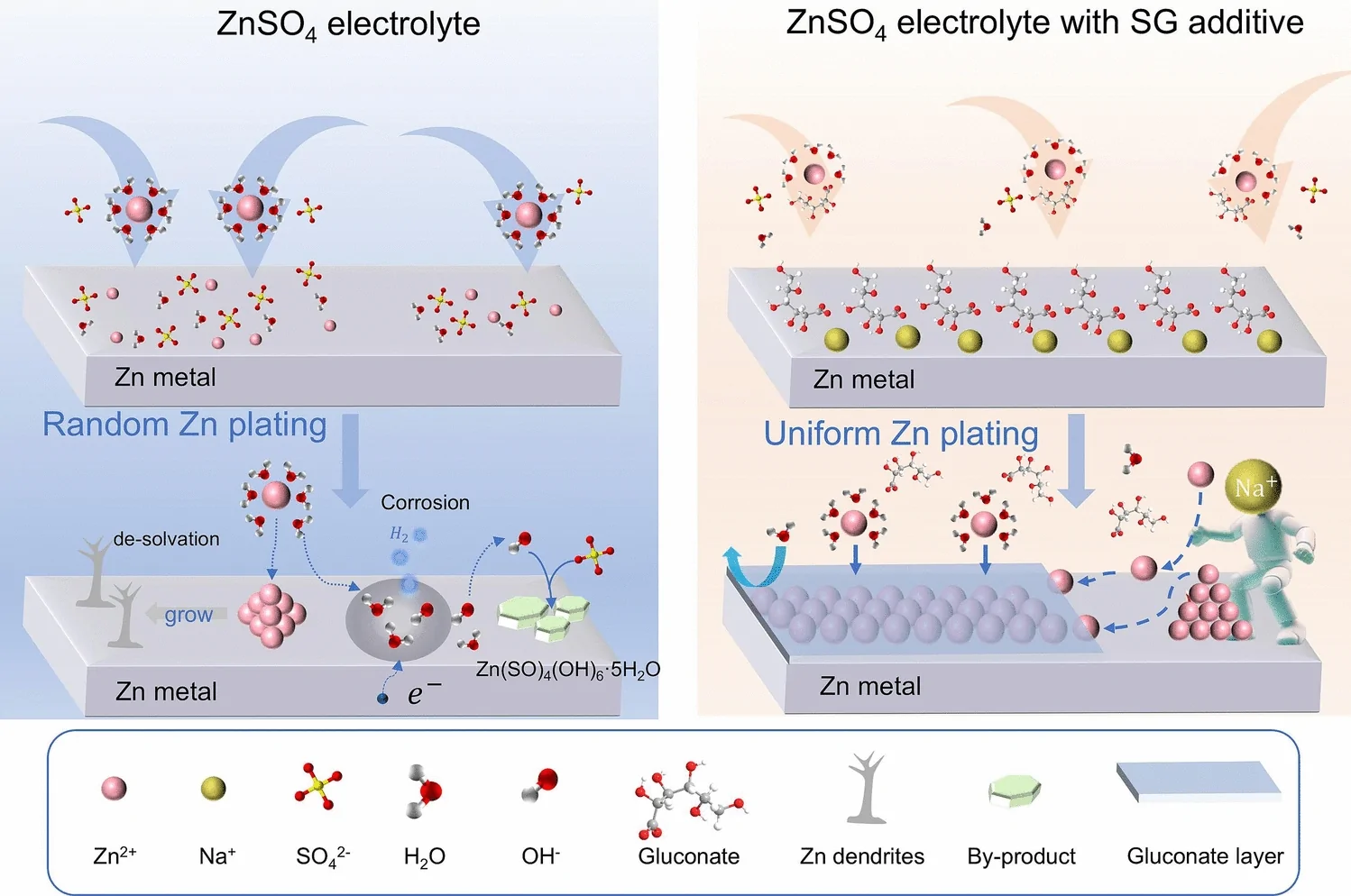
Diagrammatic representation of SG and the related mechanisms of action [218] (Reproduced with permission. © 2024 Elsevier)

**Fig. 12:**
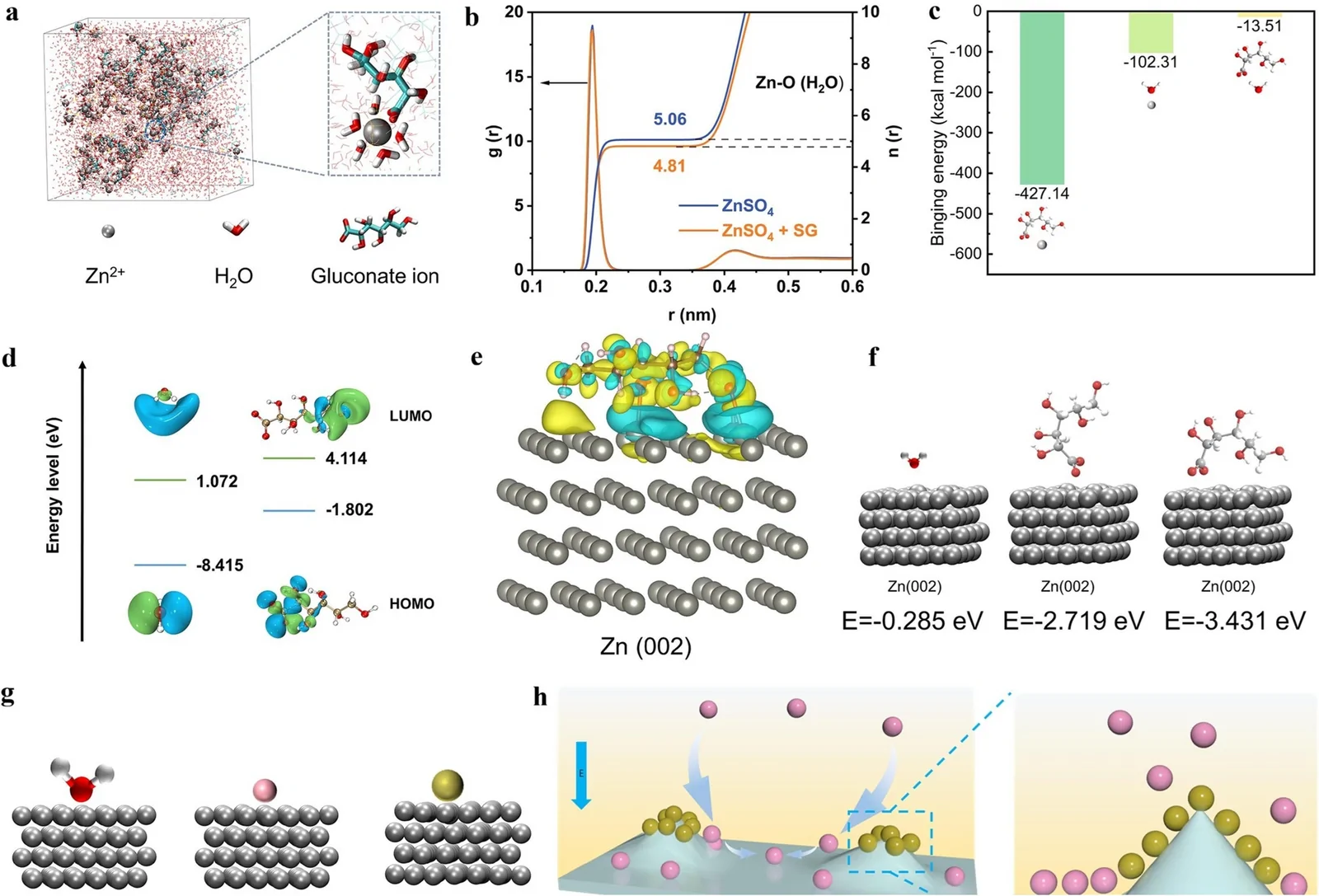
**a** A partial magnified image depicting the Zn^2+^ solvation structure and a 3D photo of the ZnSO_4_-SG system derived from MD simulations. **b** RDFs for Zn–O (H_2_O) obtained from MD simulations of ZnSO_4_-SG electrolyte. **c** Zn^2+^ binding energy with gluconate and H_2_O molecules as determined by DFT. **d** LUMO and HOMO levels of gluconate and the H_2_O molecule. **e** 3D isosurface diagrams showing the adsorption energy and charge density differential of SG (parallel) on Zn foil. **f** Comparison of gluconate and H_2_O absorption energies on the Zn (002) crystal plane. **g** H_2_O, Zn^2+^, and Na^+^ adsorption energies on the Zn (101) crystal plane. **h** Schematic illustration of how Na^+^ suppresses Zn dendrites by electrostatic shielding [218] (Reproduced with permission. © 2024 Elsevier)

Ma et al. elucidated sodium gluconate (SG) as a cost-effective and multifunctional filming corrosion inhibitor for Zn corrosion [218]. Gluconate anions form a chelating complex that prevents the attack by corrosive species, including SO_4_^2−^ ions. In the presence of these anions, the solvated Zn^2+^ converts into [Zn(gluconate)(H_2_O)_5_]^+^, which reduced C_R_ and H_2_ evolution. The working mechanism of SG is illustrated in three steps in Fig. 11. The adsorption of gluconate ions produces corrosion-preventive films that avoid direct water contact and attack. GS guided uniform Zn plating via electrostatic shielding of Na^+^ and SO_4_^2−^ ions at the interface. The replacement of H_2_O molecules by gluconate ions results in the formation of new solution shells; Zn(gluconate)(H_2_O)_5_]^+^. These result in reduced byproduct production, dendrite growth, C_R_, and improved uniformity in Zn deposition. MD simulations and DFT analyses revealed that SG replaced H_2_O molecules in the solvation shells, enabling the formation of stable complexes and preventing corrosion (Fig. 12). The *E*_binding_ for gluconate and Zn^2+^ was much greater than that of the water and Zn^2+^ system. The adsorption of SG follows a chemisorption mechanism retarding corrosion. Moreover, Na^+^ ions enhance Zn^2+^ nucleation through electrostatic field modulation and preferential adsorption, thereby reducing dendrite growth.

**Fig. 13:**
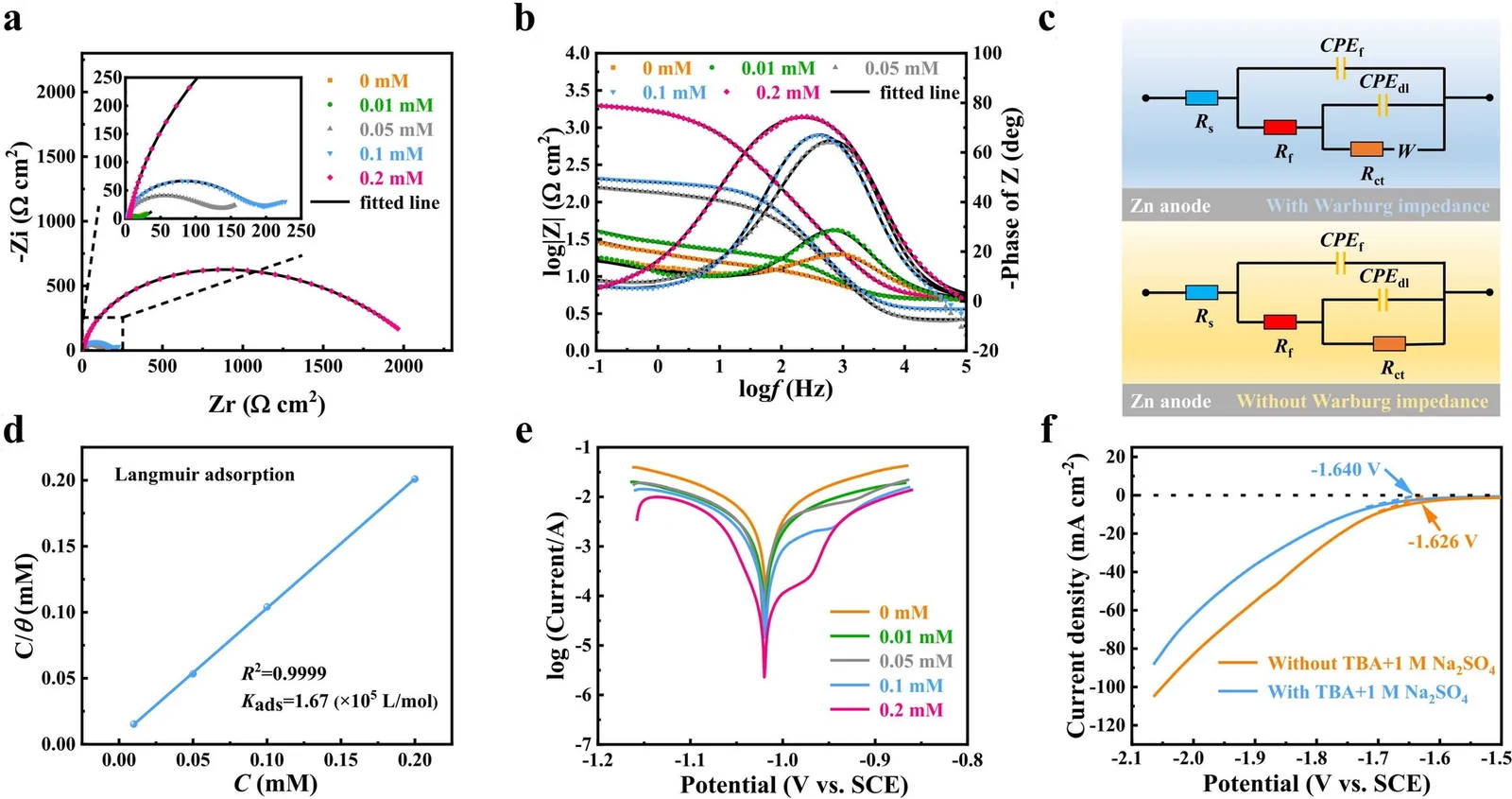
**a** Nyquist curve, **b** Bode curve,** c** Equivalent circuit diagram, **d** Langmuir isothermal adsorption curve, and **e** Tafel diagram of the Zn electrode in ZSO solution with varying TBA concentrations. **f** LSV curve [219] (Reproduced with permission. © 2025 Elsevier)

The use of advanced molecules such as urotropine (URT) and 2-amino-6-trifluoromethoxybenzothiazole (TBA) in stabilization and corrosion protection of Zn anodes gained significant advancement [219, 220]. Le et al. demonstrated that TBA manifests remarkable capability by developing a zincophilic-hydrophilic interface coordinating with a benzothiazole anchor moiety and a terminal trifluoromethoxy group [219]. These result in steric shielding and selective chemisorption at the Zn-electrolyte interface. The electrochemical behavior of the Zn surface in 2 M ZnSO_4_ solution with and without TBA is shown in Fig. 13. The larger diameter of the Nyquist plot semicircle in the presence of TBA suggests an increase in *R*_ct_ due to surface passivation (Fig. 13a). The Bode plots exhibit broadening of the phase angle and an increase in the impedance modulus, indicating enhanced capacitive properties and the formation of protective films at the Zn-ZnSO_4_ electrolyte interface (Fig. 13b). The equivalent circuits used for fitting the EIS graphs are shown in Fig. 13c. TBA becomes effective by adsorbing onto the metallic surface, following the Langmuir isotherm model (Fig. 13d). PDP analyses showed that TBA decreased *C*_R_ and *i*_corr_ without a significant shift in *E*_corr_, indicating that TBA is a mixed-type inhibitor (Fig. 13e). Lastly, LSV curves showed that the H_2_ evolution potential shifted slightly to more negative values, suggesting reduced H_2_O decomposition and H_2_ evolution. The increased *R*_ct_, decreased *C*_R_, and *i*_corr_ values suggest that TBA becomes effective by blocking the surface-active sites through chemisorption.

Recently, the use of anticorrosive coatings to enhance corrosion resistance, electrochemical stability, durability, and rechargeability of the Zn anodes is gaining particular attention in battery technology [237]. Metal oxide-based anticorrosive coatings, including TiO_2_, Al_2_O_3_, CeO_2_, and ZrO_2_, have manifested remarkable performance in reducing *C*_R_, *i*_corr_, corrosion, and H_2_ evolution while unchanging the electrochemical nature and stability of batteries. These metal oxides form stable and inherent films that separate the metallic substrates from corrosive environments. Al_2_O_3_ coatings (1.5 wt%) of Zn surface prepared by ALD and sol–gel approaches reduced *i*_corr_ by almost 79% and enhanced the capacity retention to 85–89.4% [221, 222]. The literature study reveals that Al_2_O_3_ coatings have been widely used due to their strong adhesion, barrier properties, inertness, and ability to block electron transfer. Al_2_O_3_ promotes the uniform deposition of Zn^2+^, minimizes H_2_ evolution (HER), and corrosion rate. Titanium dioxide (TiO_2_)- based coatings have also been used to protect Zn anodes. Zhao et al. observed that TiO_2_ minimized the formation of Zn(OH)_2_, along with reducing the HER and C_R_ [223]. The TiO_2_-coated Zn anode showed nearly 85% capacity retention after 1000 cycles. TiO_2_ coatings also provide the blocking effects for O_2_ and H_2_O diffusion, minimizing the risks of localized corrosion [224]. Silica (SiO_2_) based coatings are generally achieved by chemical solution and chemical vapor deposition (CSD and CVD) techniques.

**Fig. 14:**
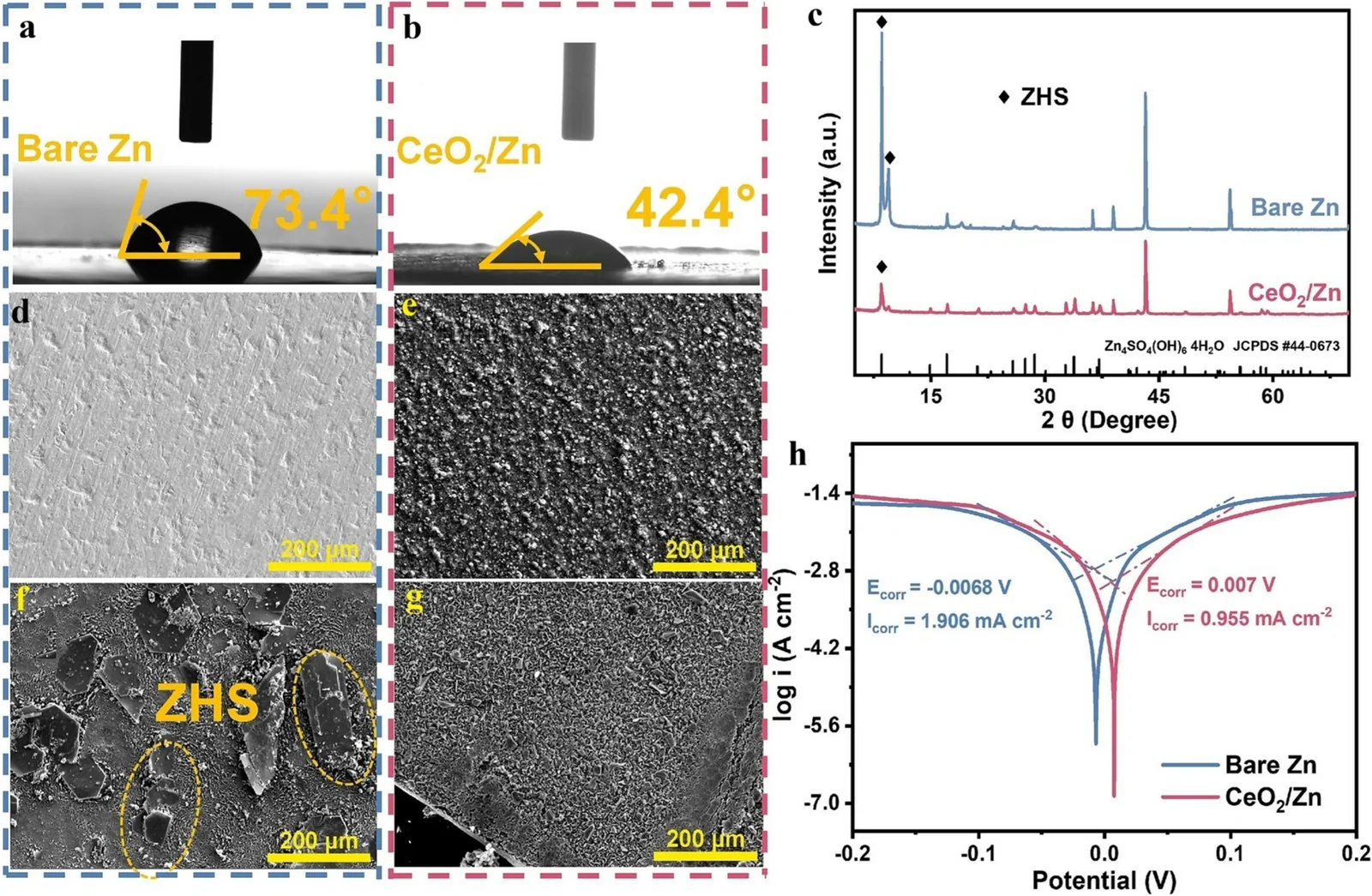
Schematics showing **a** bare Zn and **b** CeO_2_/Zn electrodes' water contact angle values. **c** Uncovered Zn and CeO_2_/Zn anode XRD patterns following a 7-day immersion in 2 M ZnSO_4_ electrolyte. Before and after immersion, SEM pictures of **d**, **e** bare Zn and **f**, **g** CeO_2_/Zn. **h** Tafel curves of the CeO_2_/Zn and bare Zn anodes [227] (Reproduced with permission. © 2025 Elsevier)

Zn_2_SiO_4_ (zinc silicate) or SiO_2_ coatings provide insulating properties while maintaining the electrochemical stability [225]. The highly adherent and compact layers of these coatings reduced HER, *C*_R_, and *i*_corr_. SiO_2_ coatings also provide chemical barriers by limiting the gas evolution and hydroxide (OH^−^) adsorption. The use of ZrO_2_ coatings for Zn anode protection is highly rated, as they improve electrochemical stability and mechanical integrity. Lian and coworkers showed that ZrO_2_ coatings stabilized the Zn-MnO_2_ cells [226]. The adherent ZrO_2_ layer promotes uniform nucleation of Zn^2+^ and acts as a physical barrier, preventing direct contact with the electrolyte. ZrO_2_ coatings also reduced ZnO densification, thereby improving the corrosion rate and coulombic efficiency to 99.35% after more than 3800 h. The development of CeO_2_ coatings for Zn anode protection can be considered one of the most advanced coating approaches, as Zn deposition along the (002) crystal planes reduces HER and dendrite growth [227]. CeO_2_ coatings also reduced the contact angle (Fig. 14a, b). XRD analysis reveals the formation of effective and adherent coatings (Fig. 14c). The SEM images of bare and CeO_2_-coated samples with and without immersion are shown in Fig. 14d–g. The PDP study revealed that the anodic and cathodic curves were significantly affected, and i_corr_ decreased notably with CeO_2_ coatings (Fig. 14h). The CeO_2_-coated anode shows 98.5% capacity retention after 4000 cycles. Overall, the CeO_2_ coatings improve kinetic stability, retard side reaction and formation of byproducts, and promote uniform Zn growth. Similar observations have also been reported in the literature for ZnNb_2_O_6_ (zinc niobate) and Al_2_Si_2_O_5_(OH)_4_ (kaolin)-based coatings [228, 229].

Organic and polymer coatings provide both physical and flexible barriers that protect Zn anodes from H_2_ evolution and corrosion, while retaining their excellent mechanical integrity and electrical conductivity. Jo et al. observed that PANI coatings prevent direct contact between the aqueous electrolyte and Zn particles [230]. The PANI coatings reduced the HER and self-discharge potential, maintaining a capacity retention of 97.8% after 24 h. The coatings demonstrated an effectiveness of 85% in providing corrosion protection. A similar observation was reported in another study involving PVDF-based coatings containing TiO_2_ [230]. The hybrid coatings enhance chemical shielding and electrochemical stability. Carbon-based coatings offer multifaceted benefits, including reduced HER, *C*_R_, and *i*_corr_, uniform Zn deposition, and improved mechanical integrity and electrical conductivity. In the Zn@C shell structure, the carbon shell protects Zn from corrosion in alkaline solution and retards the ZnO leaching [231]. Nearly 99% capacity retention was derived after 400 cycles for the Zn@C shell structure. A Zn surface coated with surface-modified CNT (sCNT) blended with Zn_2_SiO_4_ exhibits excellent corrosion resistance properties [232]. The sCNT coatings offer dendrite-free cycling with a coulombic efficiency of nearly 99.5%. Cellulose nanofiber (CNF) and graphene acid (GA)- based composite (CNF/GA) coatings demonstrated self-discharge retention of 99% after 24 h. Hybrid (organic and inorganic) coatings provide multifunctional protection for Zn anode in aqueous electrolytes [224, 228, 233, 234]. Some metal-based anticorrosive coatings have also been developed and tested, exhibiting slightly lower coulombic efficiency and capacity retention [235, 236].

**Fig. 15:**
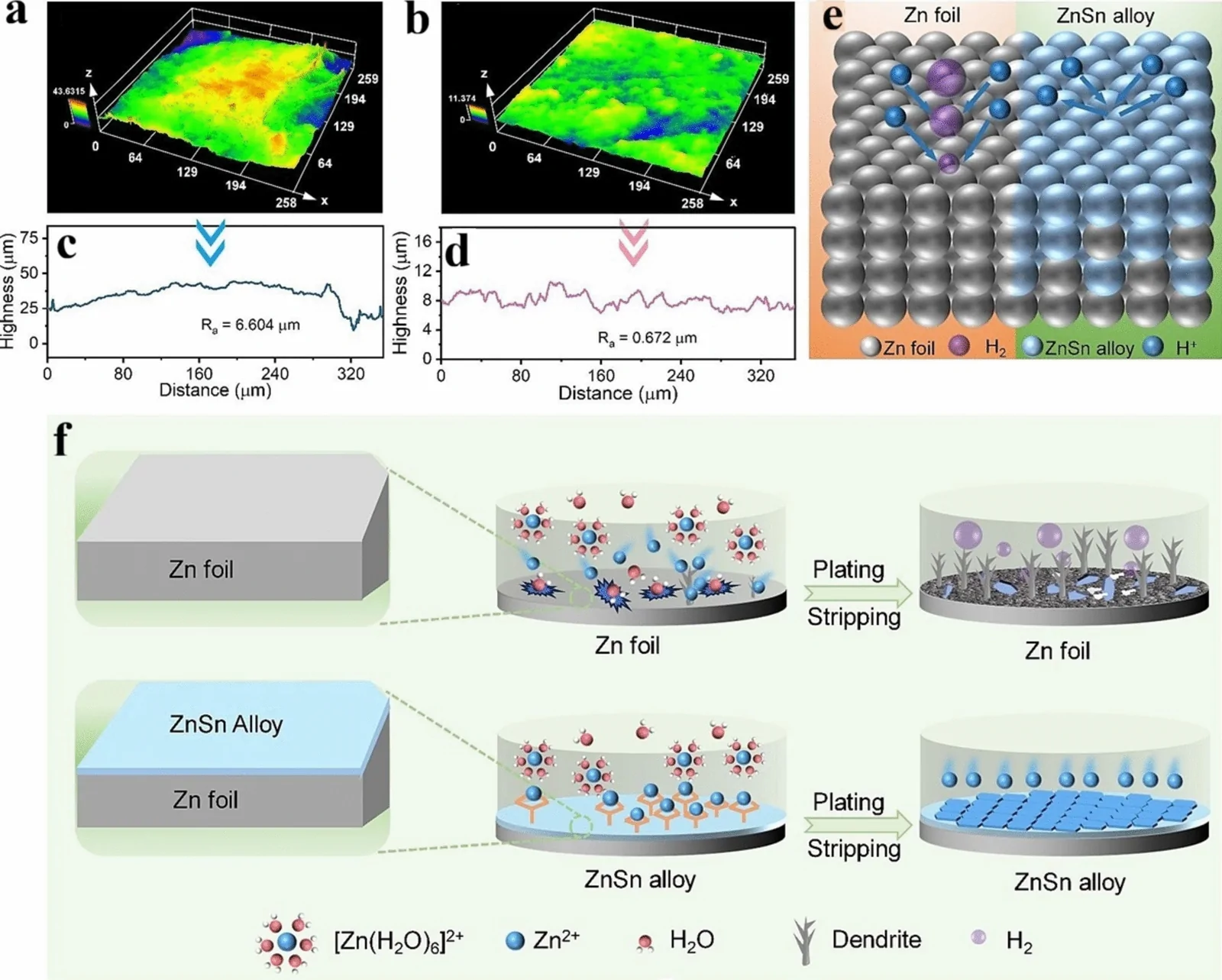
3D LCSM images of **a** Zn foil and **b** ZnSn alloy after 1000 cycles. **c** and **d** roughness profile. **e** Schematic diagram of H_2_ inhibition of ZnSn alloy. **f** Diagram of the modification mechanism of ZnSn alloy [245] Reproduced with permission. © 2025 Elsevier)

The corrosion resistance, H_2_ evolution, and electrochemical reversibility can also be tailored through alloying the Zn anode with suitable elements [238, 239]. The literature study reveals that the presence of Ni, Bi, and Sn additives significantly improves performance. Jo et al. showed that the presence of 1-5wt.% of Ni and Bi reduced the *C*_R_, *i*_corr_, and H_2_ evolution remarkably [240, 241]. A similar finding was reported elsewhere by Kang et al. [242], Da et al. [243], and Wang et al. [244] for Bi alloyed Zn anodes. The alloying not only improves corrosion resistance but also enhances battery performance, including capacity retention and high coulombic efficiency over 1000 cycles. Zhu and coworkers observed that the Sn-alloyed Zn anode showed 89.5% capacity retention [245]. They observed that for extended cycling, Sn alloying improves surface morphology and reduces H_2_ evolution. LCSM images revealed that after 1000 cycles, the bare Zn surface becomes highly rough, covered with corrosion products, and exhibits surface pits, cracks, and dendrite growth (Fig. 15a). However, Fig. 15b shows that the ZnSn alloy surface remains significantly smoother and more uniform, with fewer surface defects. Figure 15c, d represents the roughness profile of bare Zn- and Sn-alloyed Zn surfaces, and Fig. 15e, f shows the underlying mechanisms. ZnSn alloy serves as an H_2_ evolution inhibitor by reducing icorr and the H_2_ evolution overpotential. The dendrite formation and growth were significantly reduced by uniform Zn^2+^ nucleation. Summaries of recent reports on the corrosion inhibition of Zn anodes using corrosion inhibitors, anticorrosive coatings, and alloying are presented in Tables 4, 5, and 6, respectively.

**Table 4:** A summary of some recent reports on corrosion inhibition of Zn anode using corrosion inhibitors

S/N	Anode material	Battery type	Battery Electrolyte	Corrosion inhibitor(s)	Inhibition properties		Salient features	References
					Concentration	Efficiency		
1	Hg-free Zn powder	Alkaline Zn-MnO_2_ batteries	1 M KOH	Diphenylglyoxim, Tripropylene glycol, Diaminepyridine, 2,4-Dinitrophenol, PEG-200, and PEG-600	1.0%	NA	a) Reduced anodic dissolution b) Improved cycling c) Reduced H_2_ evolution	[159]
2	Hg-free pure Zn and Zn alloy (Mg &Pb)	Alkaline Zn-MnO_2_ and Zn-air batteries	10 M NaOH- additives	sodium stannate, CaO, and sodium citrate	0.02 M, 0.3 and 15 wt.%	Up to 99.0%	a) Hg-free anodes b) Reduced anodic dissolution c) Corrosion under anodic control (i.e., anodic-type inhibition)	[160]
3	Zn plate (99.99%)	Alkaline Zn-MnO_2_ and Zn-air batteries	ZnO saturated 8.5 M KOH	GAFAC RA600 (polyoxyethylene alkyl phosphate ester acid) and PEG-600	400–4000 ppm	RA600: 800–2000 ppm	a) PEG retard anodic reactions and H_2_ evolution b) Protection effectiveness increases with time	[161]
4	Zn powder coated with Nb	Alkaline Zn-based batteries	ZnO saturated 6 M KOH	Nb nitrate covering (Nb(NO_3_)_3_)	0.045 M Nb(NO_3_)_3_	90.9% (10 min US)	a) Uniform Nb-rich protective covering was formed b) Reduced corrosion current density (i_corr_) c) Increased transfer charge resistance (R_ct_)	[162]
5	Zn (99.35%)	Zn-Mn dry batteries	1 M ZnCl_2_, 1 M NH_4_Cl and their mixed	Amidopoly Ethylamines	1000 ppm	ZnCl_2_ (84%; Comp-IV), NH_4_Cl (68%; Comp-IV), and 79.8%	a) The inhibitors served as mixed types and adsorbed chemically b) Efficiency decreases with temperature	[163]
6	Zn sheet (99.99%	Alkaline Zn-MnO_2_ batteries	3 and 8 M KOH	Tween 20 and PEG-600	500 ppm (Tween 20) + 500 PEG-600	89.7% (weight loss)	a) Tween and Peg-600 synergistically improve adsorption d) Self-discharge was greatly hindered along with HER	[164]
7	Zn sheet (99.99%	Alkaline Zn-MnO_2_ batteries	ZnO saturated 3 M KOH	PEG-600 + IMZ (imidazole)	0.05 wt.% PEG-600 + 0.05 wt.% IMZ	83% (WL) & 79.9 (PDP)	a) IMZ retards anodic reactions b) PEG-600 reduced cathodic reactions c) Synergistic combination of IMZ and PEG-600 showed better performance than Hg-based inhibitors	[165]
8	Zn sheet	Alkaline Zn-MnO_2_ batteries	ZnO saturated 7 M KOH	DTAB: (dodecyl trimethylammonium bromide)	0.07wt.%	80.2%	a) DTAB acted as an anodic inhibitor b) Delayed Zn passivation c) Forms a fluffy and diffusive protective film, allowing ion diffusion	[166]
9	Zn sheet	Alkaline Zn-MnO_2_ (Zn-C) batteries	26% NH_4_Cl	HEC (Hydroxyethyl cellulose)	300 ppm	93.90%	a) Reduced corrosion rate b) Improved anodic stability c) Improved shelf life and safe operation d) Reduced H_2_ evolution and self-discharge	[157]
10	Zn (98.55%) alloy	Alkaline Zn-MnO_2_ (Zn-C) batteries	7 M KOH	Polyoxyethylene (40) nonylphenyl ether(PNE)	0.25 mM	98.01%	a) Enhanced stability and specific capacity b) Reduced Zn corrosion c) Increased performance and greenness	[167]
11	Zn (bare)	Zn-based alkaline batteries	25 g L^−1^ KOH + 6 M KOH	CMC (carboxymethyl cellulose)	10–20 g L^−1^	43.5%	a) Decreased C_R_, dendrites, and surface roughness d) CMC replaces OH-/H_2_O and self-adsorbs	[168]
12	Zn (bare) powder	Alkaline Zn-Ni secondary batteries	ZnO saturated 6 M KOH	OED (2-octanone ethylene diamine)	4wt.%	95.1%	a) 70% theoretical capacity was maintained for Zn-Ni after 30 cycles b) OED enhances discharge voltage and cyclic capability c) Using N and O, OED forms hydrophobic films	[169]
14	Zn battery grade	Leclanché primary cell (Zn-C)	26% NH_4_Cl	SDBS (Sodium dodecyl benzene sulfonate)	0.8 mM	89.7%	a) Adsorbs at positive potential and forms a protective film b) Smoothened Zn surface c) Outperform HgCl_2_ benchmark	[170]
15	Zn and Zn-Ni alloys	Zn-Ni and Zn-MnO_2_ alkaline batteries	8 M KOH	PEG-400 and CTMAB (cetyltrimethylammonium bromide)	250 ppm PEG + 250 ppm CTMAB	96.8% (Zn-10Ni alloy)	a) PEG-400 and CTMAB synergistically enhance their adsorption b) Reduced self-discharge and anodic dissolution c) Improved corrosion resistance and lifetime	[171]
16	Zinc	Aqueous Zn batteries	1 M ZnSO_4_	AQS (sodium anthraquinone-2- sulfonate)	–	Coulombic efficiency: > 99.6%	a) Self-retention capacity was 92% after 500 cycles b) Showing stable cycling for 470 h	[174]
17	Zn (bare)	Hybrid aqueous batteries	1 M ZnSO_4_ + 1 M LiSO_4_	Pb^2+^ ions (PbSO_4_)	5.0 wt.%	*i*_corr_ reduced 20%	a) Self-discharge retention was nearly 18% b) A 74.4% capacity retention was observed after 300 cycles c) Float charge current reduced to 65%	[177]
18	Zn (bare)	Rechargeable Zn-LiMn_2_O_4_ (aqueous) batteries	2 M ZnSO_4_ + 1 M LiSO_4_	Pyrazole + 5 wt.% fumed silica	0.2wt.% Pyrazole + 5 wt.% fumed silica	*i*_corr_ decreased from 5.25 to 0.9 mA cm^−2^ (~ 83%)	a) Increased recharge capacity b) 83.7% self-retention after 500 cycles d) Self-discharged reduced to 20% after 24 h	[178]
19	Zn metal	Rechargeable Zn-MnO_2_ (aqueous) batteries	2 M ZnSO_4_ with MnSO_4_ and gelatine	Mn^2+^ ions + gelatine	0.2 M (Mn_2_) + ions + 0.5 wt.% gelatine	*i*_corr_ decreased to 75 times	a) Increased specific capacity to 86 mAh g^−1^ b) Increased retention to 79.6% after 1000 cycles c) Gelatine forms a protective film and increases corrosion resistance	[179]
20	Zn (bare)	Rechargeable LiMn_2_O_4_ (aqueous) batteries	2 M ZnSO_4_ + 1 M LiSO_4_	PEG-200, 300, and 400	1 vol% PEG-200	*i*_corr_ reduced from 1.05 to 0.29 mA cm^−2^ (~ 72%)	a) Deposition current density decreased from 8.64 to 3.89 mA cm^−2^ b) Ecorr shifted in the positive direction, retarding H_2_ evolution	[176]
21	Zn (bare)	Alkaline Zn batteries	25 g/L ZnO + 8.5 M KOH	PEG-600 (di acid)	800 ppm PEG-600 di acid	*i*_corr_ decreased from 90 to 10 mA cm^−2^ (9 times)	a) At 65 °C, PEG-600 forms a protective film, increasing the corrosion resistance b) The PEG films also reduced C_R_, water permeability, and HER	[175]
22	Zn (bare) granules	Zn-air flow batteries	7 M KOH	Pluronic F-127 (P127) and sodium Dodecyl Sulfate (SDS)	SDS: 0.2 mM and P127: 100 ppm	*i*_corr_ decreased from 15.54 to 10.51 mA cm^−2^ (SDS)	a) SDS and P127 inhibited passivation and corrosion b) They hinder HER and ZnO film formation c) Charge transfer increases, and current density decreases	[180]
23	Zn-Bi composite	Alkaline Zn-air batteries	6 M KOH	Benzotriazole (BTA), thiourea (CH_4_N_2_S), and SDBS	BTA and SDBS: 60 mg L^−1^ & CH_4_N_2_S: 7.6 g L^−1^	76.9% (BTA) and 69.2% (SDBS)	a) Their %IE followed the sequence: BTA (76.9%) > SDBS (69.2%) > CH_4_N2S (61.5%) b) They form surface protective coordination complexes c) Retention capacity was 96% after 60 cycles	[181]
24	Zn (bare) particles	Alkaline Zn-air batteries	45 wt.% KOH	Tween 20, PEG-600, and SDBS (Sodium dodecyl benzenesulfonate)	5–15 wt.%	Their effectiveness followed the order: SDBS > Tween 20 > PEG-600	a) SDBS, Tween 20, and PEG-600 showed the best performance at 10, 5, and 15 wt.%, respectively b) SDBS forms laminar protective films and improved conductivity c) Suppressed ZnO passivation	[182]
25	Zn (bare)	Zn-air batteries	6 M KOH + additives	Zn(OAc)_2_ (zinc acetate) and Zn(Glu) (zinc gluconate	0.1 M Zn(OAc)_2_ + 0.1 M Zn(Glu)	C_R_ decreased from 0.463 to 0.0467 g m^−2^ h^−1^	a) They adsorb and form a thick film b) They increase the impedance and reduce dissolution c) Dendrite-free surface, longer lifecycle (297 h), and specific capacity (94%)	[183]
26	Zn (bare)	Alkaline Zn-air batteries	0.3 M ZnO + 6 M KOH	PEI (polyethylenimine) MW-600	50 ppm	52.2%	a) PEI adsorbs and reduces dendrites and C_R_ with a slight enhancement of HER b) Surface analyses showed the formation of dense inhibitive films c) Ecorr shifted to the positive side	[184]
27	Zn (bare)	Zn-I_2_ batteries	2 M ZnSO_4_	(Zn_3_(BTC)_2_) MOFs	Ionic Sieve Membrane	C_R_ and H_2_ evolution were reduced	d) C_R_, HER, and dendrite formation were reduced e) Self-retention was 84.6% after 6000 cycles f) Coulombic efficiency was 99.65%	[64]
28	Zn (bare) (99.99%)	Zn-ion batteries	2 M ZnSO_4_	BD (1-butyl-3-methylimidazolium phosphate dibutyl ester salt)	2.5 mM	91.3%	a) Forms stable inhibitive films b) Reduced dendrites and corrosion c) Adsorption follows the LAI model	[185]
29	Zinc (bare)	Zn-ion batteries	1 M ZnSO_4_	EG (ethylene glycol)	25 v/v%	–	a) Decreased corrosion and reduced corrosion product formation	[186]
30	Zinc (bare)	Zn-ion batteries	2 M ZnSO_4_	NH_4_OH	1 mM	Decreased HER and C_R_	a) Lowered HER, C_R_, dendrites, and rough surface b) > 70% capacity retention was observed after 1500 cycles	[173]
31	Zinc (bare)	Zn/Cu, Zn/Zn	2 M Zn(OTf)_2_	scandium (Sc^3+^) ions	0.5 M	99.5% coulombic efficiency	a) Sc^3+^ provides effective cycling, dendrite-free, and very long life, i.e., after 5000 cycles	[187]
32	Zinc (bare)	Zn-MnO_2_ batteries	2 M ZnSO_4_	polyhydroxylated sugar (Trehalose)	100 mM	*i*_corr_ reduced 45%	b) Trehalose chemically adsorbs, altering Zn dissolution c) HER was significantly reduced d) Self-retention capacity was 89% after 1000 cycles and 1600 h	[188]
33	Pure Zn	Zn-air and Zn-ion batteries	3.5% NaCl	Na_2_SO_4_ (sodium sulfate)	0.5 g L^−1^	C_R_ = 4.26 mpy	a) 0.5 g L^−1^increases resistance, and 1.0 g L^−1^ decreases it b) At 0.5 g L^−1^, Na_2_SO_4_ serves as an anodic-type inhibitor and forms ZnSO_4_ film c) Cl- ions integration destabilizes protective films	[204]
34	Zn (bare)	Zn-ion and Zn-V_2_O_5_ batteries	1 M ZnSO_4_	SPS (anionic sodium 3,30-dithiodipropane sulfonate)	10 mM	*i*_corr_ reduced from 23 to 10 mA cm^−2^ (56.5%)	a) SPS adsorbs on the surface and doesn’t produce any effect of Zn^2+^ ions' solvation b) Zn nucleation, HER, and C_R_ were reduced	[203]
35	Zn (bare)	Zn-ion batteries	2 M ZnSO_4_	NTA (nitrilotriacetic acid)	0.15 wt.%	93.9%	a) NTA forms chemisorbed protective films b) C_R_ was reduced 16 times c) Coulombic efficiency 99.4% -99.6%	[205]
36	Zn (bare)	Zn-ion batteries	2 M ZnSO_4_	BHB (6-bromo-1 H-benzimidazole)	2.0 mM	99.1%	a) Forms stable inhibitive films b) Reduced anodic dissolution c) Reduced H_2_ evolution	[206]
37	Pure Zn (99.99%)	Zn-air batteries	6 M KOH	OP-10P (octylphenol polyoxyethylene ether phosphate) and DD (1,10-decanedithiol)	200 mg L^−1^ (OP-10P): 100 mg L^−1^ (DD)	99.9%	a) OP-10P forms the inner layer, and DD forms the outer layer b) Reduced dendrites c) Stabilized discharge	[207]
38	Zn (bare)	Zn-ion batteries	2 M ZnSO_4_	Pyr (pyrazine), Pym (pyrimidine), and Pyd (pyridazine)	6.94 mM	i_corr_ reduced from 0.58 to 0.09 μA cm^−2^ (85%; Pyd)	a) 90% and 78% self-retention after 500 and 3000 cycles, respectively b) Pyd absorbs, and nucleation occurs through N-atom c) Reduced desolvation, H_2_O activities, HER, and byproducts formation	[208]
39	Zn (bare)	Zn-I_2_ batteries	1 M ZnSO_4_	FCD (Fucoidan)	25 mM	Impedance increased from 380 to 776 Ω cm^−2^	a) FCD suppressed dendrites and passivation b) Protective film of FCD shielded electrolyte d) C_R_ was reduced nearly 50%	[155]
40	Zn (bare)	Zn-MnO_2_ batteries	2 M ZnSO_4_	ggg (triglycine)	0.2 mM	Reduced i_corr_ values	a) Ggg coordinates with Zn^2+^ ions, forming a stable film b) Suppressed C_R_ and HER e) Zn life was extended > 4000 h	[209]
41	Zn (bare)	Aqueous Zn-I_2_ batteries	1 M Zn(OAc)_2_	EG (ethylene glycol) + I_2_ (iodine)	10 V% EG + 0.05 wt.% I_2_	C_R_ reduced from 514.7 to 43.7 mpy (15 times)	a) Reduced C_R_ and dendrites b) Self-retention was 91% after 250 cycles and 64.5% after 3000 cycles c) Specific capacity was 1210 mAh g^−1^	[210]
42	Zn (bare)	Zn-V_2_O_5_ batteries	2 M ZnSO_4_	C_5_SeCN (Crown ether)	0.3 wt.%	Impedance rose 3 times	a) C_5_SeCN reduces H_2_O activities, and a Zn–O film was developed b) Coulombic efficiency was 99.4% after 4500 h c) Self-retention was 92% after 300 cycles	[211]
43	Zinc (bare)	Aqueous Zinc batteries	1 M ZnSO_4_	BD (1,2 butanediol) and PD (1,2- pentanediol)	1 vol%	*i*_corr_ reduced from 116.8 to 44.9 μA cm^−2^	a) Zn retention was 98% after 24 h soaking b) BD and PD form an inhibitive film c) Coulombic efficiency was over 99.6%	[212]
44	Zn (bare)	Seawater-based Zn-ion batteries	2 M ZnSO_4_ + 3.5% NaCl	PBTCA (2 phosphonobutane-1,2,4-tricarboxylic acid)	1.0 mM	–	a) Columbic efficiency was over 99.6% after 2000 cycles b) HER reduced to nearly 88% and cycle life rose from 200 to 2000 h d) Protective film retards the diffusion of Cl^−^ and H_2_ evolution	[213]
45	Zn (bare)	Zn-ion batteries	2 M ZnSO_4_	[Zn(AcAc)_2_] (zinc acetylacetonate)	5.6 g L^−1^	*i*_corr_ dropped from 146 to 67 μA cm^−2^	a) Decreased C_R_, wettability, HER, and contact angle b) Self-retention of 52.9% after 12,000 cycles c) Reduced H_2_O activities	[214]
46	Zn (bare)	Zn-ion batteries	2 M ZnSO_4_ + 0.15 MnSO_4_	DA-PPy (dopamine-functionalized polypyrrole)	DA: PPy = 1:10 (20 h)	*E*_corr_ shifted from − 0.023 to − 0.017 (more positive)	a) Decreased H_2_ evolution, charge transfer, and activation energy b) N and O coordinate with the Zn^2+^ ions and form surface protective films	[215]
47	Zn (bare)	Aqueous Zn–S batteries	2 M ZnSO_4_	PCA (pyrrolidone carboxylate)	30–60 mM	*E*_corr_ shifts were observed	a) Inhibits Zn corrosion b) The self-retention was 87% after 30,000 cycles c) Specific capacity was 211 mAh g^−1^	[216]
48	Zn (bare)	Zn-V_2_O_5_ batteries	1 M ZnSO_4_	IP (Imidazo[1,2-b]pyridazine)	0.2 mg L^−1^	*i*_corr_ reduced from 1.293 to 0.368 mA cm^−2^	a) Nearly 70.5% efficiency was derived by IP b) Adsorption of IP anchor Zn^2+^ ions and block H_2_O diffusion c) Coulombic efficiency was 98.72% and highly stable for 2200 h	[217]
49	Zn (bare)	Zn-ion batteries	2 M ZnSO_4_	SG (sodium gluconate)	0.2 M	46%	a) Cycle life increased from 170 to 1800 h b) Attained the specific capacity of 97.4% c) SG forms a protective, highly stable chelating complex	[218]
50	Zn metal	Zn-ion batteries	2 M ZnSO_4_	TBA (2-amino-6- Trifluoromethoxy-benzothiazole)	0.1 mM	99.1%	a) TBA forms a hydrophobic film b) Serves as a mixed-type inhibitor c) Reduced H_2_ evolution and dendrites	[219]
51	Zn (bare)	Aqueous Zn-ion batteries	1 M ZnSO_4_	URT (Urotropine)	5 mM	*i*_corr_ decreases from 0.97 to 0.28 mA cm^−2^	a) Formed bilayer, inhibits HER and dendrites b) Improved wettability, self-life, and self-discharge retention c) Reduced nucleation overpotential	[220]

**Table 5:** A summary of some recent reports on corrosion inhibition of Zn anode using anticorrosive coatings

S/N	Anode material	Battery type	Battery electrolyte	Coating matrix	Coating medium	Efficiency	Salient features	References
1	Zn (bare) particles	Flexible Zn-air batteries	9 M KOH	Al_2_O_3_-based coatings (1.5 wt.%)	via the sol–gel technique	*i*_corr_ reduced from 525.4 to 112.1 μA cm^−2^ (78.7%)	a) Al_2_O_3_ films retards corrosion and H_2_ evolution b) Best performance was observed at 1.5 wt.% after this segregation starts c) Retention capacity was 85% after 7 days	[221]
2	Zn (bare)	Zn-MnO_2_ batteries	3 M Zn(SO_3_CF_3_)_2_	Al_2_O_3_ and ALD-based ultra-thin coatings	Thickness: 0.2 mm	Decreased * i*_corr_ from 8.20 to 4.91 mA cm^−2^ (~ 41%)	a) 89.4% self-retention after 1000 cycles b) Reduced dendrites and contact angles	[222]
3	TiO_2_-coated Zn (TiO_2_@Zn)	Zn-MnO_2_ batteries	Aqueous 3 M Zn(OTf)_2_	Ultrathin TiO_2_ coating (ALD)	Thickness: 8 nm	H_2_ evolution was slowed	a) Reduced HER and formed Zn(OH)_2_ passive film b) Increased self-discharge stability c) Self-retention was 85% after 1000 cycles d) 99% coulombic efficiency was achieved under long-term exposure	[223]
4	anti-corrosion elastic constraint (AEC)-Zn	Zn-MnO_2_ batteries	2 M ZnSO_4_	TiO_2_ NPs + PVDF coatings	Thickness: 5 μm	Polarization decreased > 60%	a) Coulombic capacity was 99.4% b) PVDF blocks H_2_O/O_2_ diffusion c) Stable up to 2000 h cycling	[224]
5	Zn particles	Zn-air batteries	6 M KOH	SiO_2_-based coatings	CVD and CSD coatings	~ 40% reduced H_2_ evolution	a) Reduced Zn dissolution and H_2_ evolution b) Semiconductive Zn_2_SiO_4_ coatings were derived from CVD c) Insulating SiO_2_ coatings were derived from CSD d) Coatings improved oxidation stability	[225]
6	Zn coated with ZrO_2_	Zn-MnO_2_ batteries	2 M ZnSO_4_	ZrO_2_-based coatings	Thickness: 4 μm	C_R_ reduced 3 times	a) Coulombic efficiency of 99.36% was derived b) C_R_ and HER were reduced c) Stable up to 3800 h cycling	[226]
7	Zn (bare) with CeO_2_	Zn-ion batteries	2 M ZnSO_4_	CeO_2_-based non-conductive coatings	Thickness: 10 μm	i_corr_ decreased from 1.906 to 0.955 mA cm^−2^	a) Coating decreased the contact angle (42.4°) and increased the wettability b) Very low polarization decreased gas evolution and surface bubbling c) Retention capacity was 98.5% after 4000 cycles	[227]
8	Zn with	Aqueous Zn batteries	2 M ZnSO_4_	ZnNb_2_O_6_^−^basec coatings	Thickness: 5 μm	C_R_ decreased nearly 16%	a) Cycle life of > 2000 h was attained after 3000 cycles b) Coulombic efficiency was 99.54% to 99.99%, with a retention capacity of 80.2% after 3000 cycles c) ZnNb_2_O_6_ serves as a multifunctional artificial solid electrolyte interphase (SEI)	[228]
9	Zn (bare)	Zn-MnO_2_ batteries	2 M ZnSO_4_	Al_2_Si_2_O_5_(OH)_4_ (Kaolin coating)	Thickness: 18–27 μm	Impedance increased 2 times	a) Excellent anticorrosion property b) Dendrite-free surface c) Long cycle stability, i.e., 800 h and 600 cycles	[229]
10	Zn particles	Zn-air batteries	7 M KOH	PANI coatings (20PANI@Zn)	20 mL HCl	85%	a) Reduced self-discharge b) Reduced HER c) 97.8% retention capacity after 24 h	[230]
11	Zn@C core shells	Rechargeable Zn-MnO_2_ batteries	0.125 M ZnO + 6 M KOH	Carbon (C) shell coatings	Electrochemical reduction method (ZnO@C-A)	ZnO leaching decreased from 12.5 to 2.5%	a) 98.8% retention capacity was observed after 400 cycles b) Coulombic efficiency was 84% after 400 cycles c) ZnO leaching decreased from 12.5% to 2.5%	[231]
12	Zn (bare)	Zn-MnO_2_ batteries	2 M ZnSO_4_	sCNT-based coatings	0.0125 wt.% sCNT; Thickness: 15 μm	i_corr_ reduced from 2.16 to 0.52 mA cm^−2^	a) Reduced C_R_ and HER and dendrites free Zn deposition b) High self-discharge stability up to 1600 h c) Coulombic efficiency was 99.4%	[232]
13	Zn (bare)	Zn-ion batteries	2 M ZnSO_4_	Cellulose nanofillers (CNF) + graphene acid (GA) coatings	Thickness: 30 μm (0.5 wt.% GA + 2 wt.% CNF	i_corr_ reduced from 1.74 to 0.25 mA cm^−2^ (85.6%)	a) 99% retention after 24 h b) The -COOH of GA helps in desolvation through adsorption d) Increased mechanical strength, reduced HER, and E_a_	[233]
14	Zn (bare)	Zn-ion batteries	3 M ZnSO_4_	Cellulose-based coatings	Thickness: 5 μm	C_R_ decreased by 62%	a) Overpotential decreased from 220 to 65 mV b) Self-life increased to 500 h from 57 h c) Attained a specific capacity of 89.4%	[234]
15	Zn coated with Cu–Zn (Zn-Cu/Zn)	Rechargeable Zn batteries	3 M ZnSO_4_	Zn-Cu-based coatings	Thickness: 20.1 ± 0.9 μm	C_R_ was reduced 6 times 86% efficiency	a) Coulombic efficiency increased to 91.8% and HER reduced to nearly 80% b) Cu-coating decreased Zn oxidation and dissolution c) Long cycle life even after 1500 cycles	[235]
16	Zn (bare)	Aqueous Zn-MnO_2_ batteries	2 M ZnSO_4_ + 0.1 M MnSO_4_ and 6 M KOH (ZnO)	InCl_3_ (indium chloride)-based coating;	Ion exchange (150 mM; thickness: 8 μm)	Decreases i_corr_ to 7.7 mA cm^−2^	a) Suppressed HER and corrosion rate b) Decreased current and dendrites c) Long-life and 72% retention capacity	[236]

**Table 6:** A summary of some recent reports on corrosion inhibition of Zn anode using Zn anode alloying

S/N	Anode alloy	Battery type	Battery Electrolyte	Alloying element and ratio	Efficiency	Salient features	Refs
1	Zn powder	Alkaline Zn-air batteries	7 M KOH + 2 wt.% PAA	3 wt.% Bi and Ni	i_corr_ reduced from 1.2718 to 0.8490 mA cm^−2^	a) Bi improves efficiency and reduces HER (4 times) b) E_corr_ slightly shifted in the positive direction c) Bi inclusion delays spontaneous corrosion	[240]
2	Zn powder	Alkaline Zn-air batteries	7 M KOH + 2 wt.% PAA	5 wt.% Bi and 5 wt.% Ni	R_ct_ increased from 55.4 to 602.2 Ω (ZnBi-2) (90.8%)	a) Bi decreased H_2_ overpotential and shifted E_corr_ in a slightly negative direction b) Ni mostly helped in the reduction of H_2_ evolution c) Bi and Ni improve the surface morphology	[241]
3	Zn-Bi (98:2) alloy	Zn-air batteries	7 M KOH + 2 wt.% PPA	2 wt.% Bi	91.1%	a) The lowest current density (0.326 mA cm^−2^) was derived from Zn-Bi alloy b) 99.5% discharge capacity was retained c) KOH-PAA reduced self-discharge	[242]
4	Zn powder	Alkaline Zn-air batteries	6 M KOH	1.5 wt.% Bi	53.8%	a) Specific capacity and energy density increased to 21.7% and 28.7%, respectively b) 84.6% capacity retention was observed after 60 cycles c) Bi reduced dendrites and H_2_ evolution and overpotential	[243]
5	Zn-Bi alloy	Zn-ion batteries	1 M ZnSO_4_	Bi	i_corr_ reduced from 3.589 to 0.333 mA cm^−2^	d) Nearly 90% reduction in i_corr_ was observed, and HER overpotential raised to -1.81 from 1.72 V e) Coulombic efficiency was 99.6% after 1000 cycles f) No dendrite formation occurs	[244]
6	Zn and Zn-Sn alloy	Zn-ion batteries	2 M ZnSO_4_	Sn	31%	g) Sn reduced C_R_ and HER h) Sn increased the lifecycle by 150% as compared to pure Zn i) Zn-Sn manifests a heat capacity of 89.5% after 1000 cycles	[245]

### Electrochemical Corrosion and Corrosion Inhibition of Sustainable Aluminum-Based Batteries

**Fig. 16:**
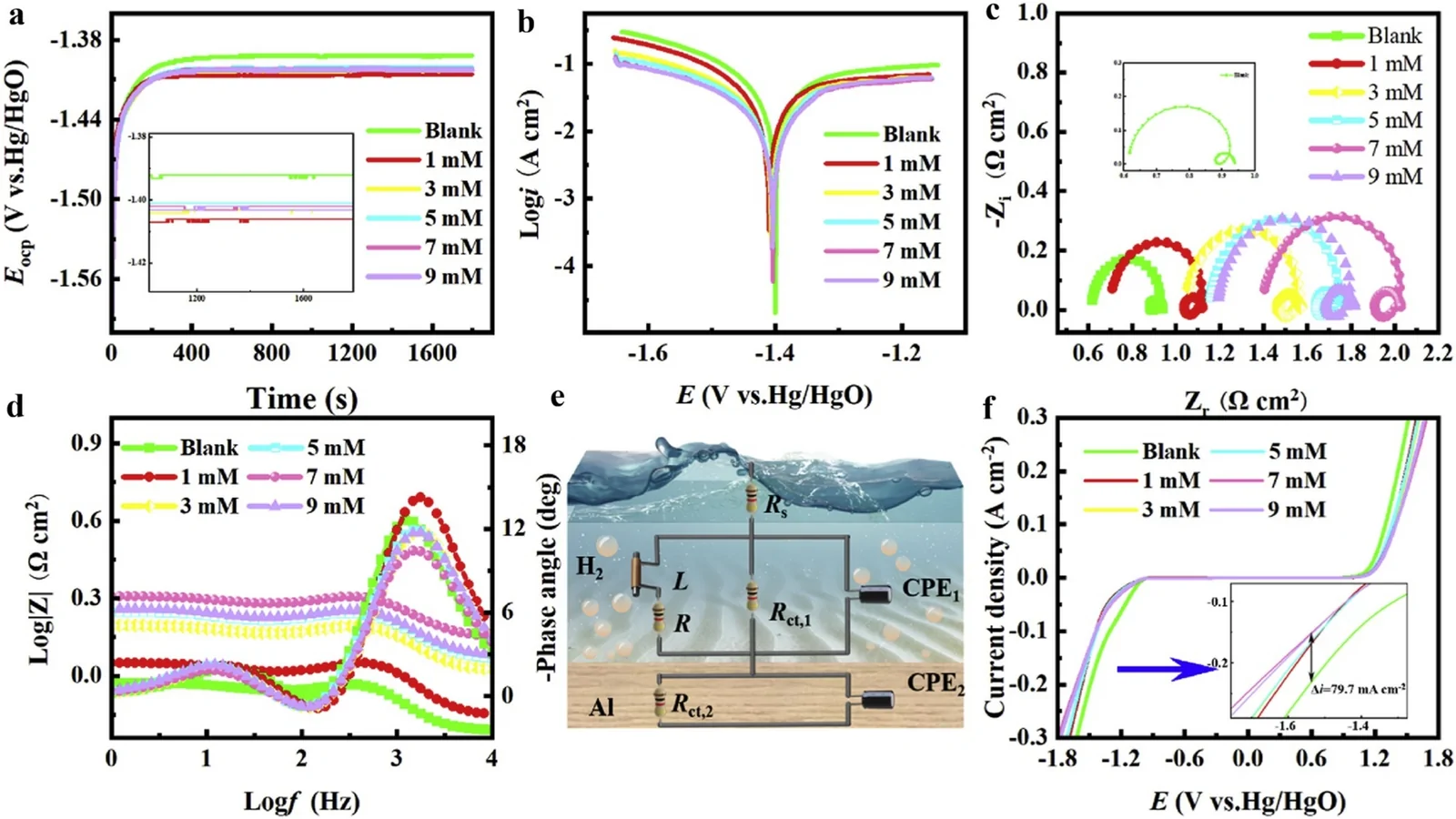
Al-5052 anode's **a** EOCP-time curves and **b** PDP curves in 4 M NaOH with and without FDLH. Nyquist and Bode graphs of Al-5052 in 4 M NaOH with and without FDLH are shown in **c** and **d**, respectively. **e** Equivalent in correspondence. **f** Window for electrochemical stabilization using various electrolytes [252] (Reproduced with permission. © 2025 Elsevier)

Al-air batteries have attracted significant attention in energy storage systems owing to their low cost, high theoretical energy density, and eco-friendly nature [246, 247]. Nevertheless, like Zn-based batteries, the practical implementation of Al anode in electrode in alkaline electrolytes, mainly KOH and NaOH solutions, is constrained by its susceptibility toward corrosion [248, 249]. Al reacts with H_2_O and OH^−^ and forms Al(OH)_3_ and H_2_ [250]. Corrosion leads to continuous, undesirable aluminum (Al) loss and hydrogen (H_2_) evolution, resulting in poor anode utilization and coulombic efficiency. Al(OH)_3_ layers serve as a barrier for discharge uniformity and electron transfer, resulting in a volatile voltage profile and reduced lifespan [251]. These circumstances increase *C*_R_, *i*_corr_, and H_2_ evolution and enhance the probability of pitting corrosion. Recently, the use of organic compounds has emerged as an effective inhibitor for these undesirable reactions. Huo et al. studied and documented the inhibition potential of an amino acid derivative, namely N-α-Fmoc-N-epsilon-Boc-D-lysine (FDLH), for Al-5052 anode in 4M NaOH electrolyte [252]. They observed that FDLH significantly reduced AL-5052 anode dissolution and H_2_ evolution. A careful inspection of Fig. 16 reveals that the electrochemical behavior is dramatically altered in the presence of FDLH. EOCP vs. time curves shifted to the positive (anodic) side, indicating the formation of protective films of FDLH.

**Fig. 17:**
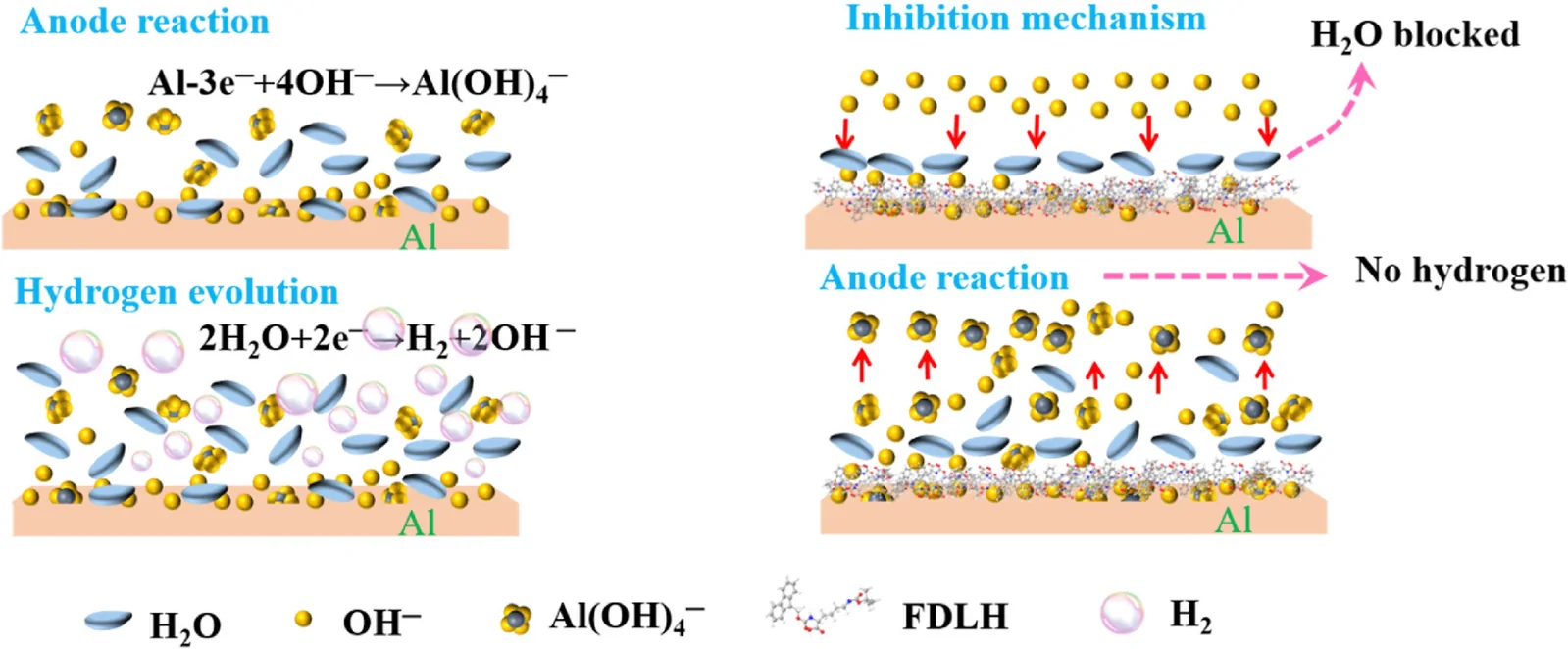
Schematic illustration of the FDLH corrosion inhibition process [252] (Reproduced with permission. © 2025 Elsevier)

PDP curves also showed a remarkable shift and reduction in icorr in the presence of FDLH. The tested inhibitor demonstrated 57.1% efficiency in inhibiting Al anode dissolution, serving as a mixed-type inhibitor. The increased diameter of the Nyquist plot and the enhanced impedance and phase angle of the Bode plot in the presence of FDLH suggest increased passivation and a higher *R*_ct_ for Al corrosion. EIS data were fitted using an equivalent circuit. In the absence of FDLH, the Al anode is freely attacked and corroded by H_2_O and OH^−^, leading to unrestricted corrosion and dissolution. However, FDLH forms a stable protective film using Al–O–C and Al-O-N bonds (Fig. 17). The dense, uniform, and hydrophobic film exhibits multifunctional properties, enhancing both corrosion resistance and battery performance. FDLH inhibits both anodic and cathodic reactions. These observations showed that the FDLH inner layer serves as a chemical passivator while its outer layer retards the diffusion of corrosive species, including OH^−^ and H_2_O. The inhibition potential of organic and polymeric inhibitors, including PVA, CTAB, heterocycles, sodium octanoate (SO), sodium benzoate (SB), and sodium citrate hydrate (SC), has been studied and described widely [253–258]. They effectively adsorb at the interface between the Al anode and the alkaline electrolyte via their electron-rich coordination sites, thereby inhibiting corrosion.

**Fig. 18:**
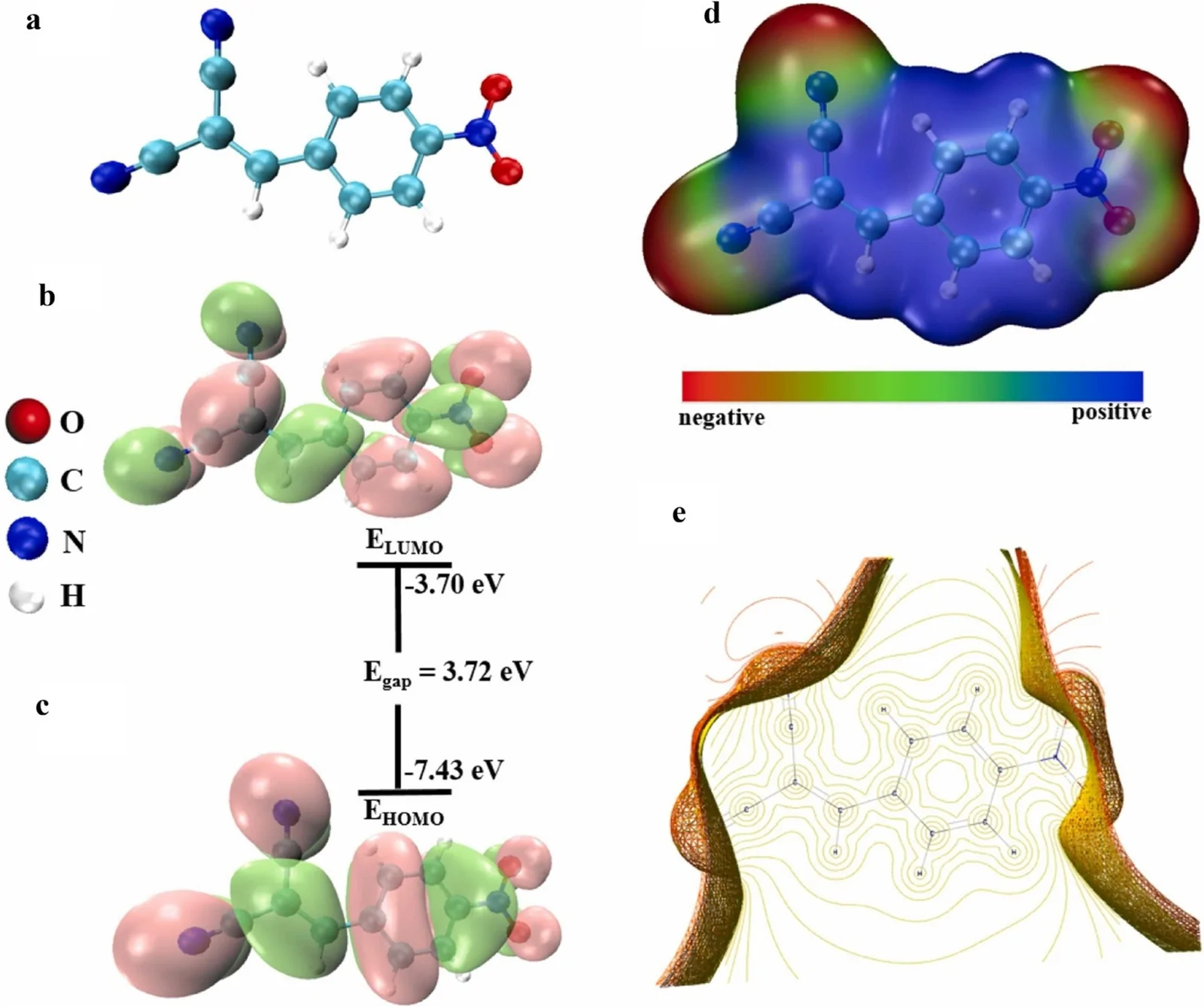
Electronic structure analysis of the Z4 monomer displaying: **a** optimized geometry, and **b** orbital distributions of LUMO and **c** HOMO. **d** ESP and **e** MESP visualization [254] (Reproduced with permission. © 2025 Elsevier)

El-Alouani et al. showed that the electronic structures of inhibitors played a crucial role in their adsorption and inhibition potential [254]. They showed that HOMO and LUMO were distributed over –NO_2_ and –CN groups of Z4 molecules, indicating that these sites were involved in the charge transfer and adsorption (Fig. 18). The careful inspection also reveals that electron density regions are mainly located around O and N atoms, suggesting that they serve as sites for coordination bonding and chemisorption. These molecules retard the self-corrosion of the Al anode and form protective films. Many studies reported the inhibition potential of tannic acid (TA) [259], [Amim][TFSI] (ILs) [260], ZnO + PEG diacid [261], nonoxynol-9 (a non-ionic surfactant) [262], and CTAB [263]. They adsorb on the Al surface using their polar functional groups, such as –OH, –NH_2_, –SH, and –COOH, as well as the π-electrons of aromatic systems or side chains. Their adsorption results in the retardation of anodic and cathodic reactions. Protein- and biopolymer-based inhibitors have emerged as promising, effective, and eco-friendly alternatives to aluminum corrosion inhibition. These green molecules, including L-cysteine, alkyl polyglucosides (APG), L-aspartic acid (Asp), fulvic and humic acids, and casein protein, are associated with numerous polar functional groups that coordinate with the Al surface and form a chelating complex to inhibit corrosion [264–269]. They achieve more than 80% efficiency by developing corrosion-protective inhibitive films.

Surfactants and ILs have also demonstrated outstanding potential for inhibiting Al anode corrosion in alkaline electrolytes by altering the electrochemical potential, retarding self-corrosion, and inhibiting H_2_ evolution. For the development of corrosion-protective films, the heteroatoms (N, O, and S) of ILs coordinate with the Al surface, demonstrating more than 96% efficiency [260]. Nonoxynol-9 and quaternary ammonium surfactants manifest reasonably high efficiency and serve as mixed-type corrosion inhibitors [262, 263]. Some studies also documented the synergistic effects of organic–inorganic hybrid additives for Al anode corrosion protection in battery environments. Their synergistic formulations are better inhibitors than their isolated counterparts. For example, the L-cysteine/ZnO hybrid manifests excellent protection for Al dissolution [270]. The authors observed and reported that cysteine coordinates on the Al surface, forming Al–Zn–S and Al–Zn–O, and significantly inhibit corrosion. Likewise, the 8-HQ /ZnO hybrid forms Al-O and Zn-N type complexes and inhibits corrosion and retard H_2_ evolution [271]. The literature survey on composite materials on Al anode corrosion inhibition shows that NiAl-vanadate(LDH/PVA) hybrid [272], PGA + K_2_SnO_3_ [267], and decyl glucoside + decanedithiol + K_2_SnO_3_ [273] have been effectively used.

**Fig. 19:**
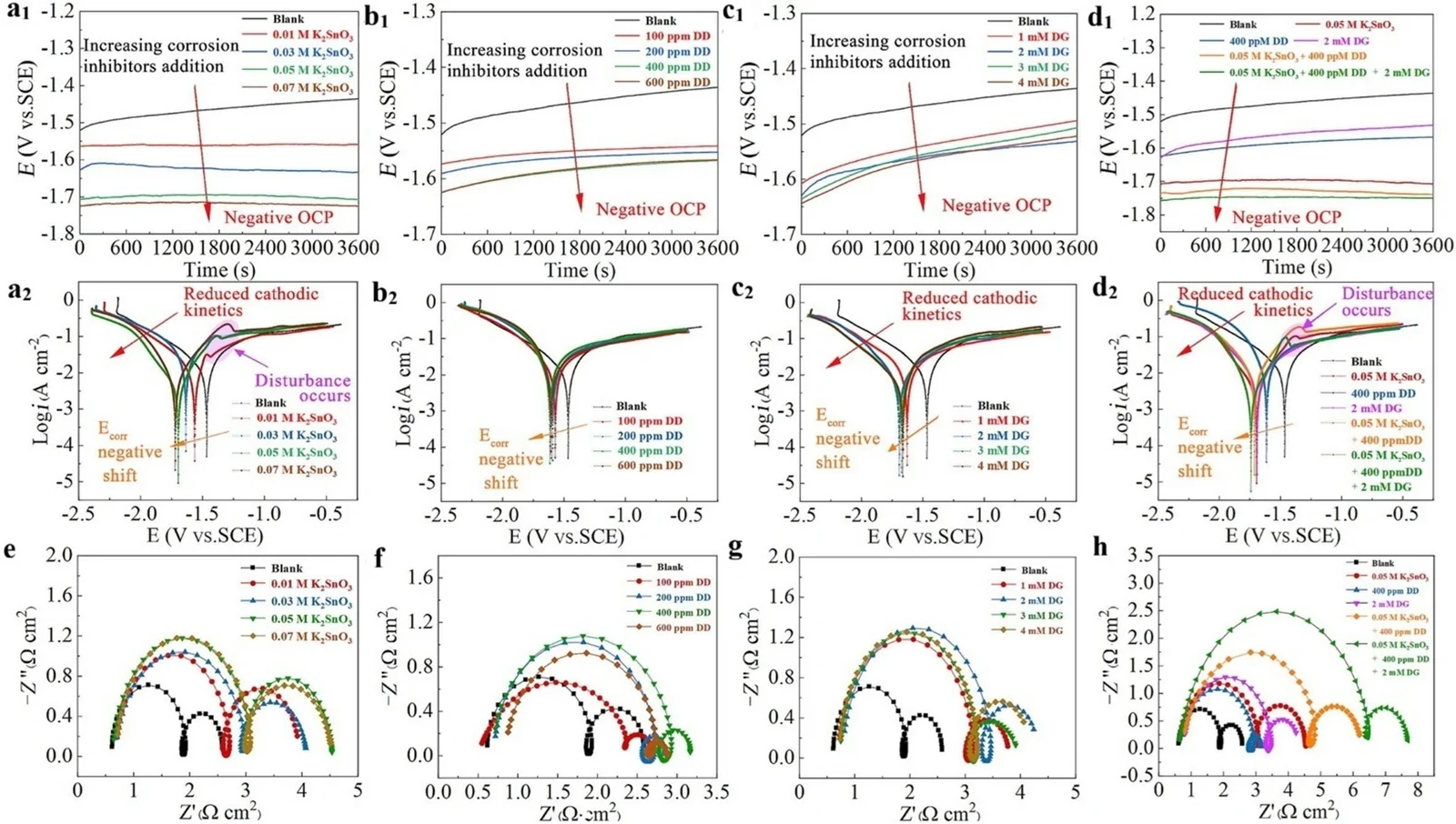
E_OCP_ versus time curves and dynamic polarization curves for the Al-1080 anode in 4 M KOH with and without additives **a** K_2_SnO_3_, **b** DG, **c** DD, and **d** summary of K_2_SnO_3_, DD, DG, K_2_SnO_3_ + DD, and K_2_SnO_3_ + DD + DG. Electrochemical impedance spectra of the Al-1080 anode in 4 M KOH with and without additives and corresponding equivalent circuits **e** K_2_SnO_3_, **f** DD, **g** DG, and **h** summary of K_2_SnO_3_, DD, DG, K_2_SnO_3_ + DD, and K_2_SnO_3_ + DD + DG [273] (Reproduced with permission. © 2025 Elsevier)

**Fig. 20:**
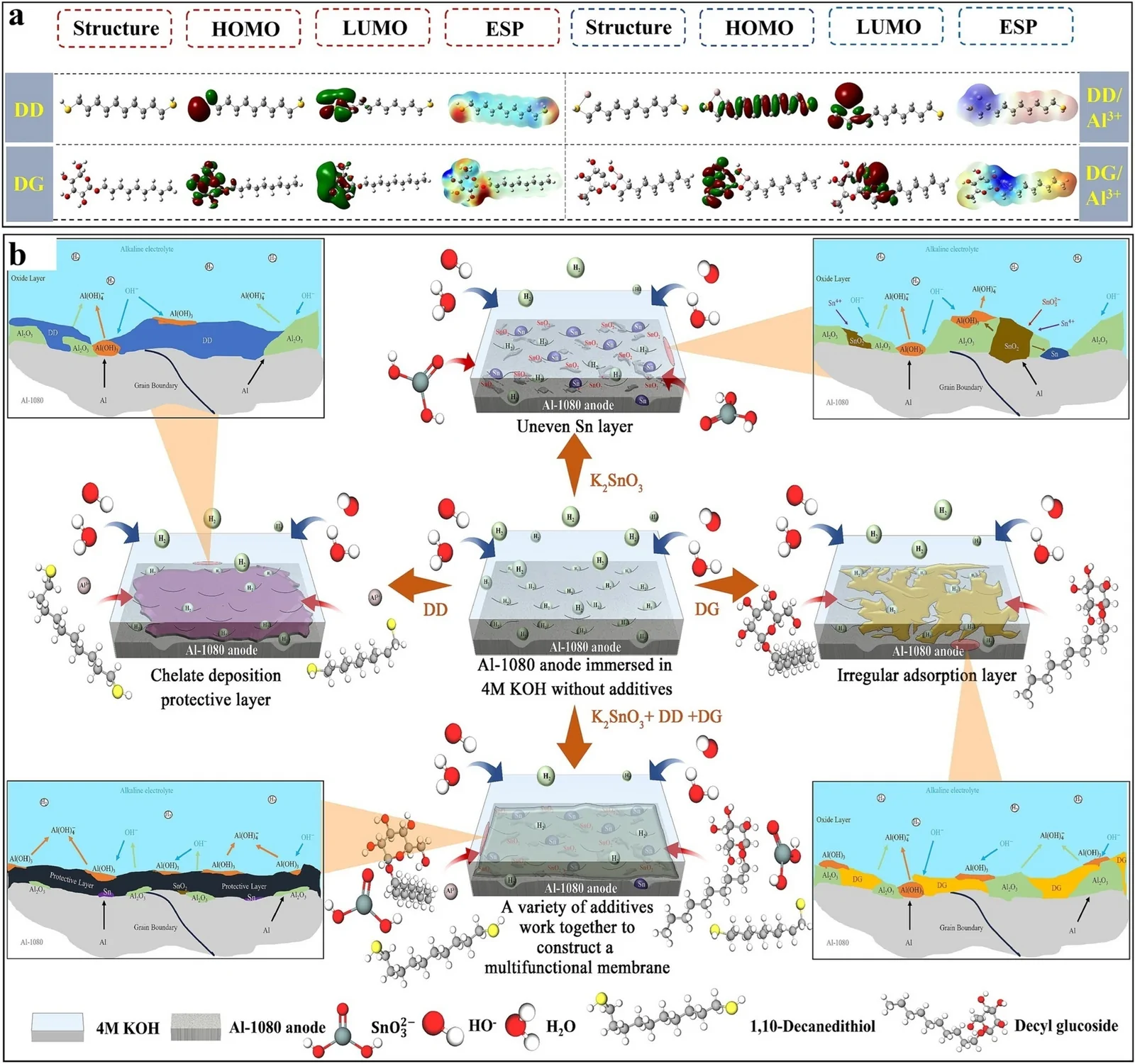
DFT computation and the mechanism of corrosion inhibition. **a** Diagrammatic representation of the Al-1080 anode's corrosion inhibition process in 4 M KOH, both with and without additives. **b** LUMO, HOMO, and ESP structures optimized for DD, DG, DD/Al^3+^, and DG/Al^3+^ [273] (Reproduced with permission. © 2025 Elsevier)

Zhang et al. studied the synergistic performance of decyl glucoside (DG), decanedithiol (DD), and K_2_SnO_3_ for Al-1080 corrosion in 4 M KOH [273]. The PDP and OCP analyses revealed that the presence of DG, DD, and K_2_SnO_3_ shifts *E*_corr_ toward the positive direction, indicating control over H_2_ evolution kinetics. EIS analyses validate the PDP results, showing that the Nyquist plots for a synergistic combination of DD, DG, and K_2_SnO_3_ exhibit the largest semicircle diameter (Fig. 19). This finding suggests that DD + DG + K_2_SnO_3_ exhibits the highest resistance and the lowest corrosion rate. The presence of different components in the formulation enhances their adsorption and film-forming ability, which is responsible for reduced *C*_R_, *i*_corr_, and H_2_ evolution, as well as increased R_ct_ values. Figure 20 shows the corrosion inhibition mode of the DD + DG + K_2_SnO_3_ system. SnO_3_^2−^ ions are first released from K_2_SnO_3_ and form a Sn-based protective film through their deposition. The adsorption of DG follows this step to enhance the hydrophobic character of the protective films and fill the surface defects and cracks. Lastly, DD coordinates with Al and adsorbed Sn through S atoms, forming Al-S and Sn-S bonds to increase the protection of existing films and their density. More so, DFT-based analyses showed that DG is associated with a smaller Δ*E* (*E*_LUMO_ − *E*_HOMO_) and a higher dipole moment than DD. This results in a higher binding affinity of DG for Al^3+^ ions than for DD. Nevertheless, the DD + DG + K_2_SnO_3_ system develops Al-Sn-S-C-O-based multifunctional layers to retard anodic dissolution and cathodic H_2_ evolution.

**Fig. 21:**
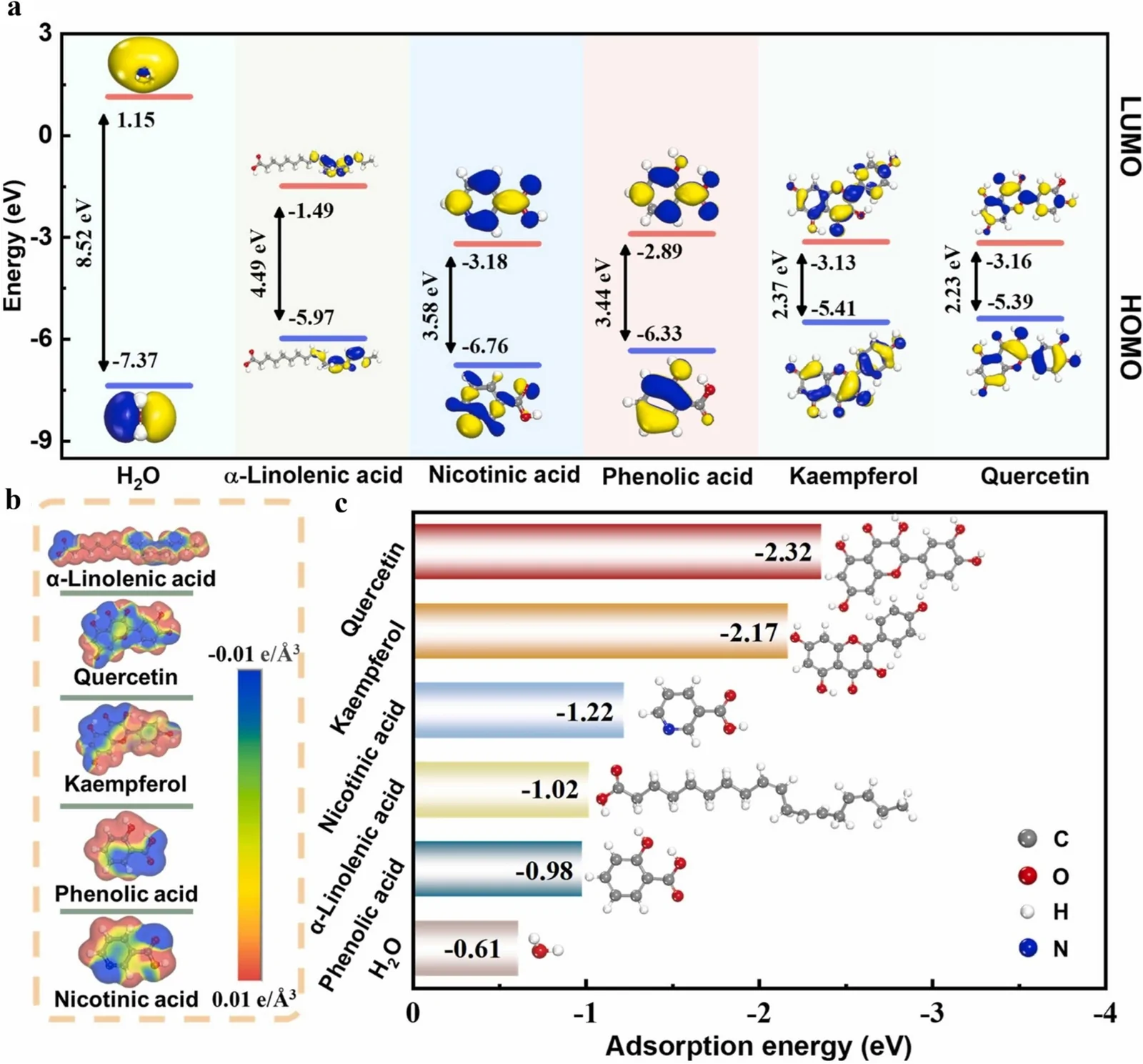
Schematics showing **a** FOMs of H_2_O and different active constituents of TSE extract; **b** EPM of TSE constituents; and **c** E_ads_ of TSE constituents on the Al surface [274] (Reproduced with permission. © 2025 Elsevier)

Bio-based QDs and plant extracts have emerged as carbon-rich and green corrosion inhibitors for the Al anode in alkaline batteries [259, 274–276]. Gao and coworkers studied the inhibition efficiency of Toona sensis extract (TSE) containing different active phytochemicals for Al anodic dissolution [274]. They observed that the active constituents bind to the Al surface, forming O–Al and N–Al bonds and thereby forming inhibitive films. Figure 21 presents the distributions of the HOMO, LUMO, and FMOs, their energy gaps, electrostatic potential maps (EPMs), and adsorption energies for different active phytochemicals. The authors observed that kaempferol and quercetin were associated with the lowest energy gap, highest chemical reactivity, and the best coordination ability. EPMs were mainly located around N and O atoms, indicating they form Al–O and Al–N complexes. Careful observation shows that quercetin had the most negative *E*_ads_ value, followed by kaempferol and nicotinic acid. Based on different outcomes, they proposed that different constituents adsorbed on the Al surface via chemisorption. Polymer-based protective layer including PVA [253], NiAl-vanadate-based LDH/PVA [272], and Prussian blue integrated PAA [277] also manifested reasonable Al protection in alkaline electrolytes. Chung et al. showed that alkali metal cations such as Rb^+^, K^+^, Na^+^, and Li^+^ affect the discharge and corrosion properties of Al in KOH-based electrolyte [278]. The outcomes suggest that Rb^+^ showed the best corrosion resistance and discharge capacity, validating that large cations provide superior electrochemical stability. Other reports also documented the inhibition of the Al anode in battery electrolytes [254, 256, 257, 279, 280].

The alloying approach for increasing the corrosion resistance of the Al anode in battery and saline environments has mainly been explored. Minor alloying elements, including Bi, Mg, Zn, Sn, In, and Ga, significantly enhance the corrosion resistance and anodic activities [281–284]. Different alloying elements perform various functions. For example, Sn decreases H_2_ evolution and breaks oxide films, thereby activating Al. Mg decreases H_2_ evolution, while Zn stabilizes dissolution and raises the H_2_ evolution overpotential. Bi and In disrupt oxide layers, and Cu forms protective films (passivation). Additionally, Ga controls surface activities. Summaries of some representative results showing the effects of corrosion inhibitors and alloying are presented in Tables 7 and 8.

**Table 7:** A summary of some recent reports on corrosion inhibition of Al anode using corrosion inhibitors

S/N	Anode material	Battery type	Battery electrolyte	Corrosion inhibitor(s)	Inhibition properties		Salient features	References
					Conce	Efficiency		
1	Al-5052 alloy	Alkaline Al-air batteries	4M NaOH	FDLH (N-α-Fmoc-N-epsilon-Boc-D-lysine)	7 mM	60.9%	a) FDLH adsorbs on the Al surface and coordinates by using the -COOH’s oxygen Protective films contain Al(OH)_3_ and Al_2_O_3_	[252]
2	Pure Al (99.99%)	Alkaline Al-air batteries	4M NaOH	PVA (polyvinyl alcohol)	1% (PVA; MW1099)	60.44%	b) -OH helps in adsorption and corrosion inhibition c) A 50% to 60% reduction in H_2_ evolution was observed Reduces *i*_corr_, C_R_, and HER through a double-fold adsorption process	[253]
3	Al-5052 alloys (AlMgCr)	Alkaline Al-air batteries	4M NaOH	Z4 (2-n(4-nitrobenzylidene) malononitrile)	1 × 10^–3^ M	*i*_corr_ reduced from 29.0 to 19.6 mA cm^−2^ (~ 32.4%)	d) Reduced C_R_, *i*_corr_, and H_2_ evolution e) Serves as a mixed-type inhibitor, and adsorption occurs through N and O Energy density improved by 2.5 times	[254]
4	Pure Al (99.99%)	Alkaline Al-air batteries	4M NaOH	MPA (2-mercapto-3-pyridinecarboxylic acid)	10 mM	*i*_corr_ reduced from 25.37 to 13.68 mA cm^−2^ (46.1%)	a) –SH–, pyridine N, and –COOH serve as adsorption centers, forming Al-O/S/N bonds b) Discharge lifetimes increase 1.5 times f) Specific capacity increased 65.7%	[255]
5	Al-5052 alloys (AlMgCr)	Alkaline Al-air batteries	4M NaOH	CPPE (cashew phenol polyoxyethylene ether)	0.2 mM	74.5%	g) CPPE adsorbs using the oxygen of -OH and ether groups to form Al-O h) Hydrophobic layer improves surface passivation CPPE films reduce C_R_ and HER	[256]
6	Al-5052 alloys (AlMgCr)	Alkaline Al-air batteries	4M NaOH	Hybrid inhibitor (8-hydroxyquinoline-5-sulfonic acid (H_2_QS) and CTAB)	0.6 mM (CTAM) + 0.5 mM (H2QS)	94.19%	c) Increased R_ct_ and decreased *i*_corr_ values d) O and N coordinate and form chelating films Increase cycling stability	[257]
7	Pure Al (99.99%)	Alkaline Al-air batteries	1M NaOH	SO (sodium octanoate)	10 wt.%	*i*_corr_ reduced from 32,000 to 16,800 μA cm^−2^ (47.4%)	e) Strongest binding energy (E_bin_: 379 kcal mol^−1^) f) Highest activity (ΔE = 3.577 eV) g) Form a stable chelating complex through chemisorption	[258]
8	Pure Al (99.99%)	Alkaline Al-air batteries	1M NaOH	SB (sodium benzoate)	5 wt.%	34.8%	h) The π-electrons and -COOH work together and form physicochemisorption i) Stronger binding energy (E_bin_: 135 kcal/mol) j) Higher activity (ΔE = 3.708 eV)	[258]
9	Pure Al (99.99%)	Alkaline Al-air batteries	1M NaOH	SC (sodium citrate hydrate)	5 wt.%	36.6%	k) Multidentate adsorption l) Stronger binding energy (E_bin_: 307 kcal mol^−1^)	[258]
10	Pure Al (99.99%)	Neutral Al batteries	5M NaCl solution + Na_2_S_2_O_8_ (catholyte)	TA (tannic acid) green corrosion inhibitor	400 mg	i_corr_ reduced 0.0027 μA cm^−2^	m) Suppressed C_R_, pitting corrosion, i_corr_, and dendrites n) Increase specific capacity, energy density, and recycling o) TA form Al-O-C coordinate bond through adsorption	[259]
11	Pure Al (99.99%)	Alkaline Al-air batteries	4M NaOH	Ionic liquids (([Amim][TFSI]))	1.5 mM	96.2%	a) IL predominantly serves as a mixed-type inhibitor b) Self-corrosion and H_2_ evolution were reduced p) Improved operating voltage, energy density, and anodic efficiency	[260]
12	Pure Al (99.99%)	Alkaline Al-air batteries	4M NaOH	PEG diacid + ZnO	5000 ppm PEG diacid + 16 g L^−1^ ZnO	C_R_ was reduced 10 times	a) PEG diacid suppressed anodic dissolution and cathodic H_2_ evolution c) Zn/PEG diacid film develops during the reaction	[261]
13	Pure Al (99.99%)	Alkaline Al-air batteries	4M NaOH	N9 (Nonoxynol-9)	2.0 mM	92.8%	d) Decreased i_corr_, C_R_, and H_2_ evolution e) Adsorption followed the Freundlich adsorption model b) Improved discharge voltage, anode utilization, and energy capacity	[262]
14	Pure Al (99.99%)	Alkaline Al-air batteries	4M NaOH	CTAB, DDAB, and DDBAB	0.6 mM	i_corr_ reduced from 22.1 to 10.4 mA cm^−2^	f) All surfactants serve as cathodic-type inhibitors g) Their effectiveness followed the sequence: DDBAB > DDAB > CTAB h) Discharge voltage and capacity density were increased	[263]
15	Pure Al (99.99%)	Alkaline Al-air batteries	5M NaOH	6-Thioguanine	0.5 mM	*i*_corr_ reduced from 9.725 to 61.70 mA cm^−2^ (~ 36.56%)	c) Decreased *i*_corr_, C_R_, and H_2_ evolution. Served as a mixed-type inhibitor d) High concentration reduced efficiency i) –C = N–, –SH and –NH_2_ serve as adsorption sites for adsorption of inhibitors	[264]
16	Al alloy (Mg-Mg-Ca-Zn-Sn)	Alkaline Al-air fuel cell	4M NaOH	Casein protein and Na_2_SnO_3_	0.6 g L^−1^ Casein + 0.05 M Na_2_SnO_3_	83.1%	j) Casein-mediated uniform deposition of Sn occurs, forming inhibitive films e) The amide and carbonyl groups of casein coordinate with Al^3+^ and Sn^2+^	[265]
17	AA-5052 alloys	Alkaline Al-air batteries	4M NaOH	L-aspartic acid (Asp) and CaO	10 mM Asp and 4 mM CaO	*i*_corr_ reduced from 87.84 to 22.38 mA cm^−2^ (~ 74.5%)	k) Ca(OH)_2_ forms, which provide coverage and corrosion protection l) Corrosion was reduced, while the reactivity of the anode remained unchanged m) Discharge voltage and capacity density were improved	[266]
18	Al alloy (Al–Mg-Ga-Sn-Zn)	Alkaline Al-air batteries	4M NaOH	Alkyl polyglucoside (APG) and K_2_SnO_3_	2 mM APG and 0.05 M K_2_SnO_3_	H2 evolution reduced from 0.735 to 0.043 mL cm^−2^ min^−1^ (~ 94.14%)	n) APG boosts uniformity and protectiveness of Sn film o) SnO_3_^2−^ and APG work together synergistically p) Discharge voltage, capacity, and anode utilization were improved	[267]
19	Pure Al (99.99%)	Alkaline Al-air batteries	5M NaOH	humic/fulvic acid mixture (Flax straw extract)	3 vol%	*i*_corr_ reduced nearly 50%	f) C_R_, *i*_corr_, H_2_, and current were reduced, and they serve as mixed-type inhibitors g) R_p_, discharge voltage, and capacity, and overall efficiency increased	[268]
20	Pure Al (99.9%)	Alkaline Al-air batteries	4M NaOH	Hybrid additives (agar + L-cysteine + ZnCl_2_)	0.25 wt.% agar + 10 mM Cys + 10 mM ZnCl_2_	HER reduced from 1.47 to 0.0833 mL cm^−2^ min^−1^	i) Agar adsorbs and forms a uniform protective layer j) ZnCl_2_ provides a blocking effect, avoids Zn dissolution, and retards H_2_ evolution h) Using –NH_2_ and –COOH groups, Cys links Al(OH)_3_ and Zn^2+^	[269]
21	Pure Al (99.99%)	Alkaline Al-air batteries	4M NaOH	Hybrid inhibitor (NiAl-vanadate LDH + Poly(vinyl acetal)	LDH: PVA = 85:15	i_corr_ reduced from 32.35 to 6.16 mA cm^−2^	a) Reduced C_R_, *i*_corr_, and H_2_ evolution b) Two-fold protection as LDH provides Al^3+^/OH^−^ channels and PVA forms a hydrophobic film to retard H_2_O diffusion q) Specific capacity, energy density, and power density were increased	[272]
22	Commercial Al-1060	Alkaline Al-air batteries	4M NaOH	L-cysteine + ZnO	0.03 M (L-cysteine) + ZnO (0.2 M)	*i*_corr_ reduced from 47.73 to 6.74 mA cm^−2^ (85%)	c) Reduced self-corrosion, H_2_ evolution, and Zn^2+^ diffusion d) Dense Al-Zn-C-S films are formed r) Cys serve as multidentate ligand with –NH_2_, –SH, and –COOH coordination sites	[270]
23	AA-5052 alloys	Alkaline Al-air batteries	4M NaOH	8HQ (8hydroxyquinoline) + ZnO	10.0 mM 8HQ + 4.0 mM ZnO	*i*_corr_ reduced from 77.3 to 22.0 mA cm^−2^ (~ 71.6%)	s) R_p_ increased 3 times, and the H_2_ evolution greatly reduced t) Improved energy density, discharge voltage, capacity density, and anode utilization u) 8-HQ adsorbs and forms a chelating complex	[271]
24	Al-1080 alloy	Alkaline Al-air batteries	4M KOH	Hybrid inhibitor (1, 10-decanedithiol (DD); Decyl glucoside (DG), and K_2_SnO_3_ (potassium stannate)	0.05 mM K_2_SnO_3_ + 400 ppm DD + 2 mM DG	H_2_ evolution from 0.2095 to 0.0406 mL cm^−2^ min^−1^ (80.62%)	q) A multicomponent system works synergistically r) The system increases battery capacity, energy density, and recycling v) C_R_ and HER were suppressed, and the contact angle was increased	[273]
25	High-purity Al	Alkaline Al-air batteries	4M NaOH	TSE (*Toona Sinensis* extract)	0.4 g L^−1^	51.9%	k) Self-corrosion and HER were significantly reduced l) R_p_ increased from 1.44 to 2.55 Ω cm^2^ w) Phytochemicals form Al-N and Al-O complexes	[274]
26	Al (bare)	Alkaline Al-air batteries	4M NaOH	N-doped C-quantum dots (Tannic acid and L-tryptophan-based)	150 mg L^−1^	68.4%	m) N-CQDs adsorb through π-Al and N-Al and inhibit corrosion n) Adsorption followed physicochemisorption x) Adsorption reduced H_2_ evolution	[275]
27	Pure Al (99.99%)	Alkaline Al-air batteries	4M NaOH	Sophora japonica leaves (SCDs) based C-quantum dots (SCDs)	0.2 g L^−1^	41.5%	s) Increased R_ct_ and decreased i_corr_ values t) SCDs adsorb by forming S-Al and O–Al coordinate bonds e) Reduced anodic dissolution and HER	[276]
28	Pure Al (99.99%)	Alkaline Al-air batteries	PAA hydrogels with KOH solute	Prussian blue (PB)	12 mg cm^−2^	81.2%	f) PB forms conductive and stable protective film, preventing the formation of Al(OH)_3_ g) Increased anode efficiency, energy density, battery capacity, and power density	[277]
29	Al–Mg-Sn-Gd-P alloy	Alkaline Al-air batteries	1 M KOH	Li^+^, Na^+^, K^+^ and Rb^+^	1 M (LiOH, NaOH, KOH, and KbOH)	Best efficiency for KbOH	o) They all form surface protective oxides that block the diffusion of corrosive species p) Larger size decreases water activities h) C_R_ and H_2_ evolution decrease as the size increases	[278]
30	Al-5052 alloy	Alkaline Al-air batteries	4 M NaOH	ZK5 (maltoheptaose)	1 × 10^–3^ M	60.9%	q) Smother surface reduced C_R_ and H_2_ evolution r) Forms the Al-O complex and becomes effective by surface adsorption s) ZK5 serves as a mixed-type inhibitor	[279]
31	High purity Al (99.999%)	Alkaline Al-air batteries	4 M NaOH	L-cysteine	0.5 M L^−1^	i_corr_ reduced from 2.27 × 10⁻^2^ to 7.86 × 10⁻^3^ μA cm^−2^	i) Cys increases anodic activity and forms a protective film j) –SH, –NH_2_, and –COOH serve as adsorption centers k) Al_2_O_3_ was replaced by hydrated Al(OH)_3_	[280]

**Table 8:** A summary of some recent reports on corrosion inhibition of Al anode using elemental alloying

S/N	Anode alloy	Battery type	Battery Electrolyte	Alloying element and ratio	Efficiency	Salient features	Refs
1	Al-1 Mg-1Zn-0.1Bi-0.02In alloy	Al-AgO battery	7 M KOH and 4 M NaOH	Mg (1 wt.%), Zn (1 wt.%), Bi (0.1 wt.%), In (0.02 wt.%)	C_R_ reduced from 17.162 to 4.273 mg cm^−2^ h^−1^	d) Bi and In provide activation to Al by segregation and oxide disruption e) Zn raises the potential of hydrogen evolution f) Mg reduced cracking and increased strength	[281]
2	Al–Mg-Sn-Ga-In alloy	Alkaline Al-air batteries	2 M NaCl and 4 M NaOH	0.05 wt.% In	*i*_corr_ reduced from 4.1 to 2.9 mA cm^−2^	g) Decreased C_R_ and H_2_ evolution h) Alloying improves electrochemical activities in NaCl but decreases them in NaOH i) Cl^−^ ions adsorb on the surface and stabilize anodic dissolution	[282]
3	Pure Al and Al 7075 alloys	Al-AgO battery	1 M NaOH	Cu-deposited	Self-corrosion decreased from 1280 to 766 mm/y	j) Cu layer serves as a barrier for alkaline attacks k) The galvanic couple of Al-Cu stabilizes dissolution l) Reduced C_R_ and H_2_ evolution and increased energy density and resistance	[283]
4	Pure Al (4N6)	Alkaline Al-air batteries	4 M KOH	NA	C_R_:10.86 mg cm^−2^ h^−1^	a) Lowest i_corr_ (16.8 mA cm^−2^) and highest R_ct_ (2.79 Ω cm^−2^)	[284]
5	Al-Sn	Alkaline Al-air batteries	4 M KOH	0.1 wt.% Sn	C_R_: 5.78 mg cm^−2^ h^−1^	a) Sn improves activity b) Promote segregation and local pitting corrosion c) Retards H_2_ evolution	[284]
6	Al–Mg	Alkaline Al-air batteries	4 M KOH	1.05wt.%Mg	C_R_: 1.25 mg cm^−2^ h^−1^	a) Best activity with the highest R_ct_ value b) It was associated with the lowest surface energy	[284]
7	Al–Mg-Sn	Alkaline Al-air batteries	4 M KOH	1.06 wt.% Mg and 0.09 wt.% Sn	C_R_: 3.06 mg cm^−2^ h^−1^	m) Moderate corrosion rate n) Reasonable corrosion inhibition	[284]

### Electrochemical Corrosion and Corrosion Inhibition of Sustainable Magnesium-Based Batteries

Magnesium (Mg) is a highly reactive metal, and it readily oxidizes to form a passivating film of Mg(OH)_2_, reducing electron transport and electrochemical stability [285, 286]. Corrosion can adversely affect the retention capacity, H_2_ evolution, and self-discharge, which compromise the safety and integrity of batteries [113]. These adverse effects are more pronounced in aqueous, humid, or saline conditions [287, 288]. The corrosion mitigation of Mg anode has already gained significant advancement through corrosion inhibitors, anticorrosive coatings, and alloying approaches [289, 290]. These strategies have been proven essential for achieving reasonable electrode stability, enhancing battery performance, corrosion resistance, and prolonging cycle life. Deyab noticed that the presence of 2.5 mM of DG (decyl glucoside) in 3.5% NaCl (saline) solution reduced the corrosion rate significantly and showed the inhibition efficiency of 94% [291]. DG forms strong, compact inhibitive films via Mg-O bonding, thereby improving anode utilization, discharge voltage, and retention capacity. A binary mixture of 10 mM Na_3_PO_4_ and 30 mM NaF manifests 98.8% efficiency for the Mg anode in 2 M Na_2_SO_4_ [292]. Na_3_PO_4_ and NaF form protective films of Mg_3_(PO_4_)_2_ and MgF_2_, which delay the voltage drop and reduce self-corrosion. The inhibition potentials of phosphate additives for Mg corrosion have been reported in other studies [293, 294].

**Fig. 22:**
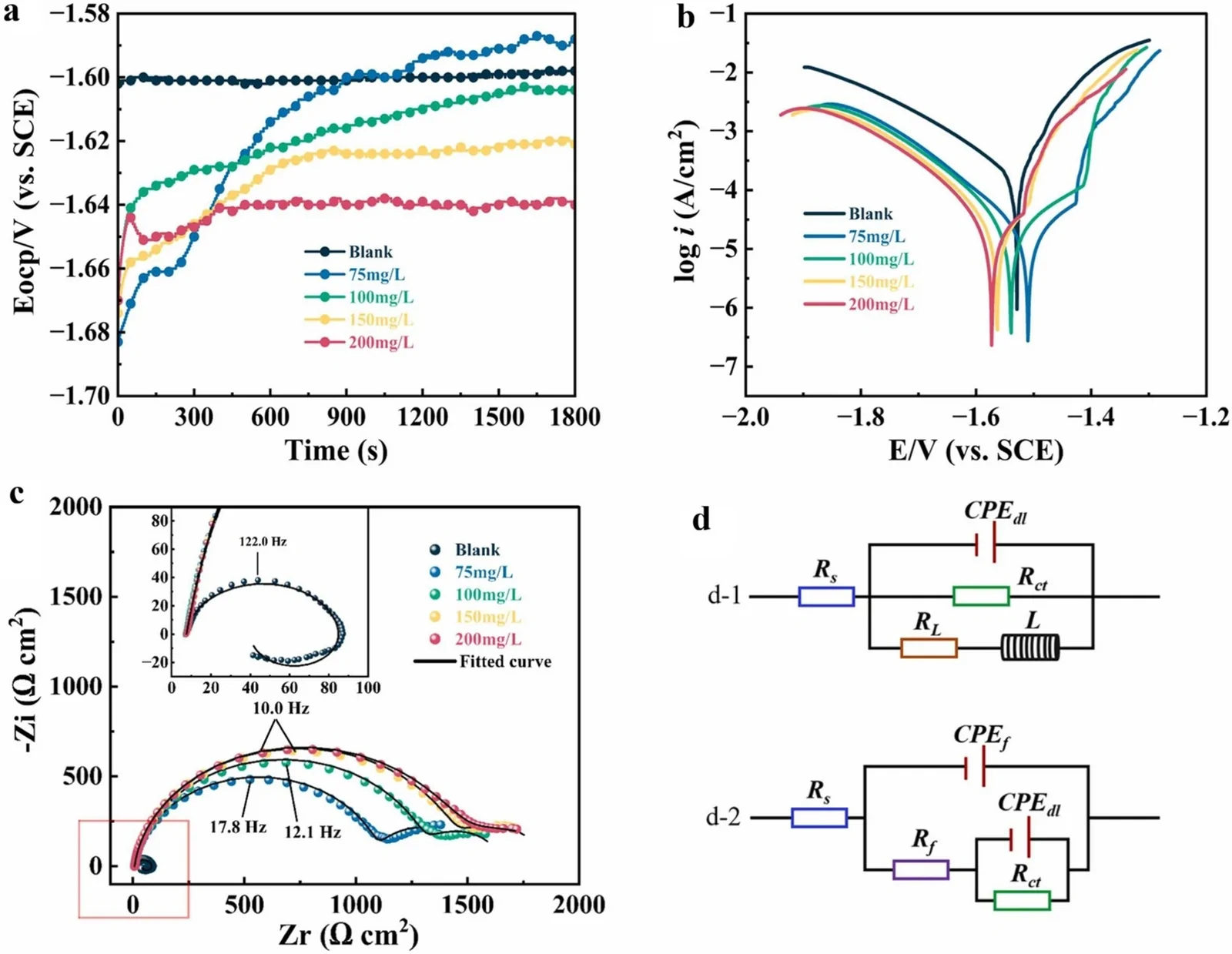
Schematic illustration of AZ31B anode corrosion in 3.5% NaCl with different P-CD levels using **a** OCP, **b** Tafel, **c** Nyquist, and **d** equivalent circuit models [295] (Reproduced with permission. © 2025 Elsevier)

Zhao et al. reported the inhibition effectiveness of P-doped carbon QDs (P-CDs) for Mg-air batteries [295]. Electrochemical analyses revealed that P-CDs significantly reduced the *i*_corr_ value, achieving an inhibition efficiency of 85.2%. The formation of protective films of Mg(OH)_2_ and Mg_3_(PO_4_)_4_ reduced both anodic dissolution and cathodic H_2_ evolution, resulting in a change in the shape of the Tafel polarization curves and Nyquist and Bode EIS plots (Fig. 22). In the presence of P-CDs, the battery showed a 70% increase in the anodic utilization. Jiang et al. studied and reported the use of sodium citrate (NaCA) to improve corrosion resistance and discharge property of Mg-based anodes in a chloride-free electrolyte [296]. As conventional NaCl-based electrolytes increase anodic dissolution and decrease efficiency due to the formation of Mg(OH)_2_ and aggressive Cl^−^ ions, the authors propose using 0.1 M NaCA as an electrolyte. In such electrolytes, Mg anodes can be stabilized by the formation of a protective chelating complex by Mg^2+^ ions.

The corrosion resistance and battery performance of the Mg anodes can be significantly enhanced by alloying. Numerous alloying elements, including Sn, Pb, Al, Ge, Bi, Zn, In, and Ca, significantly reduced self-corrosion and H_2_ evolution, while enhancing anodic efficiency. Wang and coworkers showed that the addition of 1–3 wt.% Sn to AZ61 Mg alloys reduced H_2_ evolution and refined grains, proving improved discharge efficiency and corrosion resistance [297]. Another study showed that as serves as a cathodic poison, retarding H_2_ evolution in Cl^−^ environments [298]. Pb and Al alloying improved electrochemical stability and discharge activity [299]. The presence of 14 and 15 elements, including Sn, Ge, Bi, Sb, and Pb, reduces corrosion rates via cathodic activation [300]. Ge and Zn enhanced the mechanical strength and corrosion resistance of Mg alloys [301]. Ca improves specific energy, cell voltage, and corrosion resistance [302, 303]. Summaries of some representative results showing the effects of corrosion inhibitors and alloying on Mg anode dissolution are presented in Tables 9 and 10.

**Table 9:** A summary of some recent reports on corrosion inhibition of Mg anode using corrosion inhibitors

S/N	Anode material	Battery type	Battery electrolyte	Corrosion inhibitor(s)	Inhibition properties		Salient features	References
					Conc	Efficiency		
**1**	Pure Mg (99.99%)	Mg-air battery	3.5% NaCl	DG (Decyl glucoside)	2.5 mM	94.2%	a) i_corr_ decreased, and R_p_ increased b) DG forms a compact hydrophobic film by coordinating with Mg to form a Mg-O bond c) IR peaks for C–O–C and –O–H confirm the adsorption	[291]
**2**	AZ31 Mg alloy	Primary Mg-MnO_2_ battery	2 M Na_2_SO_4_	Na_3_PO_4_ + NaF (binary mixture)	10 mM Na_3_PO_4_ + 30 mM NaF	98.8% (EIS)	d) R_p_ increased more than 81 times e) Developed MgF_2_ and Mg_3_(PO_4_)_2_ protective layers, preventing self-corrosion f) Improved battery performance	[292]
**3**	AZ31 Mg alloy	Mg-air battery	0.6 M NaCl	Vanadate and phosphate	0.02 M of each	i_corr_ reduced from 79.43 to 1.98 μA cm^−2^	g) *i*_corr_ decreased, and R_p_ increased h) 65.5% increase in anodic efficiency i) Phosphate forms Mg_3_(PO_4_)_2_ and Mg(OH)_2_ compact layer j) Vanadate forms V_2_O_3_ + Mg(OH)_2_ porous, cracked and less protective films	[293]
**4**	AZ31 Mg alloy	Mg-air battery	3.5% NaCl	Sodium phosphate (Na_3_PO_4_) and SDBS	0.5 g L^−1^ Na_3_PO_4_ + 0.5 g L^−1^ SDBS	C_R_ reduced from 0.063 to 0.003 g cm^−2^ h^−1^ (95.2%)	k) Na_3_PO_4_ forms a strong protective covering of Mg_3_(PO_4_)_2_ l) SDBS adsorbs on Mg_3_(PO_4_)_2_ and develops a hydrophobic film for the diffusion of Cl^−^ m) Improved potential stability and decreased byproduct formation	[294]
**5**	AZ31B Mg alloys	Neutral Mg-air battery	3.5% NaCl	PCDs (phosphorus-doped carbon quantum dots)	200 mgL^−1^	*i*_corr_ reduced from 172.6 to 25.5 μA cm^−2^ (~ 85.2%)	n) P-CDs retard anodic dissolution and cathodic H_2_ evolution o) Battery performance was significantly improved p) P-CDs adsorb and form corrosion-inhibitive films (Mg_3_(PO_4_)_2_ and Mg(OH)_2_)	[295]
**6**	Mg0.5-Zn0.2-Ge alloys	Aqueous Mg-air battery	3.5% NaCl	Sodium citrate (NaSC)	0.1 M (NaSC)	Utilization efficiency improved by 91.4%	q) Decreased self-corrosion (~ 50%), H_2_ evolution, and film resistance (3 times) r) Reduced passivation and byproducts formation s) NaSC forms a soluble complex with Mg^2+/^ Mg surface	[296]

**Table 10:** A summary of some recent reports on corrosion inhibition of Mg anode using elemental alloying

S/N	Anode alloy	Battery type	Battery electrolyte	Alloying element and ratio	Efficiency	Salient features	References
1	AZ61-Sn alloys (AZ61: Mg-6Al-1Zn)	Seawater-based Mg-air battery	3.5% NaCl	Sn (1–3 wt.%)	i_corr_ reduced from 1.0 × 10^–1^ to 1.58 × 10^–1^ mA cm^−2^	o) Shifts *E*_corr_ in the positive direction, reduced H_2_ evolution, and improved current efficiency p) The best performance was derived at 3.0 wt.% q) Mg anode manifests uniform corrosion	[297]
2	Pure Mg and Mg-As (0.37wt.%) alloys	Seawater-based Mg battery	0.1 and 2.0 M NaCl	As (0.3 wt.%)	C_R_ was reduced 5 times	a) As serves as a strong cathodic poison b) Prevents cathodic polarization and retards H_2_ evolution (10 times). Shift *E*_corr_ to the negative side c) Increase charge efficiency and corrosion resistance	[298]
3	Mg–Al-Pb alloys (Mg-3-9Al-2.5–7.5 Pb)	Seawater-based Mg battery	3.5% NaCl	Pb + Al alloying additives	3, 6, and 9.0 wt.% Al and 2.5, 5.0 & 7.5wt. Pb	d) The alloying elements synergistically improve battery and corrosion resistance performance e) Passivation film of Mg(OH)_2_ forms to provide protection	[299]
4	Pure Mg and Mg-X alloys (x: Bi, Ge, Sn, Pb, and Sb)	Mg primary battery	0.1 M NaCl	Bi, Ge, Sn, Pb, and Sb (group 14 and 15 elements)	C_R_ reduced by ~ 1 order of magnitude	f) 0.3 wt.% Ge provided the best corrosion resistance g) Mass loss and H_2_ evolution were suppressed with no filiform corrosion h) Mg-0.3Ge sample showed the best resistance	[300]
5	Mg-1Zn-xGe alloys (x: 0, 0.3 and 0.5 wt.%)	Mg primary battery	0.1 M NaCl	Ge with low Zn (1.0 wt.%)	C_R_ decreased nearly 10 times	r) No filiform corrosion with reduced H_2_ evolution and HER s) Inhibits anodic polarization and cathodic activation t) Improve mechanical strength and corrosion resistance	[301]
6	Mg-Ca binary alloy	Mg-air battery	3.5% NaCl	Ca (01 wt.%)	*i*_corr_ reduced from 84.1 to 63.0 μA cm^−2^	i) Low Ca concentration favors protection, but not high concentration j) Mg_2_Ca provides detrimental galvanic effects u) The battery performance, including discharge potential, efficiency, specific capacity, and self-corrosion, was greatly improved	[303]
7	Mg-Sn binary alloys	Mg primary battery	0.6 M NaOH	1.5 mM	95.1% (10 Sn)	v) Sn increases corrosion and polarization resistance w) 10SN forms a strong barrier film with the lowest *i*_corr_ value x) Sn also decreased cathodic polarization, H_2_ evolution, and HER	[302]

### Miscellaneous: Sustainable Na/K-Ion, Solid-State, Recyclable Li-Ion, and Organic Batteries

The practical implementation of sodium-ion batteries (SIBs) and potassium-ion batteries (KIBs) in energy storage is greatly challenged and hindered by undesirable corrosion [304–307]. Due to their weaker Lewis acidity, greater ionic radii, and unique solvation structures, Na^+^ and K^+^ exhibit different interfacial chemistries compared to Li^+^, despite sharing numerous electrochemical properties. The literature inspection shows that the cycling instability of SIBs and KIBs is mainly caused by interfacial corrosion of the hard carbon anodes and layered oxide cathodes [308, 309]. Unlike Li-ion batteries, Na/K systems reveal more aggressive electrolyte-mediated decomposition due to narrower electrochemical stability windows [310, 311]. This makes corrosion mitigation an essential design requirement in SIBs and KIBs. In layered potassium transition metal oxides, including P′3- and P3-type K_x_MnO_2_, they represent a beautiful class of materials owing to their favorable K + diffusion kinetics, cost-effectivity, electrochemical reversibility, and exceedingly high theoretical capacity [312]. Nevertheless, their electrochemical stability and reversibility were greatly hindered in conventional ester-based electrolytes, especially in the presence of KPF_6_, due to adverse interfacial reactions.

PF_6_^−^ ions induce oxidative instability and hydrolysis by producing HF and several other corrosive species that aggressively attack Mn–O frameworks, causing lattice distortion, Mn disintegration and dissolution, rapid voltage decay, and structural stress [313]. More so, in carbonate-based electrolytes, the solvent molecules can intercalate into the layered oxide galleries and destabilize the structure by producing thick resistive cathode–electrolyte interface (CEI) layers [314]. The combined action of solvent diffusion and HF-mediated dissolution constitutes a severe, irreversible corrosion pathway that undermines the electrode's integrity and exacerbates its losses. In F-modified KMOF-7 and related systems, the incomplete replacement of O^2−^ by F^−^ ions enhances interfacial stability and retards HF attacks by minimizing CEI overgrowth and solvent ingress. This represents a practical, cost-effective cathode-side corrosion mitigation that is highly compatible with the high-voltage K-ion battery. Hard carbon (HC) has emerged and established itself as one of the most viable anode materials for Na/K batteries due to its low cost, tunable porous structure, and compatibility with the larger ions [315–318]. However, as HC surfaces are highly reactive, their performance is significantly influenced by the nature and stability of the solid-electrolyte interphase (SEI).

In KPF6/carbonate-based electrolyte systems, at low potential, electrolyte decomposition produces highly unstable SEI layers that fracture and lead to continuous consumption of electrolyte and battery capacity fading [318]. KPF6/DME and other ester-based electrolytes form unstable SEIs, leading to interfacial corrosion. Conversely, KFSI-based electrolytes form KF-rich, inorganic SEI films, enhancing anode stability by reducing solvent reduction and thereby maintaining cycle life and coulombic efficiency. The solvent-mediated corrosion in these battery systems can be minimized by implementing synergistic mitigation approaches, including anion-engineered salts [318], surface fluorination [313], polyanionic frameworks [309], nitrate stabilizers [319], aqueous gels [320], solid-state designs [321], and optimized hard carbon [308]. The literature studies show that electrolyte engineering provides an effective pathway for corrosion mitigation [322–325]. A shift from conventional KPF6/carbonate-based electrolytes to advanced electrolytes can reduce solvent decomposition, stabilize the SEI/CEI layer, and enhance current-collector passivation [326]. The use of binary salt electrolytes offers an effective method for corrosion control in KIBs. In KPF_6_/KFSA carbonate electrolytes, a co-salt, KFSA (K[N(SO_2_F)_2_]), is introduced, and its anion decomposes more rapidly than PF6-, producing F- and S-containing species that precipitate in SEI formation [323].

The mismatch in electrochemical potential, mechanical properties, and electronic structures between electrodes and solid electrolytes leads to electrochemical corrosion in sustainable solid-state batteries [327–330]. Unlike liquid electrolyte-based systems, which are associated with wet surfaces and maintain dynamic contact, solid interfaces generally accumulate stress, space-charge layers, and micropores/microcracks that accelerate corrosive reactions and increase interfacial resistance. In solid-state battery systems, high-voltage cathodes can oxidize halide, polymer, or sulfide-derived electrolytes. At the same time, alkali metal anodes can reduce hybrid or oxide electrolytes, forming brittle byproducts that can promote electrode degradation [331, 332]. These undesirable reactions are particularly problematic in sustainable solid-state battery systems that utilize bio-derived polymers, recyclable materials, or low-toxic ceramics, as these materials possess reactive surface groups and exhibit narrower stability windows [333–335]. Interfacial corrosion in solid-state batteries has been identified as a primary failure mechanism that adversely affects their safety, integrity, and cycle life in next-generation alkali-ion battery systems [336, 337]. Corrosion mitigation of electrodes in solid-state batteries has been significantly advanced by engineering the ion-conductive interfaces. One of the most effective approaches to corrosion mitigation in such systems is the use of thin anticorrosive coatings, including metal oxides, fluorides, and phosphates. The coatings shield the electrolyte from attacks and minimize the corrosion reactions [338–340]. Polymeric buffer layers and artificial interphases can be used to accommodate strain, maintain continuous contact, and mitigate crack propagation by retarding the formation of interfacial voids and fragmentation, thereby slowing corrosion reactions [341]. Simultaneously, electrolyte-centered approaches, such as anion substitution, aliovalent doping, and polymer blending, offer alternative pathways to mitigate electrode degradation and enhance electrochemical stability by reducing corrosive interactions [342–344].

In Li-ion batteries (LIBs), electrochemical corrosion initiates from the destructive reactions at the electrode (Li)–electrolyte interfaces [345, 346]. These reactions include reduction of electrolyte, oxidation of electrolyte at the cathode, and current-collector attacks, transition metal dissolution, and destabilization of SEI and CEI [45]. These corrosion mechanisms are greatly accelerated and intensified by moisture-induced LiPF6 hydrolysis, thermal stress, and high voltage, as these processes accelerate the formation of HF and interphase breakdown [347–349]. Continuous corrosion leads to lithium loss, cracking, and thickening of the SEI and CEI, resulting in successive capacity decay and increased cell impedance. The cathodic corrosion can be directly linked to anodic instability [89, 350]. These challenges can be effectively addressed by implementing appropriate corrosion-mitigating strategies that minimize electrode–electrolyte interactions. External additives, including vinylene carbonate (VC), lithium difluoro(oxalato)borate (LiDFOB), and fluoroethylene carbonate (FEC), accelerate the formation of mechanically stable SEI/CEI layers that minimize the metal dissolution from high-voltage cathodes [351–353]. Similarly, protective coatings of metal oxides, such as ZrO_2_ and Al_2_O_3_, niobate, and phosphates, provide physical barriers for electrolyte attacks and mitigate dissolution [354, 355]. Highly concentrated electrolytes, especially fluorinated ones, are associated with reduced HF production, promoting uniform interphase, cycle life, and anodic stability [356, 357]. The corrosion mitigation in recyclable Li-ion batteries is significant as it not only enhances the lifetime for operation but also reduces contamination, enhances structural retention, and enables effective materials recovery in second-life uses [358, 359].

**Fig. 23:**
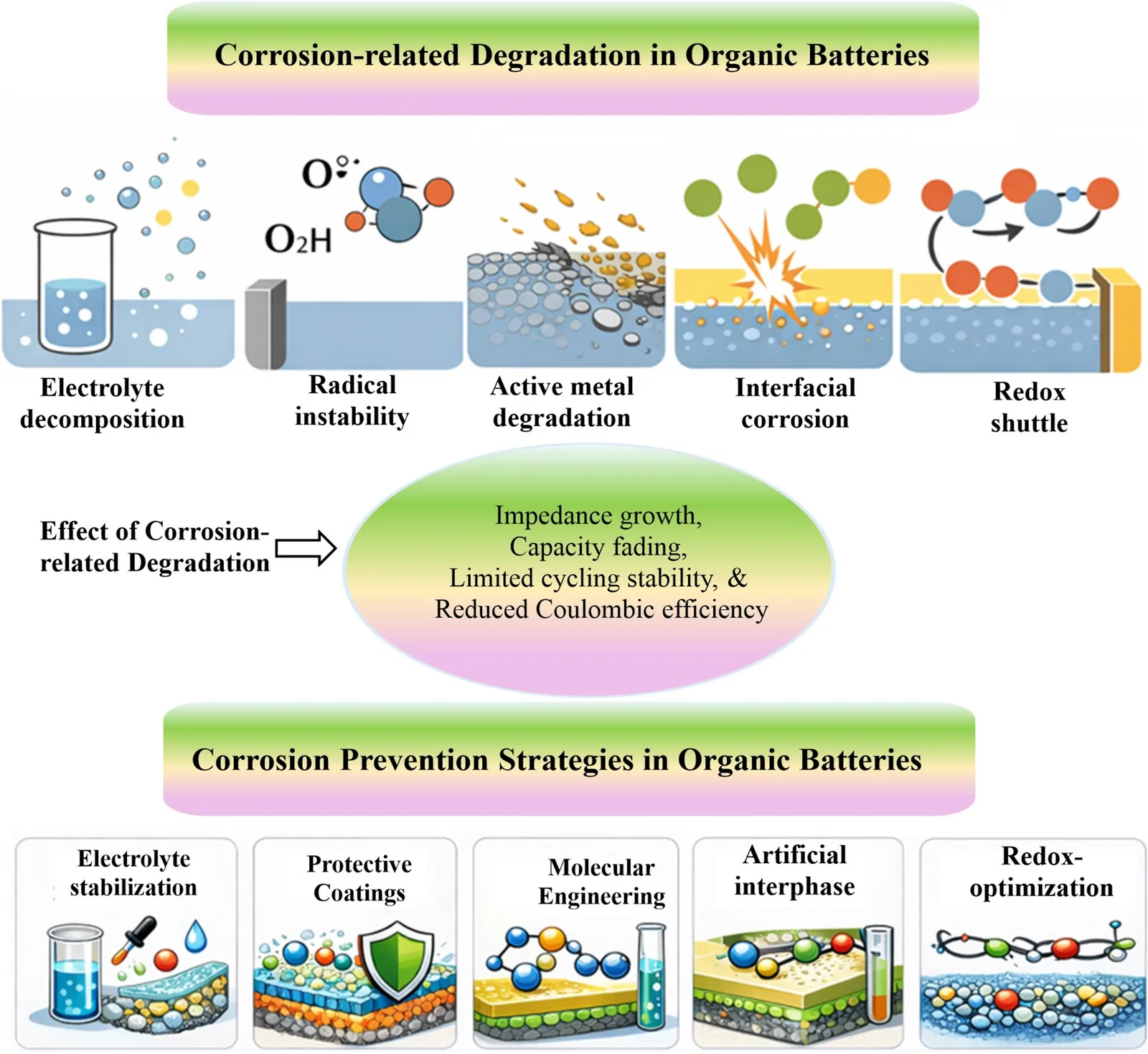
Diagram showing pathways of corrosion-related degradation in organic battery systems and various corrosion prevention strategies. The upper panel shows the central corrosion mechanisms, and the lower panel shows the prevention strategies in such systems to suppress corrosion-mediated degradation and improve electrochemical stability

In organic batteries, corrosion originates from redox and chemical reactions that attack metallic current collectors and organic electrode frameworks [360–362]. Organic electrodes, including Schiff base polymers, imides, conjugated redox polymers, and quinones, are susceptible to radical fragmentation, degradation from continuous charge/discharge cycling, protonation, and nucleophilic attack [363]. These reactions trigger the formation of anion radicals, enhanced electrolyte decomposition, and destabilization of the SEI and CEI, particularly when less stable salts or reactive solvents are employed [364]. For example, carbonyl (>C=O)-rich organic cathodes may undergo ring-opening or enolization reactions, while dissolved radical intermediates can diffuse into the electrolyte, resulting in rapid capacity fading and material degradation [365, 366]. The breakdown of organic electrodes, especially at high voltages, can produce aggressive species, such as HF, which can attack the current collectors and compromise their structural and interfacial integrity [45, 367]. These degradation processes lead to increased polarization, reduced cycle life, destruction of structural and interfacial stability, and decreased coulombic efficiency, despite their eco-friendly behavior [368]. Owing to these adverse effects and processes, corrosion mitigation has been essential in sustainable organic batteries (Fig. 23). Unlike corrosion in metal-anode systems, degradation in organic batteries mainly manifests through electrochemical reactions and chemical instability of redox-active molecules. Figure 23 schematically illustrates the major degradation pathways, including interfacial side reactions, active metal dissolution, electrolyte instability, and radical decomposition. These processes are directly involved in several adverse effects, including impedance growth, capacity fading, limited cycling stability, and reduced Coulombic efficiency. Therefore, different corrosion prevention strategies focus on electrolyte engineering, suppression of active metal corrosion, molecular-level stabilization, and the design of artificial interphases to enhance chemical and electrochemical stability. Molecular designing, especially hydrogen-bond stabilization, π-extension, adding of electron-withdrawing substituents, and polymer backbone cross-linking, minimizes the solubility of the materials and retard degradation [369–371]. Meanwhile, the implementation of corrosion-resistant salts, such as FSI- and TFSI-, localized high-concentration and highly concentrated electrolytes, water-in-salt formulations, and ionic liquids, can improve corrosion and degradation resistance [372–374]. The diffusion of electrolytes and subsequent corrosive attacks can be further minimized by using anticorrosive protective coatings [375, 376].

## Next-Generation Corrosion Resistance Materials and Strategies: Self-Healing, Smart, and Nanostructured Materials

**Fig. 24:**
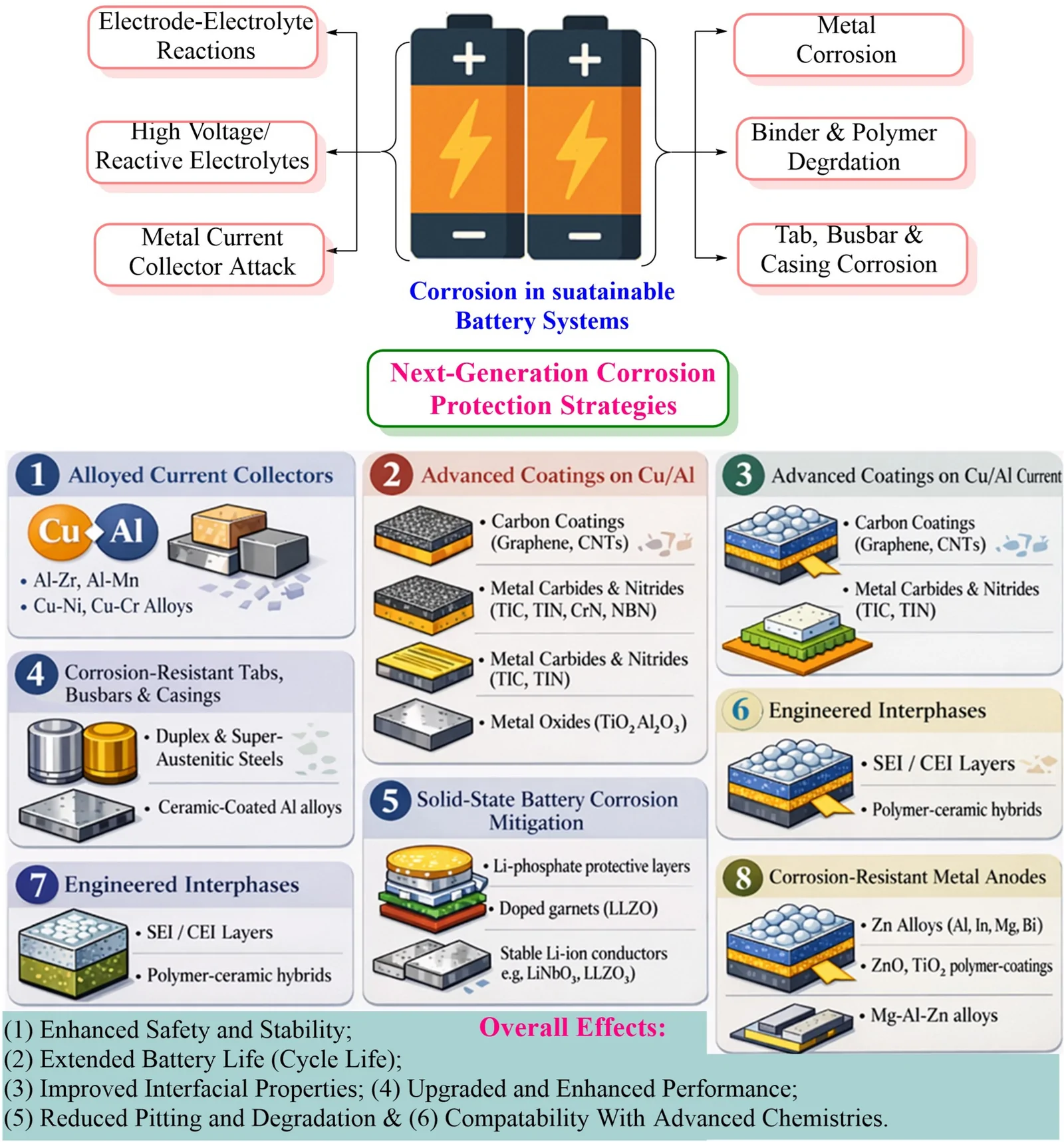
Schematic illustration of attempts made toward the development and implementation of next-generation corrosion-resistant materials for battery applications

Corrosion in batteries is a complex and broader process that not only includes metal rusting but also electrode (anode/cathode)–electrolyte interactions and degradation, reactions between electrode and current collectors, binder and polymer degradation, degradation of cooling plates, tabs, and cell casing, and high voltage/temperature corrosion [49]. Corrosion challenges are particularly severe in modern energy storage systems that operate at relatively high voltages and use aggressive electrolytes [377, 378]. The modern energy storage systems also include replacing traditional Fe, Cu, Al, Zn, and Ni-based electrodes and chemistries that are reaching their limits. Recently, several attempts have been made to develop and implement next-generation corrosion-resistant materials for battery applications (Fig. 24). One of the major attempts involves alloying Cu and Al current collectors with different elements [379, 380]. The literature shows that Al–Zr, Al–Mn, or Al–Mg–Si alloys exhibit superior passive film stability and enhanced pitting resistance in LiPF6 electrolytes [381–383]. The presence of alloying elements enhances the stability and adherence of fluoride or oxide films. Similarly, micro-alloyed steels, such as Cu–Ni and Cu–Cr, possess better corrosion resistance and mechanical stability. The tailored microstructures and presence of alloying elements enhance passivity, facilitating the repair of local breakdowns.

Carbon coatings on current collectors (Cu or Al) are emerging attempts to enhance their corrosion resistance [384, 385]. The carbon coatings, including graphene (G), carbon nanotubes (CNTs), and amorphous carbon on Al or Cu, enhance their chemical inertness, electrical conductivity, and serve as a physical barrier against oxidative and HF attacks [386–388]. Metal carbide (MC) and metal nitride (MN) coatings, such as TiC, CrN, TiN, and NbN, improve the hardness and corrosion resistance of the current collectors [389, 390]. These coatings can be applied by atomic layer deposition (ALD) or physical vapor deposition (PVD). Coatings with conductive polymers, such as poly(3,4-ethylenedioxythiophene) (PEDOT) or related polymers, improve flexibility, adhesion, and corrosion resistance [391, 392]. The presence of metal oxides such as TiO_2_ and Al_2_O_3_ provides additional pitting resistance, especially at high potentials. Instead of traditional tabs, busbars, and cell casings that utilize aluminum, copper, and nickel-plated steel cans, high-performance steels such as super-austenitic or duplex steels can be used [393]. These next-generation materials exhibit high resistance to HF, chloride, and pitting attacks.

Ceramic coatings improve the chemical resistance and mechanical properties of Al alloys [394, 395]. Alloying also improves the corrosion resistance of tabs and busbars. Artificial interphases and protective interfaces enhance material stability by modifying interfaces rather than bulk material. These phases and faces retard the direct contact of metals with aggressive electrolytes and allow the ion transport to occur freely. These include cathode-electrolyte interphases (CEI), solid-electrolyte interphases (SEI), or polymer-ceramic hybrids [396, 397]. They are nanometer-thin and significantly improve interfacial stability and corrosion resistance. In solid-state batteries, corrosion problems arise from oxide- and sulfide-based electrolytes that react with Li metal and with oxygen in air. Recently, the use of Li-phosphate protective layers, doped garnet, and stable Li-ion conductors has emerged as non-corrosive and safe electrolytes [398]. They improve interfacial resistance, reduce electrochemical decomposition, and enhance chemical stability.

Pristine metal anodes are highly susceptible to corrosion, particularly through passivation, oxygen evolution, and hydrogen evolution. The next-generation approach involves allowing suitable elements to pass through. For example, Al-, In-, Mg-, and Bi-based alloying of the Zn anode minimizes H_2_ evolution and dendrite growth and increases corrosion resistance [238, 399]. The corrosion resistance of Zn anodes can also be improved by applying inorganic, organic, or polymer coatings. Allowing Mg with Al and Zn (Mg–Al–Zn) improves corrosion resistance without decreasing the electrochemical activities. Polymer components, such as separators, binders, adhesives, and gaskets, also undergo severe degradation, although attention has been focused on metallic corrosion. Therefore, suitable tailoring of these components can improve the lifespan of batteries by retarding the risks of corrosion. For example, cross-linked or fluorinated binders exhibit remarkable resistance to pitting attack at high-voltage cathodes [400, 401]. Likewise, ceramic-coated separators reveal resistance against oxidative degradation and improved thermal and mechanical stability [402].

**Fig. 25:**
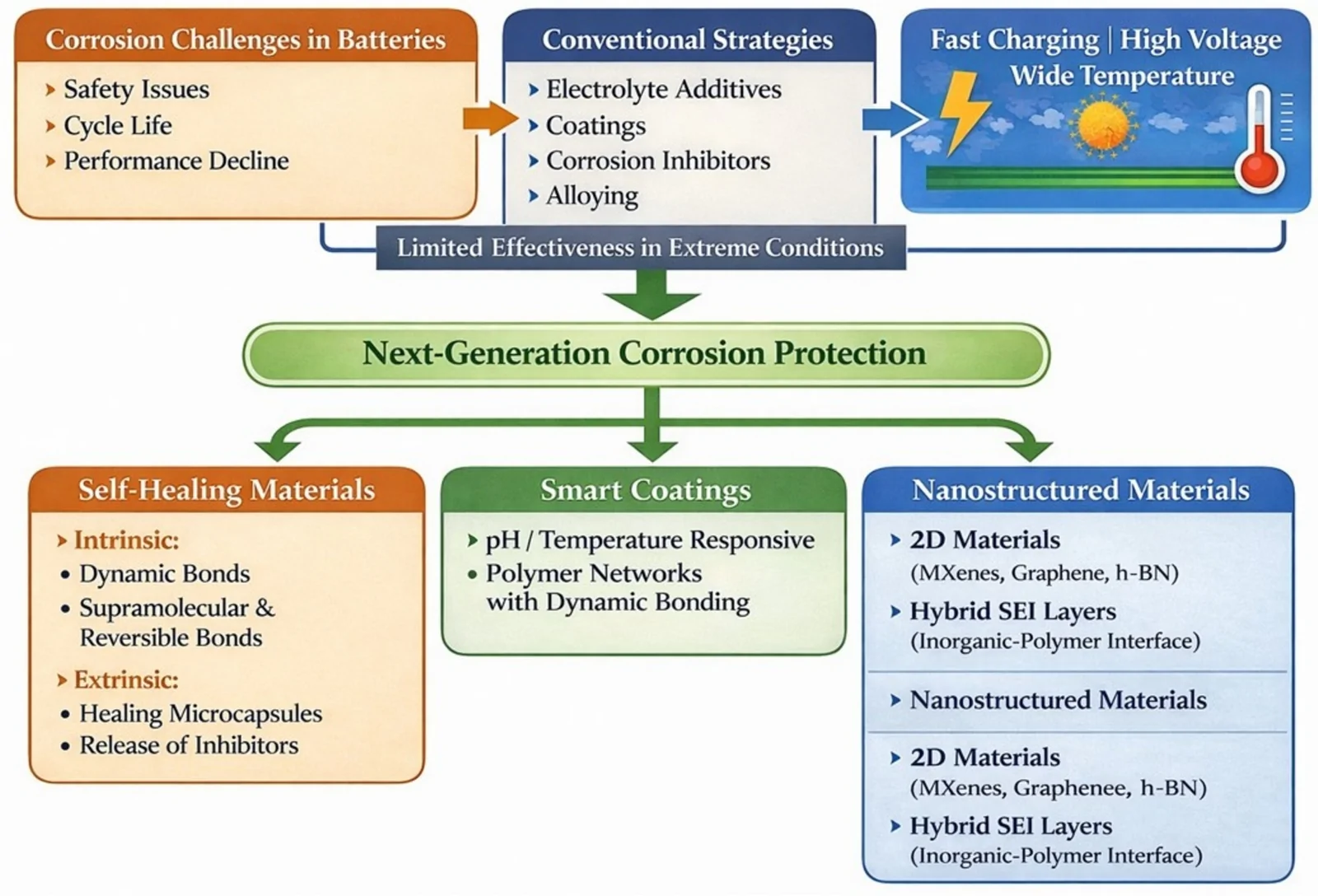
Schematic illustration showing next-generation corrosion protection strategies in modern-day battery systems

The ongoing discussion reveals that corrosion has been a significant challenge and a notable barrier to the development of sustainable batteries. As corrosion adversely affects the safety, cycle life, and performance of the battery, several strategies have been developed to mitigate the consequences [403]. Conventional strategies, such as electrolyte additives, coatings, corrosion inhibitors, and alloying, are largely inadequate in modern-day battery systems that operate under relatively extreme conditions, including fast charging, high voltage, and wide temperature ranges. These demand next-generation corrosion protection strategies, including self-healing materials and coatings, innovative or stimuli-responsive systems, and nanostructured architectures in modern-day battery systems (Fig. 25). Self-healing materials are characterized by their ability to restore functional and structural integrity after chemical degradation or mechanical loss at the battery interfaces [404–406]. Self-healing can be of two types: intrinsic and extrinsic. Intrinsic self-healing reversible chemical bonds, including supramolecular interactions, dynamic covalent bonds, and ionic cross-linking, reform spontaneously at the damaged or corroded sites. On the other hand, in extrinsic self-healing, healing agents are encapsulated in micro- or nanocapsules or in polymer networks to become effective at the damage site in response to external stimuli.

Self-healing coatings incorporating suitable healing agents have been used for corrosion protection. In these coatings, corrosion inhibitors such as organic species, cerium salts, and monomers are encapsulated in microcapsules. These microcapsules are stimuli-responsive, i.e., they rupture in response to external stimuli, such as pH and temperature changes, at damaged sites and release active corrosion inhibitors/healing agents to provide self-healing protection [404–406]. In polymer-based self-healing coatings, external stimuli trigger dynamic network changes, including hydrogen and disulfide bonding, to provide instantaneous, automatic healing at damaged sites [407–410]. In traditional batteries, SEI layers are highly unstable and experience electrolyte decomposition and corrosion. However, the next-generation dynamic polymer components automatically repair seal cracks and pores generated during cycling. Hybrid (inorganic-polymer) SEI interfaces, incorporating borate, Li_3_N, or LiF, reorganize under electrochemical stress [411–413]. Recently, the use of nanostructured materials in sustainable corrosion protection in battery environments has been significantly enhanced. Due to their ability to minimize localized reaction sites, enhance surface uniformity, control electron and ion transport, offer tunable surface chemistry, and possess a high surface area, their use in protecting against battery corrosion is gaining particular attention. 2D material-based, including MXenes, graphene, and h-BN, are effectively employed in nanostructured coatings [39, 414–416]. Polymers and ceramic nanocomposites offer synergistic effects that enhance both corrosion protection and flexibility [417–419].

The literature investigation shows that several strategies and corrosion-resistant materials, including surface coatings, corrosion inhibitors, ionic liquids (ILs), bio-based corrosion inhibitors, and alloying, have been extensively employed to provide significant enhancements in corrosion resistance and cycling stability; several inherent shortcomings remain. Most ILs, surfactants, and organic corrosion inhibitors become effective by adsorbing onto the anode surface and forming hydrophobic protective films. Their adsorption and effectiveness may be compromised by competitive adsorption with aggressive ions or by instability under high-voltage, high-temperature conditions. Likewise, though alloying improves corrosion resistance, it may also adversely affect electrochemical potential, increase the risk of galvanic corrosion due to coupling effects, or increase cost. Oxide- and polymer-based protective coatings improve interfacial stability; however, excessive coating thickness may adversely affect ion transport and increase internal resistance. Moreover, though advanced materials such as ILs and MOFs are highly effective at the laboratory scale, they may face challenges in commercial-scale production, long-term stability, economic feasibility, and environmental compatibility. Though next-generation protection strategies, such as nanostructured interphases, self-healing coatings, and multifunctional hybrid systems, offer performance advantages, they still face numerous challenges. These challenges include economic feasibility, synthetic scalability, recyclability, and interfacial stability in real working conditions. Moreso, their complex structure may introduce additional mechanical instability, undesirable complexity, or interfacial resistance over prolonged use.

## Challenges and Future Directions

The ongoing discussion shows that corrosion imposes numerous challenges in the development of sustainable battery systems. These challenges arise from the inherent susceptibility of metallic anodes and current collectors to corrosion via electrochemical reactions. Battery corrosion manifests as galvanic reactions, hydrogen evolution, localized pitting corrosion, and surface passivation. These undesirable processes accelerate the loss of active metal (anode), increase impedance, and reduce the coulombic efficiency of batteries. These processes become particularly destructive in aqueous batteries, where oxygen reduction and water spitting generate extremely corrosive environments. Another serious challenge arises from dendrite formation and growth, which concentrates current at microscopic tips, produces regions of local alkalinity or acidity, and ruptures protective (passive) films. Corrosion also produces recycling and environmental challenges to sustainable battery systems. As the battery ages, mixed-metal deposits, oxides, and hydroxides migrate to the separator membranes and active materials. The contamination reduces battery efficiency by lowering the purity of recovered metals, including Mg, Co, Mn, Al, Fe, Ni, and Li. These impurities also diminish recycling efficiency, increase the amount of spent electrolyte, and increase chemical complexity. Moreover, oxygen-rich electrolytes are associated with high swelling risk, accelerated mechanical damage, and cracking of cell components.

In view of the above, recent studies have focused on addressing the challenges associated with traditional battery systems, offering substantial prospects. Future studies should concentrate substantially on interface engineering to optimize ion-surface interactions, thereby suppressing the corrosion pathway and stabilizing metallic electrodes. Recent reports suggest that the presence of functional additives can positively impact solvation structures, enhance the density and uniformity of passive films, and displace pre-adsorbed water molecules. Numerous bio-based molecules (such as triglycine, chitosan, fucoidan, and carboxymethyl cellulose), multidentate heterocycles (e.g., imidazoles), and chelating ligands (such as sodium gluconate and nitrilotriacetic acid) have been reported as relatively non-toxic inhibitors of hydrogen evolution and dendrite growth. These species provide significant corrosion protection and enhance metal-ion transport kinetics, which are essential for improved battery performance and sustainability. Nonetheless, the available reports on these aspects are limited and warrant further exploration in future studies.

Recently, the research direction in sustainable battery systems is transitioning toward solid-state and hybrid electrolyte systems, which utilize deep eutectic solvents (DESs), ionic liquids (ILs), metal–organic frameworks (MOFs), and polymer-inorganic composites as multifunctional stabilizers. With the rapid progress of artificial intelligence for science (AI4S), data-driven modeling and AI are emerging as powerful tools for understanding corrosion mechanisms and developing subsequent mitigation strategies in battery systems [420–422]. Machine learning (ML) has already been employed to assess stability windows and corrosion rates as a function of pH, anode (alloy) composition, electrolyte chemistry, and temperature, enabling accelerated screening of suitable corrosion inhibitors and corrosion resistance materials [423, 424]. In battery systems, AI-assisted analysis of surface characterization (e.g., SEM, EDS, AFM, XPS, ICP-MS) and electrochemical data (e.g., *R*_ct_, *R*_p_, *E*_corr_, *i*_corr_) can help identify hidden correlations among interfacial properties, long-term degradation, and dendrite formation. In battery systems, data-driven approaches can also be used to optimize the design of corrosion-protective coatings, molecular-level corrosion inhibitors, and electrolyte fabrication [421, 425]. However, as corrosion-related AI datasets are often heterogeneous, small, and generated under non-standardized (unrealistic) testing conditions, models can face challenges when used across different materials and real operating environments.

Several currently used ML models work like black boxes, i.e., they provide the results without providing significant insight into the mechanistic aspects. This makes it hard to fully trust and rely on the models, particularly when they are used in new environments or situations that differ significantly from the data on which they were trained. Therefore, in future investigations, a better approach could be to combine basic scientific principles, such as transport and electrochemical principles, with AI. These models are generally known as hybrid or physics-informed models [426]. The other practical methods include active learning, where developed models are used to determine which experiments to run next, and digital twins, which link real-time experimental data with predictive tools to identify and diagnose corrosion. These electrolytes are well-known for their ability to alter solvation energetics, produce self-healing interfaces, and minimize free water acidity. Thin anticorrosive coatings provide physical shielding and corrosion protection, enabling prolonged cycling stability. The corrosion prevention strategies should be further explored due to the increasing demand for grid storage, seawater-based energy systems, and the use of EV batteries in a second life. Cost-effective production, recycling, and corrosion protection, primarily using green inhibitors, enhance performance and align with global NZE goals.

Several inorganic, organic, and bio-derived corrosion inhibitors have been used in corrosion protection in battery systems, and they manifest remarkable performance at the laboratory scale. However, their large-scale use in battery systems depends on several other factors beyond their protection (electrochemical) performance. Availability of raw materials, complexity in synthesis, cost-feasibility, compatibility with electrolyte composition, influence on ionic conductivity, and long-term chemical stability must be carefully considered. Several functionalized organic corrosion inhibitors, mainly heterocycles, while they exhibit remarkable effectiveness, may involve tedious multi-step syntheses or expensive starting materials associated with several workups and purification steps. These factors not only make synthesis tedious and costly but also adversely affect the environment and industrial scalability. Likewise, though bio-based alternatives can be considered eco-friendly and promising corrosion inhibitors due to their excellent interfacial properties, they may face challenges with long-term stability, especially in highly alkaline or acidic battery conditions and during extended cycling. Moreover, these external additives added as corrosion inhibitors can adversely affect ion transport kinetics, electrolyte viscosity and composition, downstream recycling processes, and separator compatibility.

Synergistic formulations, i.e., combining two or more active species in a single formulation, represent a promising strategy for achieving multifunctional protection in battery systems. Nevertheless, whether their combination has a synergetic or antagonistic effect should be systematically tested. The literature outcomes suggest that synergistic protection is provided by a proper combination of corrosion inhibitors capable of retarding both anodic metal dissolution and cathodic hydrogen evolution or oxygen reduction, resulting in overall mixed-type protection. Hybrid formulations of polymers and chelating organic molecules with inorganic (metal) ions are typical examples of synergistic formulations. On the other hand, antagonistic effects may result from competitive adsorption, excessive surface hydration, complex formation, or destabilization of the inhibitor (passive) film. Therefore, for the practical uses, the priority design principles for effective corrosion inhibitor selection should include: (i) the inhibitor should form effective and strong inhibitive films, (ii) the inhibitor must be stable in the operating pH window, (iii) the inhibitor synthesis should be cost-effective and scalable, (iv) the inhibitor should provide minimum interference with kinetics of ion (Zn^2+^/ Al^3+^, Mg^2+^) transport and (v) the inhibitor should be compatible with environmental and recycling rules and regulations. Therefore, effective formulation should be balanced by electrochemical efficiency, economic feasibility, interfacial stability, and synthesis to enable industry-scale production.

## Conclusions

Corrosion remains one of the most persistent problems, adversely affecting the longevity, safety, efficiency, and sustainability of battery systems. Although sustainable batteries, such as Mg-, Al-, Na-, Zn-, and bio-based systems, offer some advantages in terms of availability, recyclability, and environmental performance, their real-world practical use is strictly hindered by complex corrosion pathways. These corrosion pathways promote degradation reactions, destabilize interfaces, enhance self-discharge, and compromise structural integrity, performance, and cycle life. Corrosion adversely affects energy efficiency, shortens service life, and reduces recycling processes, while also increasing safety risks by contaminating systems with corrosion products and impurities. Recent advancements show that corrosion mitigation strategies can significantly increase sustainable battery performance. These strategies include surface passivating coatings, alloy engineering (alloying), electrolyte optimization and tailoring, and use of multifunctional organic, inorganic, polymeric, ILs, DESs, and bio-based corrosion inhibitors. The corrosion protection strategies reduced hydrogen evolution, oxygen spitting, and electrode dissolution.

Many inhibitors, including PEG, gluconates, CMC, heterocycles, MOFs, and advanced surface-active (amphiphilic) molecules, manifested reasonably high protection efficiency, exceeding 90%. These species align with the principles of green chemistry, exhibiting high biodegradability, computability, and low toxicity, enabling dendrite-free cycling and extending the cycle life of metallic anodes. However, given the demands for high-performance, fully recyclable batteries and corrosion resistance, the current progress appears insufficient. Therefore, research and development on dynamic passivation behavior, electrode–electrolyte interactions, and interface chemistry across various battery environments need to be thoroughly studied and established. Future studies should also integrate rational molecular design, in situ testing, and predictive modeling with experimental approaches to develop and demonstrate practical, eco-friendly corrosion protection strategies.
